# *In-Silico* Study of Brassinosteroid Signaling Genes in Rice Provides Insight Into Mechanisms Which Regulate Their Expression

**DOI:** 10.3389/fgene.2022.953458

**Published:** 2022-07-06

**Authors:** Sunny Ahmar, Damian Gruszka

**Affiliations:** Institute of Biology, Biotechnology and Environmental Protection, Faculty of Natural Sciences, University of Silesia, Katowice, Poland

**Keywords:** brassinosteroid signaling, bioinformatics tools, gene expression, gene promoters, *in silico* analysis, *Oryza sativa*

## Abstract

Brassinosteroids (BRs) regulate a diverse spectrum of processes during plant growth and development and modulate plant physiology in response to environmental fluctuations and stress factors. Thus, the BR signaling regulators have the potential to be targeted for gene editing to optimize the architecture of plants and make them more resilient to environmental stress. Our understanding of the BR signaling mechanism in monocot crop species is limited compared to our knowledge of this process accumulated in the model dicot species - *Arabidopsis thaliana*. A deeper understanding of the BR signaling and response during plant growth and adaptation to continually changing environmental conditions will provide insight into mechanisms that govern the coordinated expression of the BR signaling genes in rice (*Oryza sativa*) which is a model for cereal crops. Therefore, in this study a comprehensive and detailed *in silico* analysis of promoter sequences of rice BR signaling genes was performed. Moreover, expression profiles of these genes during various developmental stages and reactions to several stress conditions were analyzed. Additionally, a model of interactions between the encoded proteins was also established. The obtained results revealed that promoters of the 39 BR signaling genes are involved in various regulatory mechanisms and interdependent processes that influence growth, development, and stress response in rice. Different transcription factor-binding sites and cis-regulatory elements in the gene promoters were identified which are involved in regulation of the genes’ expression during plant development and reactions to stress conditions. The *in-silico* analysis of BR signaling genes in *O. sativa* provides information about mechanisms which regulate the coordinated expression of these genes during rice development and in response to other phytohormones and environmental factors. Since rice is both an important crop and the model species for other cereals, this information may be important for understanding the regulatory mechanisms that modulate the BR signaling in monocot species. It can also provide new ways for the plant genetic engineering technology by providing novel potential targets, either cis-elements or transcriptional factors, to create elite genotypes with desirable traits.

## Introduction

Brassinosteroids (BRs) are a class of steroidal plant hormones that control many physiological and developmental processes, such as seed development, germination, plant growth in the dark (skotomorphogenesis), transition to photomorphogenesis, cell division and elongation, differentiation of the tracheary system, reproduction, as well as plant yield (Wei and Li, 2016; Gao et al., 2019; Gruszka, 2020). It is also known that BR influences plant response to environmental (biotic and abiotic) stresses (Bajguz et al., 2020; Liu et al., 2021). Over the last 3 decades, studies have identified and characterized various components of the BR signaling in the dicot model species *Arabidopsis thaliana*, which is now one of the best described molecular signaling pathways in plants (Gruszka, 2020). Furthermore, the BR signaling components are potential targets for rational molecular design to boost plant growth and response to environmental stresses (Liu et al., 2021).

In contrast, our understanding of the BR signaling mechanism in monocot crop species is limited compared to our knowledge of this process in *Arabidopsis*. Therefore, identifying new BR signaling components in monocots, including cereals, is an ongoing process and it has already resulted in identifying some monocot-specific components of the BR signaling (Gruszka, 2020). Among the monocots, the BR signaling mechanism has been described to the most significant degree in rice (*Oryza sativa*) and serves as a model and reference for other monocots (Gruszka, 2018; Liu et al., 2021). Rice is one of the staple foods for 50% of the world population (Hussain et al., 2022). Several studies have been conducted to identify the BR signaling components in rice and other monocot species, based on the rice genome sequence information available in the public domains. However, the list of identified and functionally characterized BR signaling genes is still limited, particularly in other monocot crop species (Liu et al., 2021).

Forward and reverse genetics approaches were used to characterize the components of the BR signaling (Vriet et al., 2012; Zhang et al., 2014; Gao et al., 2021). The studies conducted in rice allowed for the identification of key players in the BR signaling pathway, including Brassinosteroids-Insensitive 1 (*OsBRI1*), and its coreceptor BRI1-Associated Receptor Kinase1 (*OsBAK1*), Glycogen Synthase Kinase 1 and 2 (*OsGSK1/2*) and Brassinazole-Resistant 1 (*OsBZR1*) transcription factor. The results confirmed that the BR signaling is conserved between monocotyledonous and dicotyledonous plants (Tanaka et al., 2009; Tong and Chu, 2018). It is interesting to note that several BR signaling components in rice, such as Leaf and Tiller Angle Increased Controller (*OsLIC*), DWARF and Low-Tillering (*OsDLT*)*,* Enhanced Leaf inclination and Tiller number1 (*ELT1*)), Taihu Dwarf1 (*OsTUD1*), Erect Leaf1 (*ELF1*), *OsRAVL1*, GrainWidth5 (*OsGW5*)*,* and *OsPRA2*, do not have orthologs in *Arabidopsis* what suggests that in monocots the BR signaling components may have specific functions or that there might be some redundancy between them (Liu et al., 2017; Tong and Chu, 2018). In rice, another component of BR signaling the ABI3/VP1 RAV-Like1 (*RAVL1*) gene enhances the expression of *BRI1* and many BR biosynthesis genes (Je et al., 2010) which is rather unique among regulatory mechanisms of the BR-dependent gene expression. On the other hand, in *Arabidopsis* there are also components of the BR signaling that have not been identified in rice, such as the Protein Phosphatase 2A (*PP2A*) and BRI1-Supressor1 (*BSU*) phosphatases (Liu et al., 2017; Tong and Chu, 2018). To date, 39 BR signaling genes have been identified and characterized in rice by different researchers and using various approaches. The genes and their functions were described in a review by Gruszka (2020) and a list of these genes is given in (Supplementary Table S1).

The identification of monocot-specific components of the BR signaling is a very important issue, taking into account that cereal crop mutants with defects in the BR signaling may be applied in breeding programs due to their favorable traits, such as erect stature which allows dense planting or enhanced tolerance to stress conditions (such as drought). Importantly, several studies have been published regarding mutants deficient in the BR metabolism in the last few years, suggesting that the mutants defective in the BR biosynthesis or signaling are more tolerant to drought (Feng et al., 2015; Gruszka et al., 2016; Gruszka, 2020). The *OsBRI1* gene, the first of the BR signaling components, was shown to encode a functional BR receptor using forward genetics in rice, which revealed typical BR-defective phenotypes, such as erect leaves and dwarfism in plants carrying mutations of this gene (Nakamura et al., 2006). Loss-of-function mutants of the second BR signaling gene—*OsBAK1,* which encodes a component of the BR receptor complex, also show the phenotype of erect leaves and BR insensitivity (Li et al., 2009). However, the loss-of-function mutations in the *OsGSK1/2* genes of rice, which encode homologs of the major negative regulator of the BR signaling in *Arabidopsis*—BIN2, enhance BR sensitivity in lamina inclination test and plant height, indicating the conserved functional role of the GSK proteins in the BR signaling in rice (Gao et al., 2019). In addition, *TaGSK* genes with point mutations display hypermorphic effects in wheat (*Triticum aestivum*)*,* which may be an effective strategy to overcome redundancy among the GSK genes in manipulating the BR signaling in wheat (Hou et al., 2019). Recently, mutations of the newly identified gene *OsBHS1* have been generated by the CRISPR/Cas 9 approach (Zhang et al., 2022). These mutants exhibited BR hypersensitivity regarding the bending angle of the lamina joint*.* The BR signaling components have been functionally characterized mainly in rice, which helps modern agriculture in order to get desired phenotypes like semi-dwarfism, stress tolerance, high yields, and erect leaves in other cereals. Plant hormone research focuses on these traits in cereals, because they serve as potential targets for improving crop yields (Hwang et al., 2021; Manghwar et al., 2022). Insight into the mechanisms regulating the coordinated expression of the BR signaling genes in rice during plant development and reaction to environmental stresses will allow for a better understanding of the BR signaling and response during plant development and adaptations to constantly changing environmental conditions.

Therefore, this study was aimed at conducting *in silico* analysis of promoter sequences of the rice genes encoding components of the BR signaling, expression profiles of these genes in various tissues, at different developmental stages, and in reaction to various environmental stresses, as well as predicting interactions between the encoded proteins. Recently, *in silico* analysis of 40 different Germin-like proteins (*OsGLP*) gene promoters was conducted in rice. The study indicated that genes which contained promoters belonging to the same clade displayed a similar pattern of gene expression across various transcription factor binding sites (TFbs). As a result of the evolution, the promoter regions of the *OsGLP* genes have become neofunctionalized to cope with various biotic and abiotic stresses (Das et al., 2019 **)**. Similarly, *in silico* approaches were followed to study the presence of cis-elements in promoters of the Pathogenesis related (*PR*) genes (Kaur et al., 2017). It was reported that cis-elements could be utilized to manipulate expression patterns in the desired manner, which further opens up the possibility of plant genetic engineering to protect crops from environmental challenges. Additionally, the natural resistance-associated macrophage protein (OsNRAMP) family of transporter proteins was characterized using *in silico* methods and tools (Mani and Sankaranarayanan, 2018). In addition, the *osr40c1* promoter region was used to discover multiple stress-responsive cis-acting regulatory elements in the *indica* rice variety “Pokkali”, which were shown to be induced by both drought and abscisic acid (ABA). Therefore, the isolated promoter sequences could be employed in rice genetic transformation to regulate the expression of abiotic stress-induced genes (de Silva et al., 2017).

**FIGURE 1:**
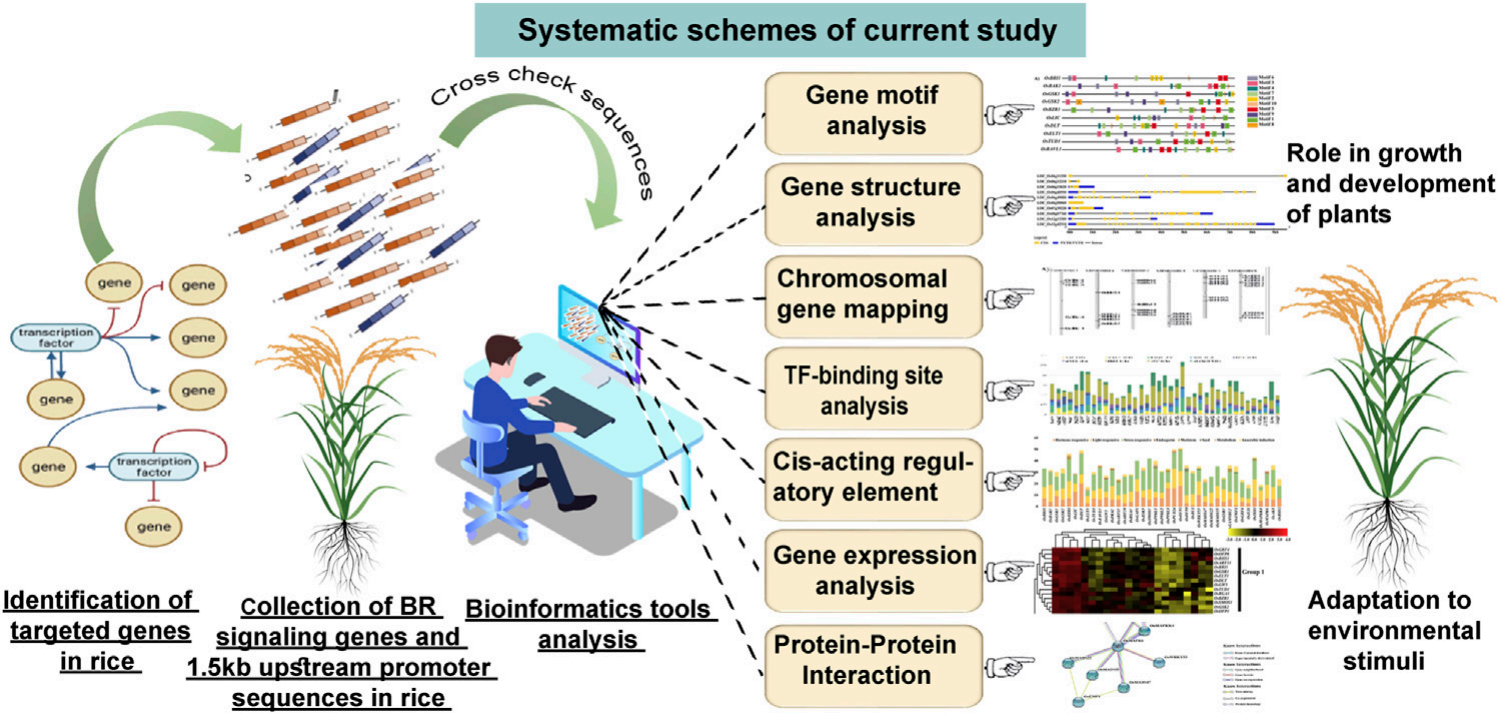
Systematic representation of current study.

The above reports indicate that the *in silico* analyses of promoter sequences of genes involved in various biological processes in plants may provide valuable information about mechanisms which regulate their expression and coordinated action during plant development and in response to environmental cues. Therefore, we decided to perform this kind of *in silico* analysis of promoter sequences, gene expression profiles, and prediction of protein interaction model for the 39 BR signaling components in rice, in order to get insights into developmental and environmental factors and mechanisms which regulate their coordinated action during plant development and adaptation to environmental conditions (Figure 1). This is particularly important taking into account the importance of rice as a crop and model species (for other monocots, including cereals) and the potential application of semi-dwarf mutants of cereals defective in the BR signaling in breeding programs, based on their erect growth habit and enhanced drought tolerance. Finally, this kind of analysis may also allow a deeper insight into the role of BR in the regulation of important aspects of plant biology.

## Materials and Methods

### Retrieval of the BR Signaling Genes and Their Structure Analysis

This study was performed on 39 promoter sequences and genes which have been identified through functional characterization as being involved in the BR signaling processes in rice based on published literature data mining (Supplementary Table S1). Full-length sequences of these genes, as well as their coding sequences (CDS), and sequences of encoded proteins, were retrieved from Rice Genome Annotation Project (RGAP) (http://rice.uga.edu/cgi-bin/gbrowse/rice/). All gene sequences were verified using Phytozome v 13. (https://phytozome-next.jgi.doe.gov/) and cross-checked with the UniProtKB database (https://www.uniprot.org/uniprot/Q7XI96). Gene Structure Display Server 2 (GSDS2) (http://gsds.cbi.pku.edu.cn) was used to illustrate the exon/intron structure of the rice BR signaling genes.

### Chromosomal Localization of Genes and Phylogenetic Analysis Using 1.5 kb Upstream Promoter Sequences

We retrieved 1.5 kb upstream regions encompassing promoter sequences of the 39 BR signaling genes from Phytozome v 13 and verified them against NCBI (Supplementary file S1). A chromosomal map of the 39 BR signaling genes was built with the Rice Database Oryzabase-Shigen (https://shigen.nig.ac.jp/rice/oryzabase/) using the Chromosome Map Tool (http://viewer.shigen.info/oryzavw/maptool/MapTool.do). In order to conduct the phylogenetic analysis, the 39 promoter sequences (1.5 kb upstream regions) of the analyzed BR signaling genes were analyzed by the neighbor-joining method using the p-distance model in the MEGA 7 software (Kumar et al., 2016). The percentage of replicate trees in which the associated taxa clustered together in the bootstrap test (1000 replicates) was calculated. We provided the unique identification (unique ID) for each BR signaling gene to perform and interpret these analyses (Table 1).

**TABLE 1:** Information about the genes involved in the BR signaling in rice and their encoded proteins.

Gene ID	Gene name	Nomenclature	Start (bp)	End (bp) Strand	Genomic length (bp)	Transcript length (bp)	Exons: Introns	Protein length (aa)
LOC_Os01g10840	*OsGSK1*	OsBRs1-1	4350502	5778466	4186	1846	12:11	405
LOC_Os01g12690	*OsOFP1*	OSBRs1-2	7009032	7010505	1473	1473	01:00	395
LOC_Os01g12890	*OsEMF1*	OsBRs1-3	7157779	7164385	6606	4300	04:03	1058
LOC_Os01g52050	*OsBRI1*	OsBRs1-4	29927542	29931487	3945	3945	01:00	1122
LOC_Os01g64430	*OsOFP8*	OSBRs1-5	37396144	37397573	1429	1429	01:00	229
LOC_Os02g22130	*OsGAP1*	OsBRs2-1	13173638	13176606	2968	1628	03:02	166
LOC_Os02g47280	*OsGRF4*	OsBRs2-2	28863172	28866997	3825	1721	03:02	423
LOC_Os02g52340	*OsMADS22*	OsBRs2-3	32038901	32045130	6229	1567	08:07	229
LOC_Os02g54600	*OsMAPKK4*	OsBRs2-4	33442068	33443948	1880	1880	01:00	370
LOC_Os02g58390	*OsELT1*	OsBRs2-5	35705634	35710799	5165	2754	02:01	691
LOC_Os03g08754	*OsMADS47*	OsBRs3-1	4519404	4525778	6374	1460	08:07	251
LOC_Os03g13010	*OsTUD1*	OsBRs3-2	7029148	7031702	2554	2554	01:00	460
LOC_Os03g38210	*OsGAMYBL2*	OsBRs3-3	21212848	21215801	2953	1829	03:02	523
LOC_Os03g44500	*OsPPKL1*	OsBRs3-4	25042314	25051072	8758	3743	21:20	1004
LOC_Os03g45420	*OsPUB24*	OsBRs3-5	25638650	25642193	3543	3459	02:01	825
LOC_Os03g49990	*OsRGA1*	OsBRs3-6	28512624	28515179	2555	2555	01:00	626
LOC_Os04g49230	*OsRAVL1*	OsBRs4-1	29366455	29368515	2060	2060	01:00	317
LOC_Os04g54900	*OsILI1*	OsBRs4-2	32657752	32658190	438	338	02:01	105
LOC_Os04g56500	*OsIBH1*	OsBRs4-3	33676316	33683540	7224	6618	01:00	203
LOC_Os04g56850	*OsARF11*	OsBRs4-4	33888003	33894393	6390	3849	14:13	956
LOC_Os04g58750	*OsBSK3*	OsBRs4-5	34942979	34949751	6772	2316	08:07	359
LOC_Os05g05240	*OsPPKL2*	OsBRs5-1	2564220	2573158	8938	3494	21:20	892
LOC_Os05g06280	*OsBHS1*	OsBRs5-2	3203041	3214116	11075	3584	19:18	1100
LOC_Os05g09520	*OsGW5*	OsBRs5-3	5365121	5366701	1580	1494	02:01	498
LOC_Os05g11730	*OsGSK2*	OsBRs5-4	6657480	6661493	4013	1863	12:11	403
LOC_Os05g27730	*OsWRKY53*	OsBRs5-5	16150265	16152747	2482	2075	05:04	488
LOC_Os05g32270	*OsRLA1*	OsBRs5-6	18812389	18817699	5310	1484	07:06	315
LOC_Os06g03710	*OsDLT*	OsBRs6-1	1465498	1468600	3102	3102	01:00	618
LOC_Os06g06090	*OsMAPK6*	OsBRs6-2	2806542	2813004	6462	1815	06:05	399
LOC_Os06g11330	*OsMADS55*	OsBRs6-3	5953592	5963184	9592	600	06:05	200
LOC_Os06g12210	*OsBU1*	OsBRs6-4	6556801	6557319	518	264	02:01	88
LOC_Os06g15620	*OsGSR1*	OsBRs6-5	8847701	8848847	1146	1042	02:01	111
LOC_Os06g48950	*OsARF19*	OsBRs6-6	29656968	29665195	8227	3858	14:14	1139
LOC_Os06g49080	*OsLIC*	OsBRs6-7	29738879	29742503	3624	1834	10:09	391
LOC_Os06g50060	*OsPRA2*	OsBRs6-8	30336258	30336936	678	678	01:00	226
LOC_Os07g39220	*OsBZR1*	OsBRs7-1	23483808	23485344	1536	1407	02:01	299
LOC_Os08g07760	*OsBAK1*	OsBRs8-1	4344170	4350502	6332	2710	11:10	625
LOC_Os12g13380	*OsAK3*	OsBRs12-1	7476415	7480311	3896	1173	06:05	242
LOC_Os12g42310	*OsPPKL3*	OsBRs12-2	26306762	26315803	9041	4179	21:20	1010

### Analysis of Transcription Factor Binding Sites (TFbs), the CpG/CpNpG Islands, and Tandem Repeats Within the 1.5 kb Upstream Promoter Sequences

Transcription factor binding sites (TFbs) were identified within the promoter regions to elucidate their interactions with various groups of transcription factors during the regulation of expression of the BR signaling genes. The PlantPAN 3.0 software (http://plantpan.itps.ncku.edu.tw/promoter.php) was used to identify TFbs in the promoter regions of the 39 BR signaling genes. The Multiple promoter analysis program (http://plantpan2.itps.ncku.edu.tw/gene_group.php?#multipromoters) was used to identify the common TFbs in different promoter regions. Furthermore, the PlantPAN 3.0 was also utilized for the analysis of CpG/CpNpG islands and tandem repeats (TRs) within the 39 (1.5 kb upstream) promoter sequences (Chow et al., 2016). CpG islands based on their DNA sequence must fulfil three conditions 1) GC content above 50%, 2) ratio of observed-to-expected number of CpG dinucleotides above 0.6 and 3) length greater than 200 bp.

### Identification of Conserved Motifs and Cis-regulatory Elements in the 1.5 kb Upstream Promoter Sequences

The 1.5 kb upstream promoter regions of the 39 BR signaling genes in *O*. *sativa* were selected to identify the key cis-regulatory elements. The PlantCARE database of plant cis-acting regulatory elements (http://bioinformatics.psb.ugent.be/webtools/plantcare/html/) was used to detect cis-regulatory elements in these sequences (Lescot et al., 2002). Moreover, the MEME tool (http://meme-suite.org/index.html) (Bailey et al., 2009) was used to determine the conserved motifs, which were visualized using the TBtools v0.6655 (Chen et al., 2020). We set the number of motif sites at 10 per sequence and the number of motifs at zero or one for each sequence.

### Analysis of Expression Profiles of the BRs Signaling Genes

In order to characterize expression patterns of the BR signaling genes in rice, the following tissues and developmental stages were analyzed: seed (72 h after imbibition), embryo (after germination) and endosperm (7, 14, and 21 days after pollination), root and shoot (stage with 2 tillers), radicle (after emergence in the dark and light), stem (heading stage, 5 days before heading), leaf and flag leaf (4–5 cm young panicle, 5 days before heading, 14 days after heading, and young panicle at stage 3), sheath (young panicle at stage 3), spikelet (3 and 5 days after pollination), panicle (4–5 cm young panicle, young panicle at stage 3, 4, and 5, and panicle at heading stage). The expression profiling data were extracted from the CREP database (http://crep.ncpgr.cn/) (Wang et al., 2010). Furthermore, the expression profiles of the BR signaling genes in response to different abiotic stresses (cold, heat, drought, and salt stress) were also analyzed. The expression profiling data related with responses to the abiotic stresses were extracted from the Plant Public RNA-seq Database (http://ipf.sustech.edu.cn/pub/plantrna/). The expression profiles of the BR signaling genes under different abiotic stresses were used to generate a hierarchical cluster analysis of the complete method with log2 value. The heatmap was prepared using the TBtools (Toolbox for biologists) v0.6655 with red/black and green color scheme markers.

### Analysis of Interactions Among Proteins Encoded by the BR Signaling Genes of Rice

Protein-protein interactions were predicted using the STRING v11.0 (https://string-db.org/) tool accessed on 20 February 2022 with a high confidence score of 0.7 (Szklarczyk et al., 2015). The functional enrichment analysis of the interactome was done through the level of 0.01.

## Results

### Analysis of the BR Signaling Genes in Rice and Characterization of Their Structures

Using the above-mentioned, various computational resources and previously published literature, we have verified genome annotation of 39 genes of rice which have been so far functionally characterized as participating in the BRs signaling in this species (Supplementary Table S1) and retrieved their sequences. The genomic information such as chromosomes in which the genes are localized, full length of the gene in the rice genome, number of introns and exons in each gene, and length of encoded proteins have been described in detail in Table 1.

The position and distribution of exons and introns are fundamental characteristics in gene structure analysis. The ratio of intron:exon architecture of the 39 BR signaling genes was checked graphically to compare the impact of evolution on the conversion of size and number of introns and exons within the BRs genes. Our results suggested that the numbers of exons: introns varied from 21:20 to 01:00. Furthermore, the gene size of 39 BRs genes ranged from the longest 11075 bp (*LOC_Os06g11330*, *OsMADS55*) to the lowest 438 bp (*LOC_Os04g54900*, *OsILI1*) (Table 1, Supplementary Figure S1).

### Chromosomal Localization and Phylogenetic Analysis of Promoter Sequences of the 39 BR Signaling-Related Genes

**FIGURE 2:**
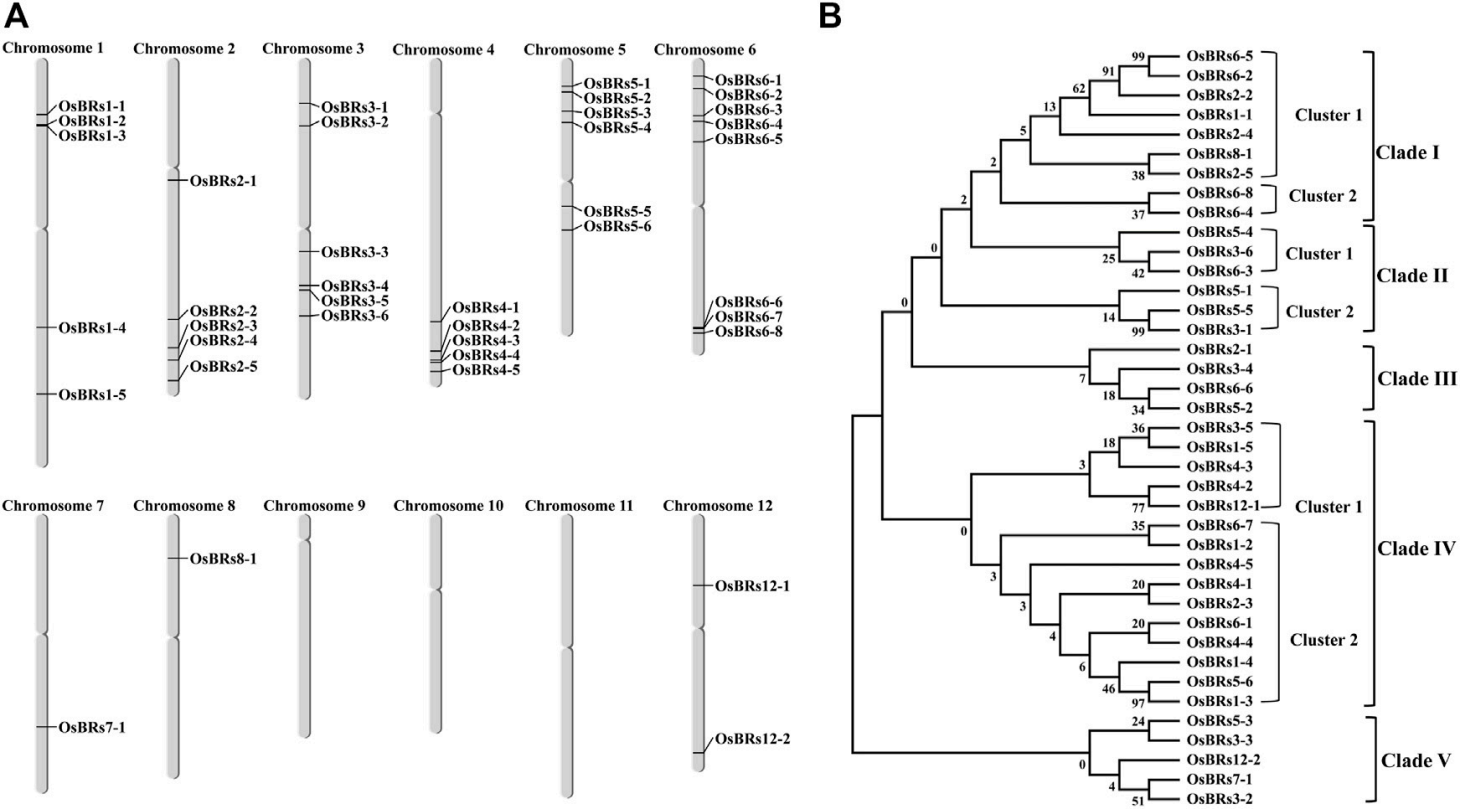
**(A)** Graphical representation of the chromosomal distribution of the 39 promoter sequences of the BR signaling genes in rice. **(B)** Phylogenetic relationship of the 1.5 kb upstream promoter sequences of the 39 BR genes. The sequences were aligned using ClustalOmega, and the tree was constructed by MEGA 7 using the neighbor-joining method.

The chromosome mapping of the 39 BR signaling gene promoters indicated that they are unevenly distributed on 9 out of 12 chromosomes (Figure 2A). Based on the phylogenetic analysis of the 1.5 kb-long upstream sequences of the 39 BR signaling genes, they were divided into 5 different clades using the bootstrap value from 0 to 1000. Out of the 5 clades, clade I was divided into two clusters in which cluster 1 contained 7 gene promoters and cluster 2 had 2 promoter sequences (Figure 2B). Clade II comprised of two clusters in which cluster one contained 3 gene promoters (*OsBRs5-4*, *OsBRs3-6*, and *OsBRs6-3*) and cluster 2 also consisted of 3 gene promoters (*OsBRs5-1, OsBRs5-5*, and *OsBRs3-4*). Clade III was the smallest clade consisting of 4 gene promoters (*OsBRs2-1*, *OsBR3-4*, *OsBRs5-2* and *OsBRs6-6*). Clade IV was found to be the largest clade with two clusters. There were 5 gene promoters in cluster 1, while cluster 2 comprised 10 gene promoters. Clade V was second the smallest clade, after clade III with 5 genes from chromosomes 3, 5, 7 and 12. The phylogenetic analysis of the 39 BR signaling-related gene promoters revealed that the promoters on chromosome 6 named *OsBRs6-5* and *OsBRs6-2* have high homology, up to 99 percent with each other (Figure 2B).

### Identification of Common TFbs in Promoters of the BR Signaling Genes

The promoter sequences of 39 BR signaling genes were used to find common TFbs using PlantPAN 3.0. The database released 26,077 TFbs collected from input queries (Supplementary Table S2). We identified different TFs involved in regulation of growth and development stages, as well as reaction to biotic and abiotic stresses, with the link to the BR signaling (TCP, WRKY, bHLH, NAC, BES1, bZIP, MYB, GATA and AP2/ERF). From 26,077 TFbs, we have collected common TFbs present in all targeted gene promoters (Supplementary Table S3). The results suggest that the most common (present in all input query genes) and the most represented of TFbs was the TCP-binding site (4620 TFbs in total). In the 39 BRs signaling gene promoters, the highest number of TCP TFbs was found in *OsMADS22,* followed by the *OsSMOS1* with 227 and 222, respectively. The second most common TFbs was AP2/ERF-binding site (1756) and the highest numbers of these TFbs were present in *OsLIC* (197) and *OsAK3* (194). The other three TFbs, bHLH-binding site, bZIP-binding site, and MYB-binding site were also common in all 39 gene promoters, 1145, 995, 874, respectively. The highest number of bHLHbs (142) was found in the *OsOFP1* promoter region, bZIPbs (93) in the *OsDLT* promoter*,* while MYBbs (78) in the *OsGSK1* gene promoter. The NACbs (281 in total) were present in most of the gene promoters except three gene promoters (*OsGAMYBL2, OsILI1, OsAK3*)*.* The WRKYbs were found 593 times in total, except eleven gene promoters (*OsBU1*, *OsLIC*, *OsPPKL3, OsMADS47, OsMADS22, OsMADS55, OsILI1, OsAK3, OsBHS1, OsELT1* and *OsRGA1*). The WRKYbs were present in the highest number (73) in promoter region of the *OsPPKL2* gene. The GATAbs were found 585 times in total and most frequent in the *OsMADS47* (49) promoter region, whereas they were not present in the *OsOFP1, OsBHS1, OsELT1* and *OsGSK1* gene promoters*.* Surprisingly, among the analyzed TFbs the lowest number (33) was found for the BES1 transcription factor (one of the major regulators of the BR-dependent gene expression) which was present in 17 promoters out of 39 targets, and the highest number (4) of the BES1 TFbs was present in the three (*OsBRI1*, *OsDLT*, *OsTUD1*) gene promoters (Supplementary Table S3). Furthermore, our analysis indicated that promoters belonging to the same class of genes, such as *ARF11* and *ARF19* (which encode auxin-response transcription factors) have a different pattern of TFbs. Similarly, another group of genes belonging to the same family, *OsPPKL3* and *OsPPKL2* (which encode protein phosphatases), also showed significant differences in the pattern of TFbs in their promoters. These results indicate that the differences in the same class of gene promoters may provide a basis for their sub- or neofunctionalization which will be discussed below.

**FIGURE 3:**
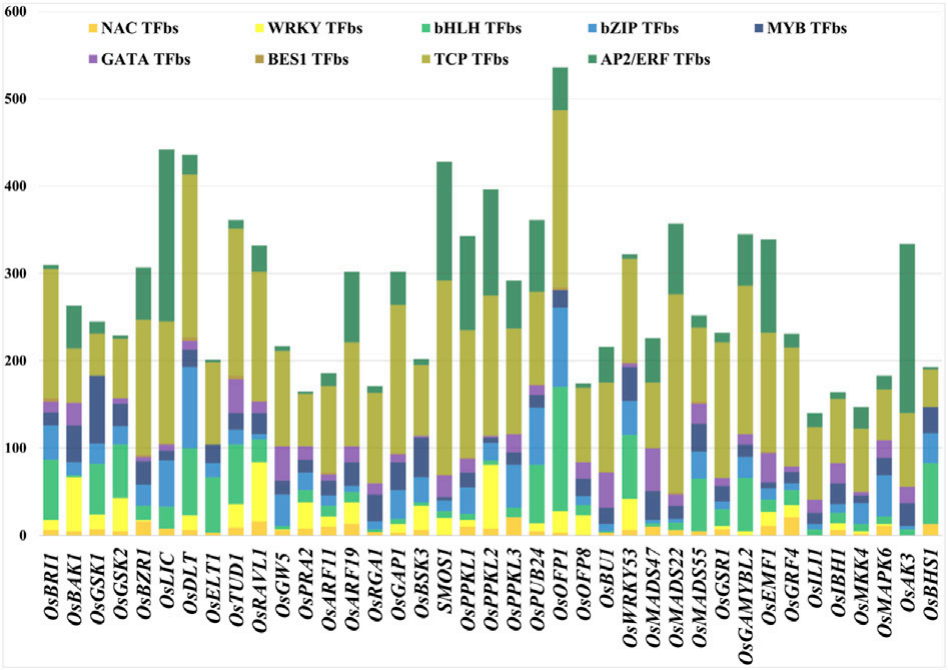
Different numbers of TFbs in each 1.5-kb upstream promoter sequence. Different colors represent different TFbs.

In the current study, among the 9 common TFbs within promoters of the 39 BR signaling-related genes, the highest number of TFbs was present in the *OsOFP1* upstream region*,* while the lowest number of TFbs was found in the *OsILI1* gene promoter (Figure 3, Supplementary Table S3). Our results of the occurrence and frequency of the TFbs suggest that the expression of some of the BR signaling genes (*OsBRI1, OsBZR1, OsDLT, OsTUD1, OsARF11, OsGAP1, OsPPKL1, OsPUB24, OsBU1, OsWRKY53, OsEMF1, OsRAVL1*) is regulated by a particularly diverse group of transcription factor families (their promoter regions contain all 9 common TFbs (Figure 3, Supplementary Table S3). This result indicates that the expression of these genes may be regulated by a particularly broad group of hormonal, developmental and environmental factors. Our analysis of promoters of the BR signaling genes identified several TFs which are involved in various hormonal and physiological regulatory mechanisms. However, further experimentations are still needed to understand the biological importance of the BRs signaling genes with the high and low number of TFbs in their promoters.

### Tandem Repeats and CpG/CpNpG Analysis Within Targeted Gene Promoters

It is important to understand the epigenetic regulatory mechanisms, such as DNA methylation, chromatin rearrangement, or histone modifications which may influence gene expression and consequently modify plant physiology and development. In plants, DNA is methylated in 5′-CG-3′ symmetrical dinucleotides, 5′-CNG-3′ symmetrical trinucleotides (where N denotes any nucleotide), as well as in 5′-CNN-3′ asymmetrical trinucleotides (where N stands for A, T or C). In the present study the 1.5 kb upstream promoter regions of the 39 BR signaling-related genes were analyzed to identify the CpG/CpNpG motifs. In the present study, we have checked each gene promoter and found that CpG/CpNpG islands were present in 30 out of 39 analyzed promoter regions, except for *OsGSK1, OsGSK2, OsELT1, OsGW5, OsPRA2, OsBSK3, OsIBH1, OsMAPKK4,* and *OsAK3*. These genes encode proteins which show different enzymatic activity and participate in various stages of the BR signaling pathway. The highest length of CpG island was observed in the *OsBZR1* gene promoter (1500 bp) showing similarity in length to the targeted query (1500bp) (Table 2). The presence of CpG/CpNpG islands in 30 of the analyzed BR signaling gene promoters may hint toward their transcriptional repression due to methylation influencing the gene expression. However, there are rice BR signaling genes in which the CpG/CpNpG islands were not detected, suggesting that in these promoters, expression of the genes is not repressed due to cytosine methylation and/or gene regulation may occur via a different epigenetic regulation mechanism, such as post-translational histone modifications. The targeted promoters in which methylation levels of the CpG islands were absent need to be investigated in future studies. These promoters could be the potential target of further analysis for functional validation to investigate the relationship between methylation levels of the CpG islands and their gene expression levels (Table 2).

**TABLE 2:** List of promoter regions of the BRs signaling-related genes containing the CpG/CpNpG motifs.

Promoter ID	Gene name	Begin site	End site	Length	G + C frequency	CpG o/e ratio	At Skew	CG Skew	Start-p	Strand	Strand-p
LOC_Os01g52050	*OsBRI1*	51	1524	1450	0.54	1.08	−0.05	−0.14	0.72	−	0.79
LOC_Os08g07760	*OsBAK1*	890	1500	611	0.53	0.98	0.19	−0.09	0.49	−	0.96
LOC_Os07g39220	*OsBZR1*	1	1500	1500	0.54	0.77	0.05	−0.09	0.38	−	0.84
LOC_Os06g49080	*OsLIC*	22	1500	1479	0.54	1.16	−0.01	−0.01	0.8	+	0.5
LOC_Os06g03710	*OsDLT*	1	1153	1153	0.55	1.04	−0.05	−0.04	0.67	+	0.53
LOC_Os03g13010	*OsTUD1*	1	684	684	0.49	0.9	0.03	0.07	0.34	+	0.65
LOC_Os04g49230	*OsRAVL1*	190	1500	1311	0.58	1.01	0.08	−0.16	0.68	−	0.95
LOC_Os04g56850	*OsARF11*	1	1167	1167	0.55	0.82	−0.07	0.06	0.4	+	0.83
LOC_Os06g48950	*OsARF19*	836	1500	665	0.54	0.87	0.02	0.04	0.38	+	0.57
LOC_Os03g49990	*OsRGA1*	810	1500	691	0.51	0.78	−0.04	−0.02	0.25	+	0.53
LOC_Os02g22130	OsGAP1	233	1500	1268	0.51	0.93	0.03	−0.01	0.51	−	0.61
LOC_Os05g32270	OsRLA1	1	1167	1167	0.6	0.87	−0.07	−0.07	0.52	−	0.55
LOC_Os03g44500	*OsPPKL1*	630	1500	871	0.61	1.05	0.05	−0.22	0.68	-	0.97
LOC_Os05g05240	*OsPPKL2*	619	1500	882	0.56	0.77	−0.04	−0.16	0.31	−	0.85
LOC_Os12g42310	*OsPPKL3*	760	1500	741	0.55	0.91	0.09	0.01	0.44	−	0.7
LOC_Os03g45420	*OsPUB24*	147	1500	1354	0.5	0.79	−0.05	0.1	0.34	+	0.88
LOC_Os01g12690	*OsOFP1*	1	815	815	0.54	0.98	−0.07	0.04	0.53	+	0.79
LOC_Os01g64430	*OsOFP8*	913	1488	576	0.5	0.52	−0.13	−0.32	0.08	−	0.95
LOC_Os06g12210	*OsBU1*	971	1500	530	0.52	0.83	0.07	−0.2	0.3	−	0.97
LOC_Os05g27730	*OsWRKY53*	722	1500	779	0.54	1.23	−0	−0.36	0.79	−	0.99
LOC_Os03g08754	*OsMADS47*	959	1500	542	0.5	0.88	−0.29	−0.09	0.34	+	0.88
LOC_Os02g52340	*OsMADS22*	56	1500	1445	0.6	0.95	−0.09	−0.19	0.64	−	0.83
LOC_Os06g11330	*OsMADS55*	789	1500	712	0.54	1.03	−0.27	−0.09	0.57	+	0.87
LOC_Os06g15620	*OsGSR1*	865	1500	636	0.51	0.8	0.09	−0.23	0.28	−	0.98
LOC_Os03g38210	*OsGAMYBL2*	1	1114	1114	0.52	0.67	−0.16	−0.14	0.22	−	0.54
LOC_Os01g12890	*OsEMF1*	1	1126	1126	0.58	1.09	−0.22	−0.29	0.73	−	0.82
LOC_Os02g47280	*OsGRF4*	714	1500	787	0.55	1.1	0.2	−0.02	0.68	−	0.92
LOC_Os04g54900	*OsILI1*	814	1500	687	0.49	0.71	0.09	−0.2	0.19	−	0.98
LOC_Os06g06090	*OsMAPK6*	1001	1500	500	0.5	0.91	0.18	−0.29	0.36	−	1
LOC_Os05g06280	*OsBHS1*	853	1500	648	0.58	1	0.19	−0.13	0.57	−	0.98

Tandem repeats (TRs), also called satellite DNA, are repeated sequences adjacent to one another in a head-to-tail pattern at a higher frequency than the surrounding genome region. A DNA sequence containing tandem repeats indicates a greater propensity for mutation, and genes containing tandem repeats in their promoters exhibit higher rates of transcriptional divergence (Vinces et al., 2009). We used 1.5 kb upstream promoter sequence of 39 BR signaling genes to identify the presence of TRs. TRs may be categorized into three groups based on the length of each repeat. The repeat unit less than 6 nucleotides in length is called microsatellite as group one. The second group is minisatellites with 6–100 bp-long repeat units. The third group is megasatellites having longer units, with more than 135 nucleotides. In the present study, promoters of 8 genes (*OsDLT, OsELT1, OsARF11, OsPPKL3, OsOFP8, OsEMF1, OsGRF4* and *OsBHS1*) were found to contain repeats of less than 9 nucleotides in length, falling into the microsatellites group. In promoters of 8 genes (*OsBAK1, OsRLA1, OsPPKL1, OsPUB24, OsBU1, OsMADS22, OsMAPK6* and *OsMAPKK4*) minisatellite repeats were identified. The promoter sequence of the *OsGSK1* gene is the only which contains repeats of the unit which is longer than 100 nucleotides called as megasatellites (Table 3). The results indicated that TRs were present in 17 gene promoters out of 39, suggesting that they have an increased potential for accumulation of mutations during replication (so called polymerase slippage). The presence of TRs in these promoters can be used for mutational study, and it might also participate in gene expression regulation.

**TABLE 3:** List of promoter regions of the BRs signaling-related genes containing tandem repeats.

Promoters ID	Gene name	Start	End	Period size	Copy number	% Matches	% Indels	Score	Entropy (0–2)	Tandem repeat
LOC_Os08g07760	*OsBAK1*	435	498	30	2.1	100	0	128	1.62	AAT​ATA​TCT​TTG​ACT​GTA​TTT​TTC​TAT​TGT
LOC_Os01g10840	*OsGSK1*	158	1088	174	5.3	78	7	898	1.85	Large no of DNA stretches (173bp)
LOC_Os06g03710	*OsDLT*	1419	1455	3	12.3	94	0	65	1.07	GAG
LOC_Os02g58390	*OsELT1*	1305	1336	2	16	93	0	55	1.17	AG
LOC_Os04g56850	*OsARF11*	351	375	3	8.3	100	0	50	1.58	GCA
LOC_Os05g32270	*OsRLA1*	604	643	21	1.9	89	0	62	1.11	GAG​GCG​GTG​GGA​GCG​GGG​GGA
LOC_Os03g44500	*OsPPKL1*	660	732	38	1.9	85	0	101	1.92	ACT​AAA​CAC​AGA​AAG​GGA​TCA​TCC​AAC​GTG​AAC​TTC​TC
LOC_Os12g42310	*OsPPKL3*	510	535	2	13	100	0	52	1	AT
LOC_Os03g45420	*OsPUB24*	119	182	32	2	100	0	128	1.92	GCT​AAC​TAA​AAG​AGC​TAT​AGC​TCA​TGA​TGA​TT
LOC_Os01g64430	*OsOFP8*	350	385	2	18	100	0	72	1	TC
LOC_Os06g12210	*OsBU1*	663	711	24	2	100	0	98	1.68	AAA​ACA​TAG​AAC​TTA​AAT​ATT​TGC
LOC_Os02g52340	*OsMADS22*	1268	1301	10	3.4	91	0	50	1.52	CCTCGCCTCA
LOC_Os01g12890	*OsEMF1*	964	992	2	14.5	100	0	58	1	TC
LOC_Os02g47280	*OsGRF4*	1137	1165	2	15	92	7	51	1	GA
LOC_Os02g54600	*OsMAPKK4*	365	559	76	2.7	79	12	173	1.6	Large no of DNA stretches (69bp)
LOC_Os06g06090	*OsMAPK6*	614	652	11	3.5	82	6	51	0.83	TTTTCTTTTTT
LOC_Os05g06280	*OsBHS1*	358	417	2	29.5	96	3	11	1.11	AT

### Common Cis-Regulatory Elements and Conserved Motifs

**FIGURE 4:**
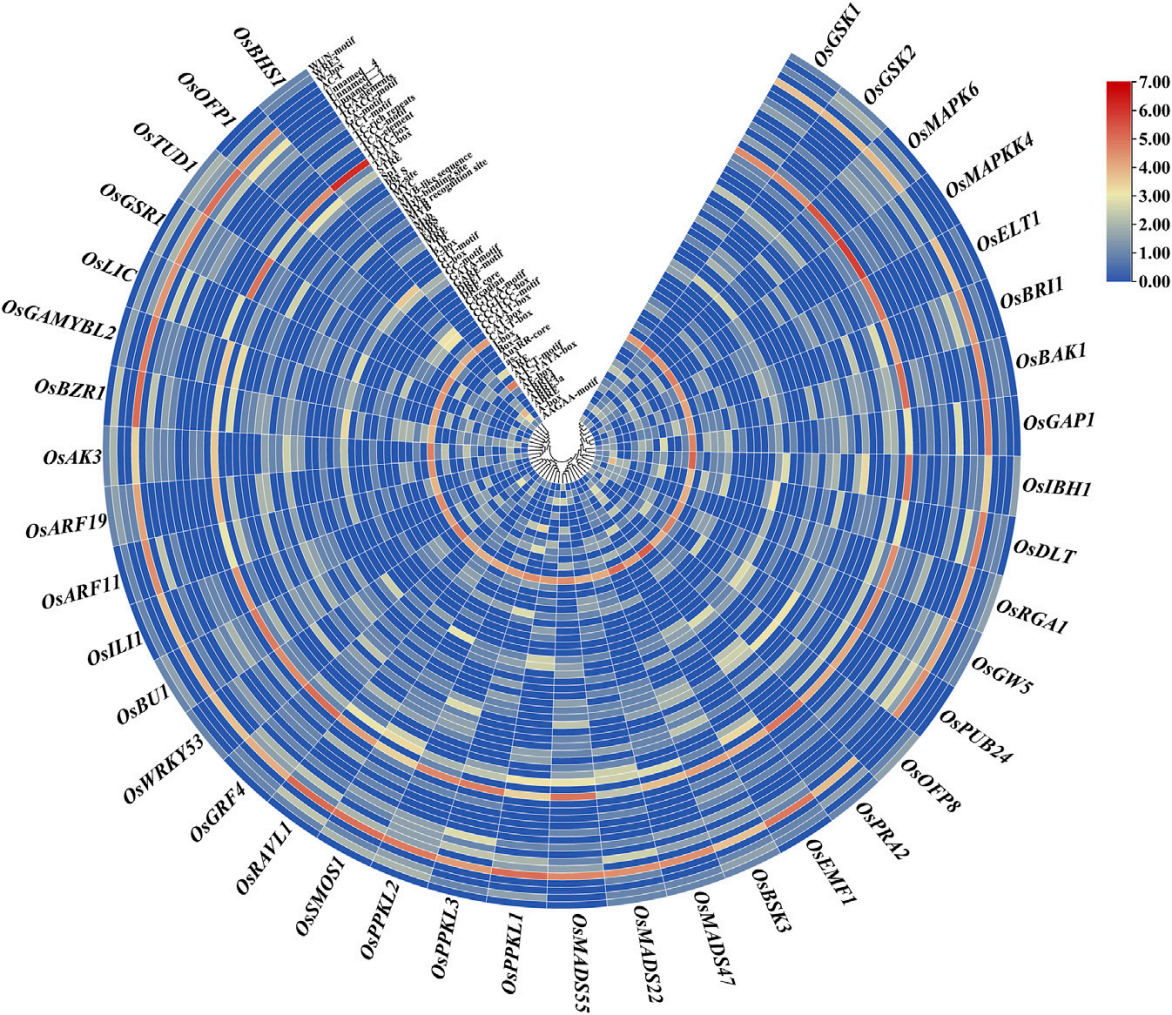
A graphical representation of commonly present cis-regulatory elements in 1.5-kb upstream promoter sequences of the 39 BR signaling genes in *O. sativa.* Abbreviations/Functions: ABRE: ABA-responsive element; ERE: Ethylene-responsive element; ARE: Anaerobic response element; LTR: Low temperature-responsive element; STRE: stress-responsive element; MRE: Myb-related element: DRE: dehydration-responsive element; GARE: thymine- and cytosine-rich repeats.

We identified trans-acting regulatory elements in the 1.5-kb upstream promoter sequences of the 39 BR signaling genes. The obtained result revealed that 97 cis-regulatory elements were identified in all targeted gene promoters. Out of 97 in total, we have identified 59 cis-elements that were present at least in 10% of the analyzed gene promoters (Supplementary Table S4, Supplementary Table S5). Out of the 59 cis-elements, few were present in more than 70% of the analyzed promoter regions: CAAT-box (100%), TATA-box (97%), STRE (92%), AT ∼ TATA-box (87%), ABRE (82%), ARE (79%), G-box (79%), MYC (77%), and WRE3 (74%) (Figure 4, Supplementary Table S5). Furthermore, cis-acting elements were involved in different categories, such as hormones, light, stress responsiveness, growth development stages (seed, endosperm, meristem, metabolism) and anaerobic induction.

**FIGURE 5:**
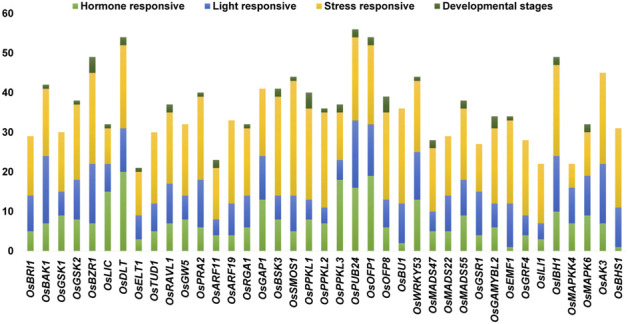
Different categories of cis-regulatory elements according to their biological functions.

The stress-responsive cis-elements were common in all promoters of the BR signaling-related genes. The stress-responsive elements were categorized into biotic and abiotic stress-responsive elements, but not ascribed to any category. The abiotic stress-related cis-elements were represented by ARE, CCAAT-box, DRE core, MBS, MYB recognition site, Myb-binding site, MYB-like sequence, MYB, MYC, STRE, TC-rich repeats, LTR, MBSI, GC-motif, DRE1, and AT-rich element, whereas the biotic stress-related cis-elements included box S, WRE3, W-box, and WUN-motif. In terms of frequency of occurrence in the promoter sequences, the stress-responsive elements in all targeted promoters ranged from the highest in *OsSMOS1* (29) to the lowest in *OsMAPKK4* (6). The second most common category of cis-elements included light-responsive cis-elements (AE-box, G-Box, I-box, Sp1, Box 4, GT1-motif, TCCC-motif, TCT-motif, ATCT-motif, MRE, GA-motif, GATA-motif). These cis elements were identified in 39 promoters of the BR signaling genes. Few other light-responsive cis-elements were also present in several gene promoters, for example AT1-motif was present in promoters of the *OsBRI1* and *OsMAPKK4* genes*,* the ACE motif was present in promoter sequences of the *OsDLT, OsRGA1, OsBU1* and *OsBHS1* genes, the Box II motif was identified in the promoter of the *OsRGA1* gene, the 3-AF1 binding site was found in promoters of the *OsGW5, OsRGA1, OsMADS47,* and *OsGAMYBL2* genes, whereas the Pc-CMA2c motif was present in the promoter of the *OsAK3* gene. Similarly, each promoter of the BR signaling-related genes contained several hormone-responsive cis-elements ranging from the highest number (20) in *OsDLT* to the lowest (1) in the *OsBHS1* and *OsEMF1* genes promoter (Figure 5)*.* Our results revealed that several cis-elements which are intricate in hormonal regulation, such as ABRE (abscisic acid), CGTCA-motif and TGACG-motif (MeJA), TCA-element (salicylic acid), TGA-element and AuxRR-core (auxin), ERE (ethylene), as well as P-box and GARE-motif (gibberellin) were present in promoters of all BR signaling genes (Supplementary Table S4).

We have also identified cis-elements related to developmental stages such as seed, endosperm, meristem, and metabolism. Our results identified that seed, endosperm, meristem, and metabolism were regulated by the RY-element, AACA_motif and GCN4_motif, CAT-box, and O2-site, respectively (Figure 5, Supplementary Table S4). We have observed some cis-elements which were common in our targeted gene promoters, however, with an unknown function, such as ABRE3a, ABRE4, AAGAA-motif, CCGTCC motif and CCGTCC-box, including some still unnamed (Unnamed__1,2,4), but present in more than half of our targeted genes. We can predict that these cis-elements could be potential targets for further investigation within the 39 BR signaling genes (Figure 4, Supplementary Table S4).

**FIGURE 6:**
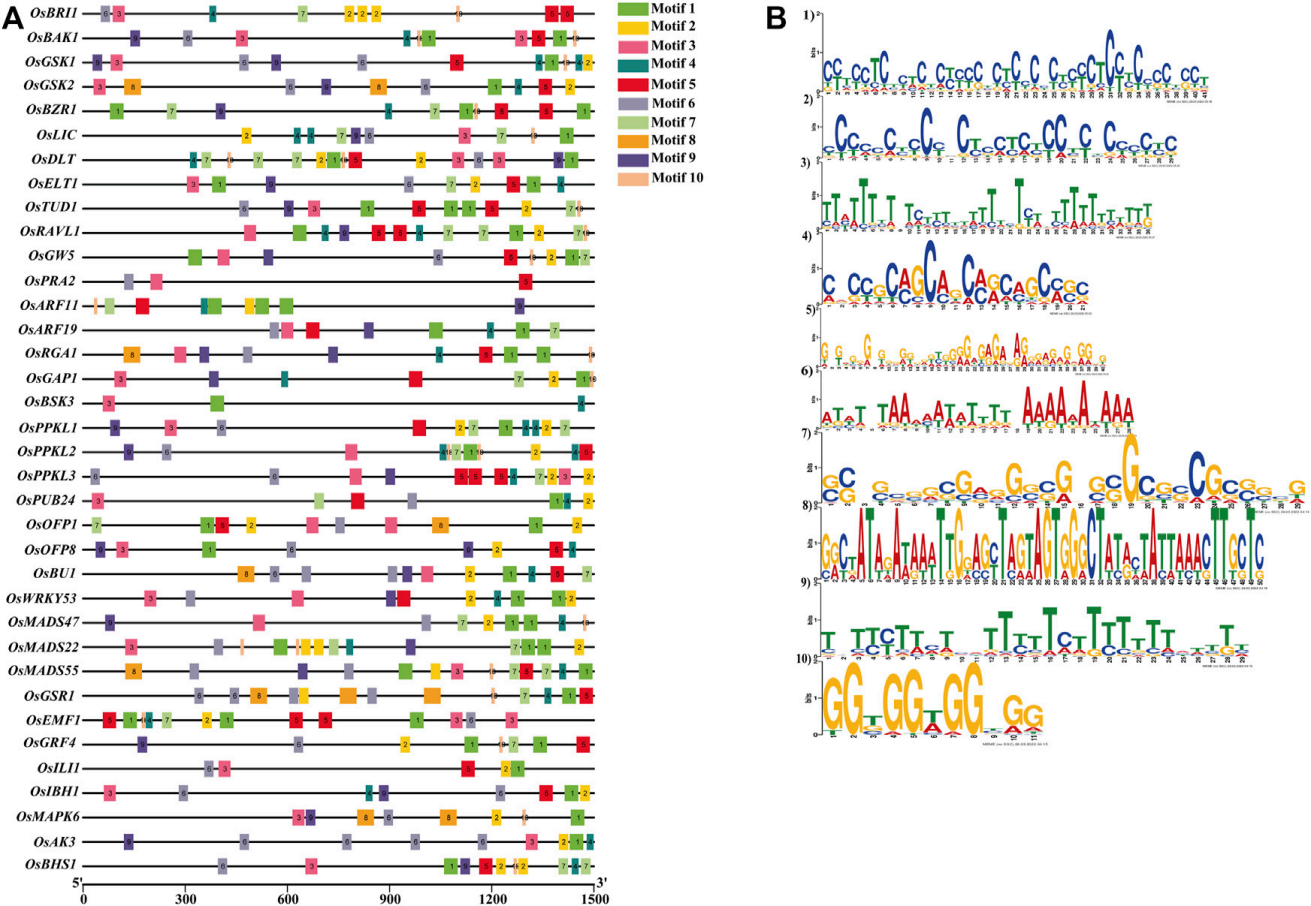
**(A)** Localization of conserved motifs within promoters of the BR signaling genes. **(B)** Logo of the conserved motifs within promoters of the BR signaling genes.

We have also searched conserved motifs within the promoter region of the 39 BRs signaling gene promoters. The results showed that motif 1, motif 3, and motif 9 were highly conserved in all 39 gene promoters. Motif 5 was absent in the *OsBSK3* gene promoter*.* Similarly, motif 2 was absent in promoters of the *OsBSK3* and *OsPRA2* genes, and motif 4 was absent in promoters of the *OsGW5, OsPRA2, OsGRF4* and *OsILI1* genes. In addition, motifs 2, 4, 5, 6, 7, 8, and 10 were present in 94%, 89%, 97%, 84%, 66%, 12%, 89% of the BRs signaling gene promoters, respectively (Figures 6A,B and Supplementary Table S6).

### Expression Pattern of the BR Signaling Genes in Different Tissues in *O.sativa* and During Reaction to Environmental Stresses

We examined the expression patterns of the BR signaling genes at 22 developmental stages using microarrays data from the CREP database. In the expression database, probes were available for 37 out of 39 BR signaling genes. The 37 BRs signaling genes in the array were used for expression profiling, and probes with higher signal values were retrieved.

**FIGURE 7:**
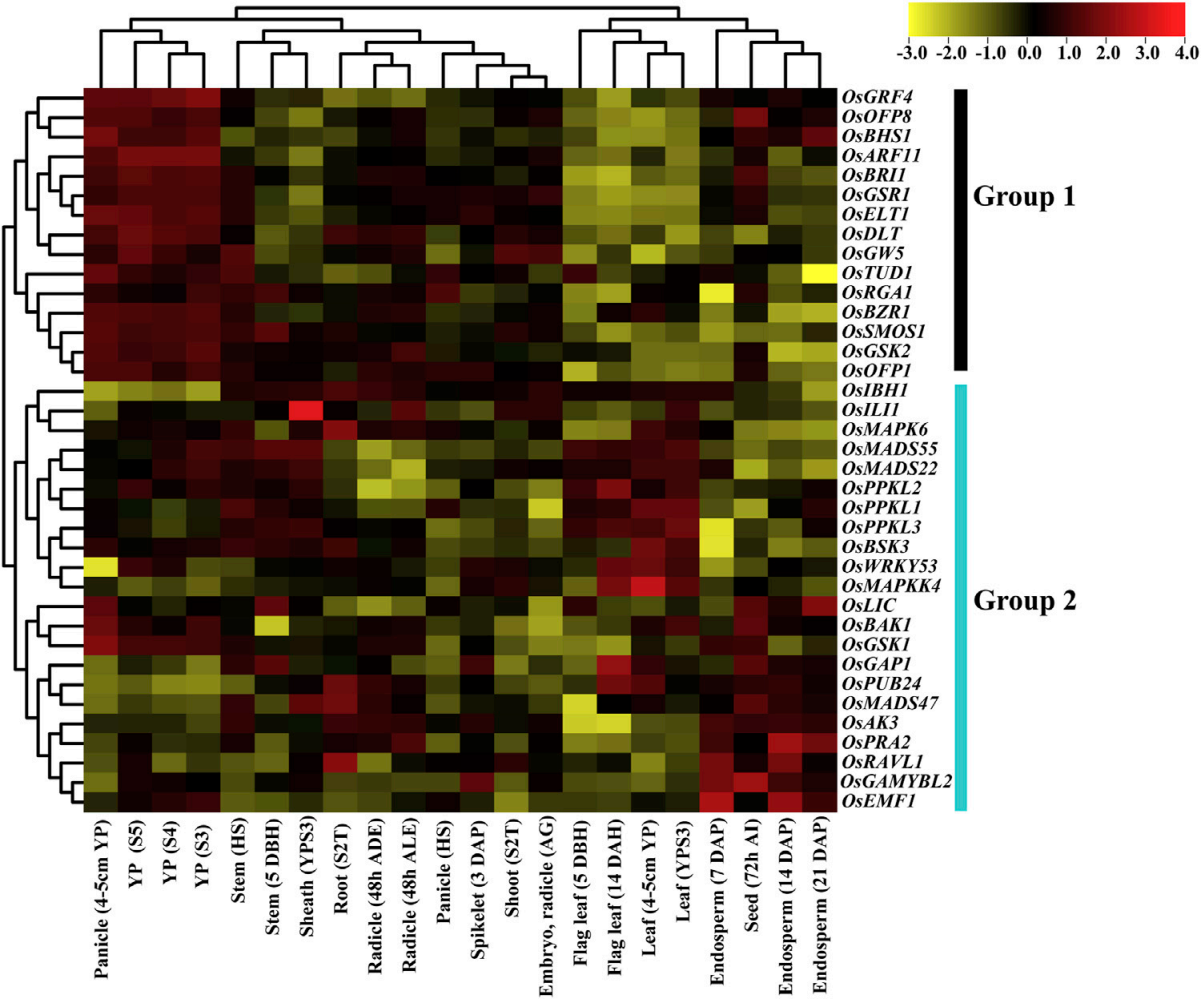
Expression pattern of the BR signaling genes in rice. Abbreviations: YP: Young panicle; HS: Heading stage; DBH: Days before heading; YPS3: Young panicle at stage 3; S2T: Stage with 2 tillers; ADE After dark emergence; ALE: after light emergence; DAP: Days after pollination; DBH: Days before heading; DAH: Days after heading; AI: after imbibition.

Based on hierarchical clustering, expression patterns of 37 BR signaling genes were classified into 2 groups. Group 1 includes 15 genes, and all the genes were differentially expressed at different developmental stages. All genes from group 1 showed the highest expression at different panicle development stages and lowest expression at leaf and flag leaf stages. This trend indicates that genes of group 1 play an essential role in panicle development. In the remaining tissues, such as stem, root, sheath, radicle shoot, seed, and endosperm, the genes of group 1 showed intermediate expression except for *OsOFP8* and *OsBRI1,* which were highly expressed in the seed development stage (Figure 7). In addition, we have found that the *OsRGA1* gene showed intermediate expression at all development stages.

Group 2 comprised 22 genes and their expression patterns indicated that *OsMAPK6, OsMADS55/22, OsPPKL1/2/3, OsBSK3, OsLIC, OsBAK1,* and *OsGSK1* genes were highly expressed in panicle, stem, sheath, and root. Moreover, *OsMADS55/22, OsPPKL1/2/3, OsBSK3, OsWRKY53,* and *OsMAPKK4* genes were highly expressed in leaf and flag leaf*,* while half of the genes (*OsLIC, OsBAK1, OsGSK1, OsGAP1, OsPUB24, OsMADS47, OsAK3, OsPRA2, OsRAVL1, OsGAMYBL2* and *OsEMF1*) showed high expression level in the seed and endosperm tissues. The *OsGAP1*, *OsPUB24, OsMADS47, OsAK3* and *OsPRA2* genes have the highest expression level in stem, sheath, and radicle. Interestingly, the *OsIBH1* gene showed particularly low expression during panicle development. Two genes, *OsGAP1* and *OsPUB24,* showed higher expression in almost all tissues except for panicles, indicating their role in the vegetative growth of rice plant. The genes of group 2 in the remaining tissues showed differential, intermediate expression patterns (Figure 7). Our gene expression analysis concluded that most of the genes from group 1 were highly expressed in different stages of panicle development, while group 2 genes at endosperm and leaf development stages, respectively.

**FIGURE 8:**
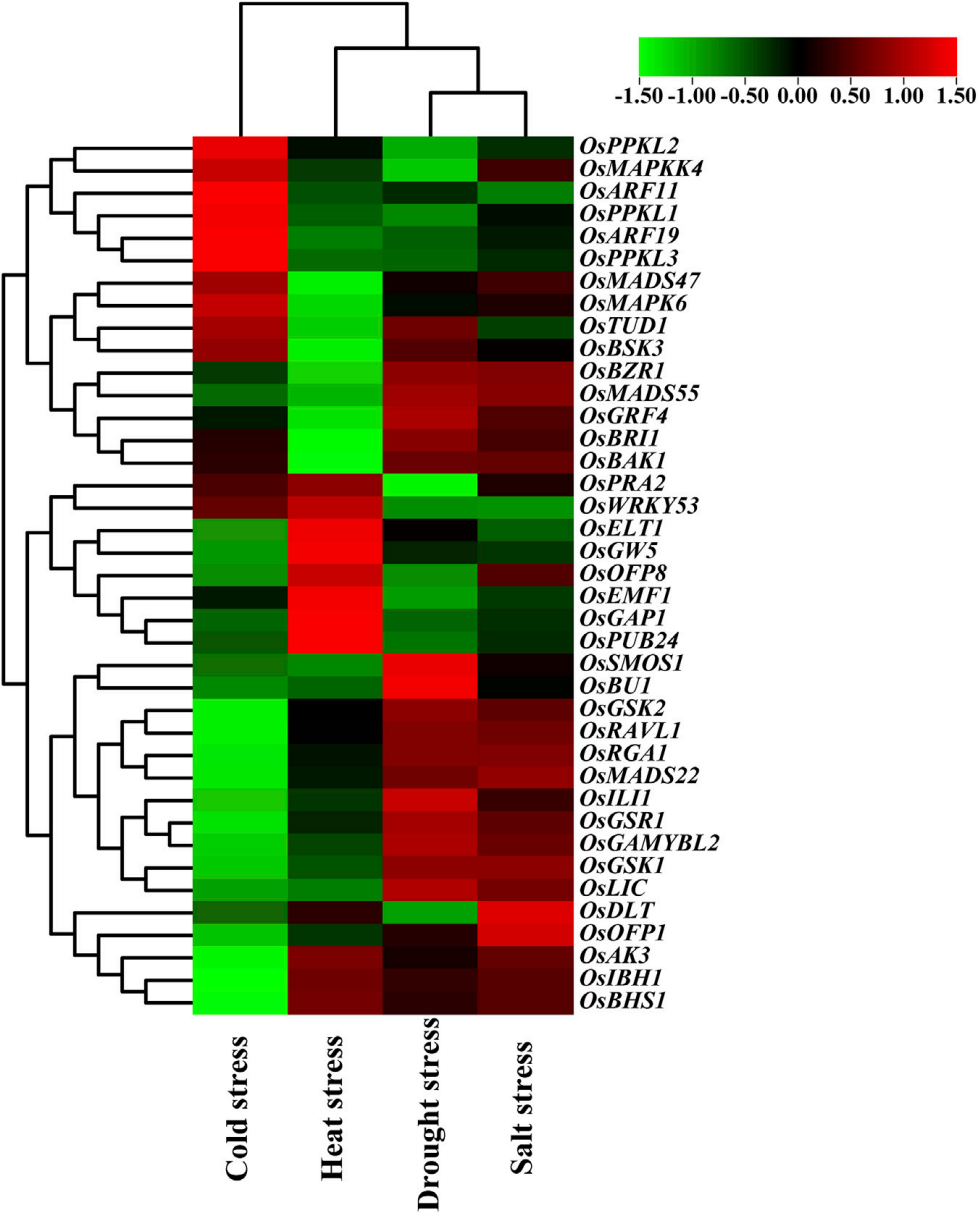
Expression pattern of the BR signaling genes in response to abiotic stresses.

In addition, we also examined the expression patterns of the BR signaling genes in response to various abiotic stresses (cold, heat, drought, and salt stress). Importantly, 19 out of the analyzed 39 genes showed opposite expression patterns in response to cold and heat stresses. The following genes: *OsPPKL2*, *OsMAPKK4*, *OsARF11*, *OsPPKL1*, *OsARF19*, *OsPPKL3*, *OsMADS47*, *OsMAPK6*, *OsTUD1*, and *OsBSK3* showed up-regulation in reaction to cold stress. Interestingly, these genes were down-regulated in response to heat stress. Conversely, the following genes: *OsELT1, OsGW5*, *OsOFP8*, *OsEMF1*, *OsGAP1*, *OsPUB24*, *OsAK3*, *OsIBH1*, and *OsBHS1* showed up-regulation during the heat stress response, however, they were down-regulated under the cold stress conditions (Figure 8).

As far as reaction to drought and salt stresses is concerned, the vast majority of the analyzed genes (except *OsMAPKK4*, *OsTUD1*, *OsOFP8*, and *OsDLT*) showed similar expression patterns in response to these stresses. Out of the analyzed genes, 20 and 22 genes were up-regulated in reaction to the drought and salt stress, respectively. On the other hand, 14 and 12 out of the analyzed genes were down-regulated in response to the drought and salt stress, respectively. Interestingly, a group of the analyzed genes, including *OsGSK2*, *OsRAVL1*, *OsRGA1*, *OsMADS22*, *OsILI1*, *OsGSR1*, *OsGAMYBL2*, *OsGSK1*, and *OsLIC* showed specific expression pattern—they are upregulated during response to the drought and salt stresses, but down-regulated under both thermal stresses (cold and heat). Noteworthy, the two major negative regulators of the BR signaling (*OsGSK1* and *OsGSK2*) represent the above-mentioned group of genes. Interestingly, the *OsBRI1* and *OsBAK1* genes encoding two protein kinases, which cooperate during formation of the transmembrane BR receptor complex, showed very similar expression profiles in response to the analyzed stress conditions (Figure 8).

### Protein-Protein Interaction Among the BR Signaling Components in Rice

**FIGURE 9:**
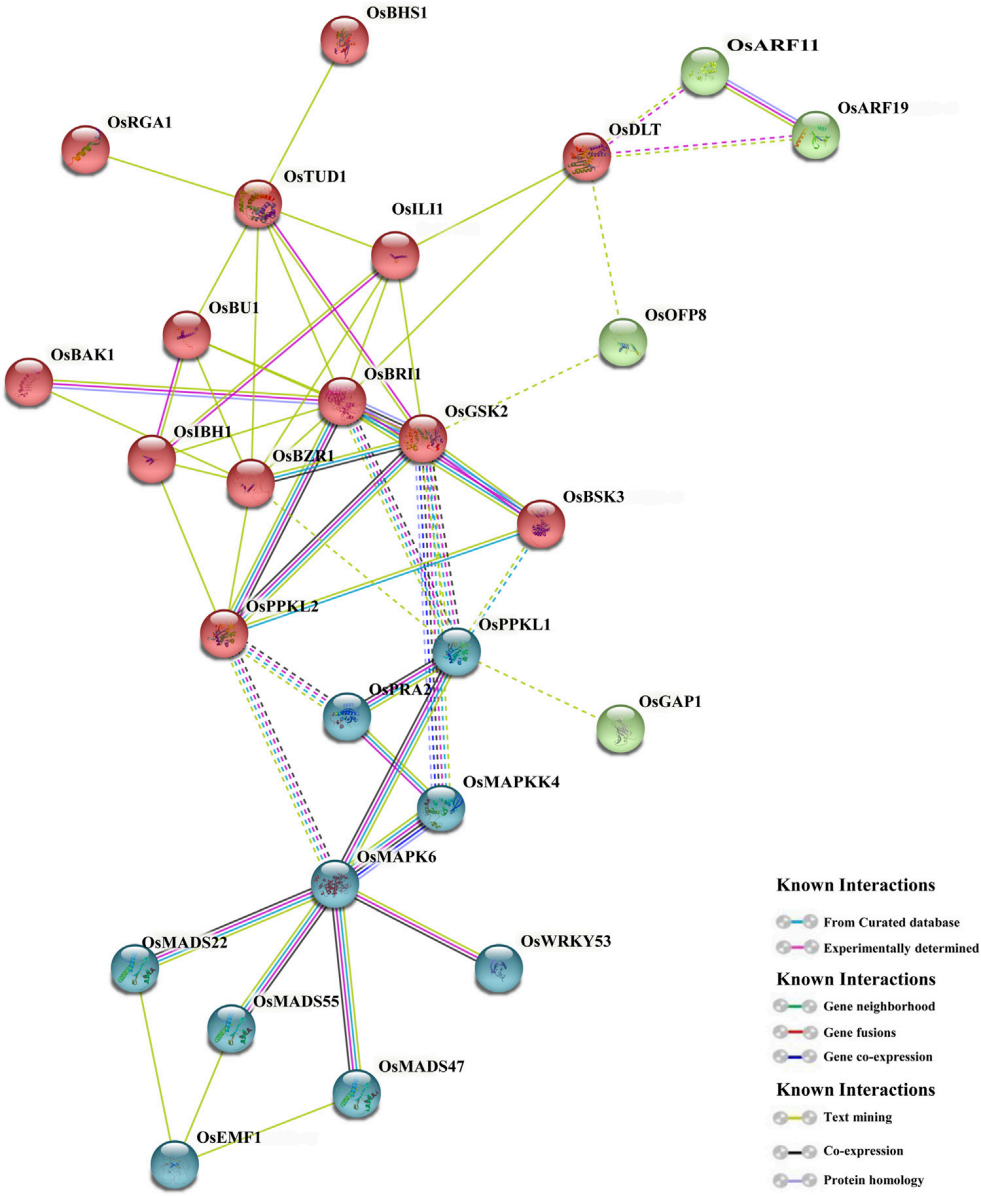
Protein-protein interactions among the BR signaling components in rice.

A model of protein-protein interaction (PPI) of 39 BR signaling proteins was predicted using the STRING database. The obtained result indicates that 26 out of 39 proteins revealed strong interaction, and the interaction had 37 nodes with 55 weighted edges followed by an enrichment *p*-value <0.01. The average node degree among the adjacent proteins was 2.97. Based on the PPI results, most proteins interact with more than one protein, except OsRGA1 and OsBHS1, which associate with OsTUD1, whereas OsGAP1 interacts with OsPPKL1. The interaction shows complexity within the BR signaling process and proves the versatile nature of the proteins (Figure 9). The results predicted complicated (and in some cases previously unknown) interactions of the analyzed proteins to regulate plant growth and development, stress responses, hormonal regulation, etc. Additionally, the detailed annotation of network proteins is described in (Supplementary Table S7), even though further investigation is needed.

## Discussion

The BR signaling pathway is one of the best characterized molecular relays in plants. Moreover, BRs are one of the most significant growth-promoting hormones and play an essential role in controlling yield-related phenotypic features, such as leaf angle, tillering, plant height, and grain filling in rice (Vriet et al., 2012; Sadura et al., 2019; Gruszka, 2020). However, our knowledge about mechanisms that regulate the coordinated expression of genes which encode the BR signaling components during plant development and reaction to various environmental stresses is still limited. Recently, Zheng et al. (2021) analyzed 11 rice BR signaling genes as queries to compare them with their orthologues from the Rosaceae family. The BR signaling-related genes from Rosaceae vary from their rice homologs in various characteristics, including gene length, conservation of domains and secondary structures in the encoded proteins, and reactions to external signals in terms of changes in their expression profiles. Therefore, in the present study, we used various online bioinformatics tools to conduct the *in silico* analysis of promoters sequences of 39 BR signaling genes from rice to find characteristics that can be used as a model for other crops, such as chromosomes in which the genes are localized, the total length of the gene in the rice genome, the number of introns and exons in each gene, and length of encoded proteins (Table 1, Supplementary Figure S1). Moreover, the results of our analysis provided vital information on the putative cis-elements and their corresponding TFs involved in regulatory mechanisms and interdependencies that influence the BR signaling during growth, development, and stress response. This kind of data may be important for interpreting the involvement of the BR signaling in the regulation of developmental and stress-adaptation processes in other monocots (including cereal crops).

### The *In-Silico* Analysis of Common TF Binding Sites Within Promoters of the BR Signaling Genes

In rice and other crop species, the TF families (NAC, WRKY, bHLH, bZIP, MYB, and AP2/ERF) are classified depending on their respective pathways (Lindemose et al., 2013; Das et al., 2019). We identified binding sites of several TFs (TCP, WRKY, bHLH, NAC, BES1, bZIP, MYB, GATA, and AP2/ERF) which are involved in the regulation of growth, reactions of plant to environmental stresses, and developmental processes within promoters of the analyzed BR signaling-related genes. Our results indicated that the most common TFbs in all 39 rice BR gene promoters was the TCP-binding site (4620 TFbs in total in all analyzed promoter sequences). These TFbs are helpful to regulate germination, plant growth and development, and reactions to the abiotic and biotic stresses (Li, 2015; Danisman, 2016; Lan and Qin, 2020). Our study indicated that the highest number of the TCP TFbs is present in the *OsMADS22* and *OsSMOS1* gene promoters (Supplementary Table S3), suggesting that they may be potential candidates for the regulation of plant development corresponding to the TCP TFbs. For example, the gene *AtTCP14* in *Arabidopsis* is involved in controlling gene expression during seed germination (Tatematsu et al., 2008). Overexpression of the *OsMADS22* gene leads to shorter stems and delayed senescence at maturity. However, knockout of this gene did not result in any changes in coleoptile elongation, lamina joint inclination or stem elongation (Sentoku et al., 2005; Lee et al., 2008). The *OsSMOS1* gene is necessary for *OsBZR1* function to rescue the dwarf phenotype (Qiao et al., 2017). Moreover, TFs such as *TCP14/15* have been shown to promote cell growth via the modulation of the expression of 9 genes during GA-mediated germination (Xu et al., 2020). Interestingly, it should be mentioned that the *OsSMOS1* and *OsMADS22* genes play contrasting roles in the regulation of BR-dependent gene expression. The *OsSMOS1* is a positive regulator of this process, whereas *OsMADS22* represses the BR signaling and response (Lee et al., 2008; Qiao et al., 2017; Gruszka, 2020). This indicated that the TCP transcription factor in rice may regulate the expression of these two target genes having opposite effects on the BR response.

The second largest and most common TFbs was the AP2/ERF-binding site (1756 in all analyzed gene promoters). These TFs are involved in several stress-responsive mechanisms in various plant species, because many stress-responsive processes in plants are closely associated with members of the ERF family. Therefore, these TFbs may be particularly common in promoters of stress-related genes (Lindemose et al., 2013). Several studies have shown that over-expression of the AP2/ERF genes, either in a root-specific or constitutive manner, enhances water use efficiency and various other environmental responses without affecting grain yield (Karaba et al., 2007; Yang et al., 2010). In the present study, the highest number of AP2/ERF TFbs was reported in promoters of the *OsLIC* (197) and *OsAK3* (194) genes (Supplementary Table S3), indicating that they may be involved in the coordination of plant growth and BR response during plant reaction to stress conditions. In addition, the expression of gibberellin-deactivating genes was upregulated in several ERF family candidates under high-salinity stress, leading to reduced levels of endogenous gibberellic and improved stress tolerance (Magome et al., 2008). It has been reported that AP2/ERF TFs stimulated the production of ethylene, salicylic acid, and jasmone acid in response to biotic stress (Dar et al., 2017). Since the AP2/ERF TFs promote accumulation of the stress hormones, the high number of the AP2/ERF binding sites in the promoter of the *OsLIC* (one of the major negative regulators of the BR response) may indicate that AP2/ERF enhances expression of the *OsLIC* gene during plant reaction to stress conditions in order to reduce response to BRs (which are mainly growth promoting phytohormones) and promote response to the stress-related phytohormones. The other TFbs such as bHLH (1145), bZIP (995), MYB (874), WRKY (593), GATA (585), NAC (281) and BES1 (33) were commonly present in 39 BRs signaling gene promoters. The highest number of the bHLHbs was reported in the *OsOFP1* gene promoter (142), whereas the bZIP TFbs were present in the highest number (93) in promoter of the *OsDLT* gene (Figure 3, Supplementary Table S3). The function of certain members of the bHLH family of transcription factors in rice is crucial for iron absorption and utilization, while other members of this TF family play a role in regulation of the anthocyanin and anthocyanidin biosynthesis (Sakamoto et al., 2001; Ogo et al., 2006). Furthermore, the bHLH TFs regulate gene expression during salicylic acid and jasmonic acid biosynthesis in agricultural plants. Their functions are essential to the regulation of stress responses, as well as the ROS scavenging mechanism (Huang et al., 2013). A recent study found that overexpression of gene encoding the BES1-Interacting MYC-like Protein1 (*OsBIM1*), which belongs to the bHLH family, in rice resulted in an increase in leaf angles. However, the T-DNA knockout mutant of *osbim1* and wild type showed similar leaf inclination (Tian et al., 2021). Interestingly, the *OsOFP1* transcription factor in rice is a positive regulator of the BR-dependent gene expression, but a negative regulator of the gibberellic acid accumulation. Thus, the bHLH-mediated regulation of expression of the *OsOFP1* gene may impact on responses to both phytohormones.

Another essential TF family, called bZIP, regulates several plant developmental processes, such as seed development, growth, plant maturity, and reaction to abiotic stresses (Jakoby et al., 2002). In our study, the presence of bZIP TFbs in all targeted gene promoters suggested it might be helpful for various regulatory mechanisms which influence growth, development, and stress response. The positive effect of transcription factors belonging to the bHLH family on the BR response in rice may also be mediated by the aforementioned *OsDLT* transcription factor (promoter of this gene includes the highest number of the bZIP TFbs) which positively regulates the BR-dependent gene expression.

The WRKY transcription factors comprise a conserved, plant-specific family that play a significant role in plant growth, development, and stress challenges, especially in drought stress in rice, rapeseed, *Arabidopsis*, and other species (Chen et al., 2022; Lee et al., 2022). In our study the WRKYbs were found 593 times in total, except eleven gene promoters, including *OsPPKL3*. On the other hand, the WRKYbs were present in the highest number (73) in the promoter region of the *OsPPKL2* gene. It is important to note that a neofunctionalization among homologous OsPPKL proteins occurred within the rice genome. There are three paralogs of genes encoding the OsPPKL proteins in the rice genome (Gao et al., 2019). Interestingly, two of these homologs, *OsPPKL1* and *OsPPKL3*, play a negative role in the regulation of grain length, whereas *OsPPKL2* has a positive effect on the grain length in rice (Zhang et al., 2012). Therefore, the significant difference in the occurrence of the WRKYbs within promoters of the *OsPPKL3* and *OsPPKL2* genes may provide a basis for this neofunctionalization. Similarly, our analysis indicated that promoters of genes belonging to another class, *ARF11* and *ARF19*, which encode auxin-response transcription factors, have a different pattern of TFbs. It is known that the *OsARF11* and *OsARF19* transcription factors constitute points of crosstalk between the auxin and BR signaling pathways. However, they are differently regulated by these hormones. The expression of both genes is induced by auxin, whereas only the *OsARF19* gene’s expression is stimulated by BR (Sakamoto et al., 2013b; Zhang et al., 2015; Liu et al., 2018). The above-mentioned results indicate that the differences in the same class of gene promoters may provide a basis for their sub- or neofunctionalization in terms of differential pattern of expression during plant development and/or reaction to hormonal stimuli. Another important TFs MYB plays an essential role in nearly all plant growth and development aspects and responds to diverse abiotic and biotic stresses (Ponce et al., 2021; Wang et al., 2021). MYB TF is the third most common TFbs present in all targeted gene promoters in the current study (Figure 3, Supplementary Table S3).

As far as the GATA TFbs are concerned, these TFbs were absent in four gene promoters (*OsBHS1, OsGSK1, OsELT1,* and *OFP1*) and present in the remaining 35 BR signaling gene promoters. There are still limited numbers of reports available about the role of GATA TFs in regulation of plant development and physiology. For example, role of these TFs in response to anaerobic germination is not well studied yet (Gupta et al., 2017; Mohanty, 2022). However, it is known that genes related with stress-response, metabolism, and hormone signaling are controlled by the GATA TFs (Gupta et al., 2017).

In the present study, NAC TFbs were present in 36 gene promoters which can be potentially used to reveal their biological significance. The NAC transcription factors are well-known plant-specific transcription factors and members of this family affect plant growth, development, and stress responses (Yuan et al., 2019). Six NAC transcription factors respond to Tomato yellow leaf curl virus (TYLCV) infection in resistant and susceptible tomato cultivars (Huang et al., 2017). Recent functional studies demonstrated that several NAC TFs function as positive or negative regulators of plant immunity to biotrophic, hemi-biotrophic or necrotrophic pathogens (Yuan et al., 2019). This may indicate that apart from being (mainly) growth-promoting phytohormones, BR may also regulate plant response to these environmental stresses.

Interestingly, the lowest number of TFbs (33) was found for the BES1 transcription factor (one of the major regulators of the BR-dependent gene expression). Moreover, occurrence of the BES1 TFbs per promoter (range 0–4) was the lowest among all analyzed TFbs. The BES1 TFbs were present in 16 promoters out of 39 targets, and the highest number (4) of the BES1 TFbs was present in the three (*OsBRI1*, *OsDLT*, *OsTUD1*) genes promoters. It is known that the BES1 (and BZR1) TFs coordinate the BR-dependent gene expression and constitute hubs of various interconnecting signaling pathways (Gruszka, 2018). However, an in-depth understanding of the BES1 function is lacking in rice and other monocot crops, especially when compared with *Arabidopsis* (Giovannoni et al., 2017). It seems that in *Arabidopsis* BES1 remains the major negative regulator of expression of the BR biosynthesis-related genes with relatively little impact on the BR signaling-related genes. Our results are in accordance with data gathered in *Arabidopsis* where BES1 negatively regulates expression of the *BRI1* gene in a feedback manner (Yu et al., 2011; Gruszka, 2013). However, in our study of the promoters of the BR signaling genes in rice, several other components of the BR signaling seem to be regulated in the BES1-dependent manner. It may suggest that apart from the fact that during evolution of monocots some components of the BR signaling emerged which are not present in *Arabidopsis* (Zhang et al., 2014; Gruszka, 2020), but additionally the BES1-dependent regulation of the BR signaling genes may be broader than in *Arabidopsis*. Therefore, our promoter analysis of 39 BRs signaling genes indicated that the presence of BES1 TFbs in 17 gene promoters might have a role in the BR-dependent gene expression which needs to be functionally validated. Additionally, results of our study indicated that promoters of several genes (*OsBRI1, OsBZR1, OsDLT, OsTUD1, OsARF11, OsGAP1, OsPPKL1, OsPUB24, OsBU1, OsWRKY53, OsEMF1, OsRAVL1*) contain all 9 common TFbs from our analysis.

### Identification of CpG Islands and Tandem Repeats Within Promoters of the BR Signalling Genes

DNA sequences that were not methylated in the plant genomes and rich in G and C nucleotides are classified as CpG islands. The role of CpG island in promoter region facilitates nucleosome remodeling and recruitment of transcription factors. The methylation status of CpG islands may be determined using several methods that assume the same methylation level over the whole CpG island (Barrera and Peinado, 2012; Villicaña and Bell, 2021). In this study we have found that CpG/CpNpG islands were present in 30 out of 39 analyzed promoters, except for *OsGSK1, OsGSK2, OsELT1, OsGW5, OsPRA2, OsBSK3, OsIBH1, OsMAPKK4,* and *OsAK3* (Table 2). The obtained results should follow the criteria previously explained by (Kaur et al., 2017). Those promoters in which CpG islands were absent suggest that gene expression is not repressed by cytosine methylation and/or gene regulation may occur via a different epigenetic regulation mechanism, such as post-translational histone modifications. However, this needs to be further investigated. Tandem Repeats consist of repetitive DNA motifs which help to regulate gene expression (Vinces et al., 2009; Reinar et al., 2021). We also checked the TRs in our targeted promoters and TRs were present in 17 of them with variations in length, suggesting that these variations could be due to the addition and deletion of TFbs (Table 3). The presence of TRs in these promoters can be used for mutational study, and it might have participated in gene expression regulation.

### *In Silico* Analysis of Common Cis-Regulatory Elements Within Promoters of the BR Signalling Genes

In our study 97 trans-acting regulatory elements were identified in all 39 targeted gene promoters (Supplementary Table S5, Supplementary Table S6). Several cis-elements, such as ABRE, ARE, AT ∼ TATA-box, CAAT-box, MYC, STRE, TATA-box, WRE3 and G-box were present in more than 70% of the analyzed promoter sequences, what suggested that these cis-elements play a role in regulation of expression of these genes during growth and development of rice and in response to light, hormone, and stress stimuli (Figure 4)*.* The stress-responsive cis-elements were common in all BRs signaling gene promoters. The stress-responsive elements were categorized into biotic and abiotic stress-responsive components. The abiotic stress-related cis-elements included the following motifs ARE, CAAT-box, DRE core, MBS, MYB recognition site, Myb-binding site, MYB-like sequence, MYB, LTR, MYC, STRE, TC-rich repeats, MBSI, GC-motif, DRE1, AT-rich element, whereas the biotic stress-related motifs included box S, WRE3, W-box, and WUN-motif. The GC-motif is an enhancer-like element engaged in anoxic-specific inducibility and the AT-rich sequence is connected to elicitor-mediated activation during stress responses (Brown et al., 2003). The Anaerobic responsive elements (ARE) motif is an essential regulatory element for anaerobic induction in plants. According to structure analysis of ARE, these bipartite components are made up of GT and GC motifs (Dolferus et al., 2001). MYB transcription factors are essential for plant growth, secondary metabolism, hormone signal transduction, abiotic stress tolerance, and disease resistance (Li et al., 2015). The drought-responsive gene regulation in plants is influenced by several cis-regulatory elements, such as the STRE, DRE, LTR, and MBS motifs (Niu et al., 2020). The W-box is a fungal elicitor-responsive transcription factor that interacts with WRKY TFs (Liu et al., 2016).

The second most common group of cis-regulatory elements which were present in the analyzed promoters were the light-responsive motifs (AE-box, G-Box, I-box, Sp1, Box 4, GT1-motif, TCCC-motif, TCT-motif, ATCT-motif, MRE, GA-motif, GATA-motif). BRs and light signals have essential roles in plant development and play an opposite role in controlling the transition from skotomorphogenesis in the dark to photomorphogenesis in the light (Cao et al., 2022). The presence of light responsive elements in the targeted promoter sequences which is reported in the present study, needs to be further investigated to check its correlation with the light/dark conditions. Numerous cis-elements, including G-Box, ACE, Box-4, Sp1, TCT-motif, and GATA motif, have also been previously found in promoters of the drought-, salinity-, cold-, and heat-related genes (Shariatipour and Heidari, 2020). In the present study, the G-box and Box-4 motifs were found the most abundant cis-elements of this category. These motifs exist in the regulatory regions of genes whose transcriptional activity is light-dependent. The Box-4 element is abundant in soybean WRKY genes, indicating that it is essential for light-regulated transcriptional activity. The G-box motif has been implicated in photosynthesis, hormone signaling (ethylene and ABA), and stress responses (Ma et al., 2013; Yin et al., 2013).

The hormone responsiveness cis-elements (ABRE, CGTCA-motif, TGACG-motif, TCA-element, TGA-element, AuxRR-core, P-box, ERE, ERE, and GARE-motif) were common in all 39 BRs signaling gene promoters. Among them, the ABRE cis-elements, which are frequent in promoters of genes which are regulated in the stress-inducible, ABA-dependent manner (Bray, 2002), were predominant. It suggests that the regulation of expression of the BR signaling genes in rice may proceed in this way (Figure 5, Supplementary Table S4). The bZIP TFs are mainly bound by the ABRE core, and several bZIP TFs are implicated in the regulation of the ABA-dependent stress response (Banerjee and Roychoudhury, 2017; Yao et al., 2020). Importantly, in the present study, it was also confirmed that bZIP TFbs and the ABRE core motif both were common in 32 out of 39 analyzed gene promoters. These results indicated that the bZIP TFbs and ABRE motif are tightly mutually connected with the regulation of plant development. This study indicated that presence of the bZIP TFbs and the ABRE core motif in gene promoters are also interdependent in regulation of plant development in the BR signaling-dependent manner. All promoter sequences contained hormone-related elements, including the ABA, salicylic acid, methyl jasmonate, and auxin-responsive elements (Banerjee et al., 2013). It indicated that the expression of the BR signaling-related genes in rice is regulated by a vast group of hormones, which most probably allows for inter-hormonal coordination of developmental and physiological processes.

### Expression Profiling of the BR Signalling Genes

Microarray methodology provides a rich resource for investigating the evolution of gene expression. The temporal and spatial expression patterns of the BR signaling genes provide useful information for establishing their putative functions (Zhao et al., 2010). Our microarray analysis indicated that the expression patterns of 37 BR signaling genes are differentially regulated. Based on clustering, 37 genes were classified into two groups (Group 1 and Group 2) (Figure 7). All genes from group 1 showed the highest expression at different panicle development stages and lowest expression at leaf and flag leaf stages. This would provide discrimination in predominant expression between generative and vegetative tissues. Interestingly, *OsGRF4* was preferentially expressed in developing panicles and the highest levels of expression were found in panicles of 7 cm in length. On the other hand, there was less transcript accumulation in the hull, root, stem and leaf sheath (Sun et al., 2016). The higher expression levels in panicles and low expression levels in roots were observed in a novel component of the BR signaling in rice—*OsBHS1* (Zhang et al., 2022) and in present study also showed a similar pattern of gene expression in roots and panicles. Furthermore, in the remaining tissues, such as stem, root, sheath, radicle, shoot, seed, and endosperm, the genes of group 1 showed intermediate expression except for *OsOFP8* and *OsBRI1,* which were highly expressed at the seed development stage. However, in grapevines, the *VviBRI1* gene showed the low expression level at seed development stage (Parada et al., 2022). In addition, we have found that the *OsRGA1* gene showed intermediate expression at all developmental stages. The *Rht* orthologue of rice *OsRGA1* gene in wheat shows a very low expression level in different vegetative tissues (root, stem, flag leaf, young leaf and leaf blade) at jointing stages and intermediate expression level in flag leaf, leaf sheath and spike at grain-filling stages (Chai et al., 2021).

The expression pattern of group 2 indicated that the *OsMAPK6, OsMADS55/22, OsPPKL1/2/3, OsBSK3, OsLIC, OsBAK1* and *OsGSK1* genes were highly expressed at the development stages of panicle, stem, sheath, and root. In contrast, the *TaPPKL1/2/3* genes showed lower expression levels in root and stem during wheat growth (Yang et al., 2019). Moreover, Lee et al. (2008) confirmed that expression levels of the *OsMADS55/22* genes indicate the diversified roles in age-dependent BR responses. Based on the gene expression analysis, we concluded that most of the genes from group 1 were highly expressed during panicle development, while group 2 genes were expressed at endosperm and leaf development stages.

Furthermore, in this study, the expression patterns of the 39 BR signaling genes in response to various abiotic stresses were analyzed. Importantly, 19 out of the analyzed BR signaling-related genes showed opposite expression patterns in response to cold and heat stresses. Thus, it may be inferred that the groups of genes which displayed the opposite expression patterns under these thermal stress conditions (Figure 8) may be regarded as representatives of the BR signaling pathway which play important role during adaptation of rice plants to the thermal stresses. It is known that BRs are mainly growth promoting hormones. However, it is also known that BRs regulate plant tolerance to the environmental stresses, but the underlying mechanisms remain largely unknown (Gruszka et al., 2018). However, several studies indicated that expression of genes related with cold tolerance may be increased by exogenous BRs. It has recently been demonstrated that the BR signaling genes play a critical role in the response of plants to cold stress. It was also reported that accumulation of BRs and dephosphorylated form of the BZR1 transcription factor might be induced by chilling (Fang et al., 2019). In *Arabidopsis* plants*,* overexpression of the *TaBRI1* gene, which encodes the BR receptor of wheat (*Triticum aestivum*), greatly enhanced their resistance to cold stress. It also increased the accumulation of dephosphorylated BZR1 during the cold stress, which led to an increase in the transcription of the cold response genes (*CBFs*), and consequent regulation of cold signaling (Singh et al., 2016). Interestingly, a recent study conducted in another monocot crop species—barley (*Hordeum vulgare*) indicated that mutants defective in the BR biosynthesis or signaling showed a higher tolerance to high temperatures, but reduced tolerance to low temperatures than respective wild type cultivars (Sadura et al., 2019). Thus, it may be inferred that genes encoding components of the BR signaling pathway may indeed participate in molecular mechanisms of adaptation to the thermal stresses, as it was shown in the gene expression analysis of the BR signaling genes in rice (our study). Effective adaptation of plant to the thermal stresses may be achieved through differential expression of both groups of genes which in our study displayed the opposite expression patterns in reaction to the cold and heat stresses.

Interestingly, in our study the vast majority of the analyzed genes showed similar expression patterns in response to drought and salt stresses. It may illustrate that fact that both stresses result in the same physiological consequence—a decrease in water potential within plant cells. Noteworthy, several genes showed the specific expression pattern—they are upregulated during response to the drought and salt stresses, but down-regulated under both thermal stresses (cold and heat) (Figure 8). It may indicate that this group of the BR signaling genes functions as specific regulators of the plant response to drought and salt stresses, and that different environmental stresses activate various subgroups of the BR signaling genes. It corresponds with the fact that proteins involved in various stages of the BR signaling pathway are interconnected with various signaling pathways of other phytohormones and stress responses in a crosstalk manner (Gruszka, 2018; Gruszka, 2020). Importantly, the above-mentioned group of genes which showed the specific expression pattern (drought/salt vs. cold/heat) includes two major negative regulators of the BR signaling—*OsGSK1* and *OsGSK2*. Involvement of the GSK proteins in plant reaction to abiotic stresses has been reported in several model and crop species (Li et al., 2021). In *Arabidopsis*, the BIN2 kinase from the GSK family stabilizes the TINY and RD26 transcription factors, which positively regulate the drought tolerance (Jiang et al., 2019; Xie et al., 2019). However, in the same species, the GSK proteins are negative regulators of the salinity stress response, with BIN2 playing the major role (Li et al., 2020). In rice, apart from being involved in the BR signaling, *OsGSK1* may also participate in the stress response. It was reported that T-DNA insertional mutation of the *OsGSK1* gene resulted in the increased tolerance to several abiotic stresses, including salinity and drought. It indicated that *OsGSK1* is a negative regulator of rice response to these abiotic stresses (Koh et al., 2007; Zhang et al., 2014). The different effects of the GSK proteins on tolerance to abiotic stresses in the various dicot and monocot plant species may result from subfunctionalization which occurred among the members of the GSK protein family (Li et al., 2021). However, our study indicated that the *OsGSK1* and *OsGSK2* genes are mainly up-regulated during reaction of rice plants to the drought and salt stresses, whereas their expression is down-regulated under both thermal stresses. Again, it may indicate that different environmental stresses may differentially regulate expression of various subgroups of the BR signaling genes what allows fine-tuning of plant reaction to the stress conditions.

In the present study different cis-elements associated with various stress responses were identified in promoter sequences of the BR signaling genes. The highest number of the stress-responsive elements was reported in the *OsRLA1/OsSMOS1* gene, whereas the lowest number was identified in promoter sequence of the *OsMAPKK4* gene. In the gene expression analysis the *OsRLA1/OsSMOS1* was highly upregulated under the drought stress conditions, which may be due to the presence of the highest number of stress-responsive elements. In contrast, the *OsMAPKK4* gene was down-regulated in reaction to drought stress, and it may be explained by the lowest number of stress-responsive elements in this gene. It may indicate that the stress-responsive elements which were present in the different numbers in promoters of the *OsRLA1/OsSMOS1* and *OsMAPKK4* genes are mainly associated with the drought response. Overall, our analysis provided insights into the role of many BR signaling genes during abiotic stress responses. This information can be employed in future studies which will be performed on other plant species.

### Analysis of Protein-Protein Interactions

In this study the network of interactions among the proteins encoded by the BR signaling genes in rice was predicted. Models of interactions among proteins participating in the BR signaling pathways were recently described for *Arabidopsis* and rice (Nolan et al., 2017; Gruszka, 2018; Gruszka, 2020). However, it should be emphasized that in our analysis, several newly identified components of the BR signaling pathway in rice were included, such as OsAK3 (Adenylate kinase) and OsBHS1 (kinesin-13a), which have not been analyzed in any previous study of this kind. The protein interaction model predicted in this study confirmed previously reported interactions among the BR signaling proteins (Gruszka, 2020), however, it also provided new information about the protein interactions which will be discussed below.

In our study, the newly identified component of the BR signaling in rice, OsBHS1, was predicted to interact with OsTUD1 (Figure 9) which functions as a co-activator of the BR signaling relay which may be parallel or partly overlapping with the main OsBRI1-mediated pathway (Sakamoto, et al., 2013a; Hu et al., 2013). Moreover, in our study it was predicted that the OsTUD1 protein interacts with the OsILI1 transcription factor which inactivates through heterodimerization the OsIBH1 transcription factor, which functions as a negative regulator of the BR response (Zhang et al., 2009; Zhang et al., 2014). Although it was previously reported that the OsRGA1 and OsTUD1 proteins initiate the BR response process (Sakamoto, et al., 2013a; Hu et al., 2013), however, the downstream stages of this pathway remained largely unknown (Gruszka, 2020). Our study indicates that the BR response pathway, which is initiated by the OsRGA1 and OsTUD1 proteins, may proceed through the newly identified OsBHS1 protein and the OsILI1 transcription factor.

Moreover, our analysis indicated that the OsDLT protein which functions as a transcription factor which positively regulates the BR response in rice, may interact with the *OsARF11* and *OsARF19* transcription factors. It is known that expression of the *OsARF11* gene is induced by auxin, and the *OsARF19* gene expression is stimulated by auxin and BR. Thus, the *OsARF11* and *OsARF19* transcription factors constitute points of crosstalk between the auxin and BR signaling pathways (Sakamoto et al., 2013b; Zhang et al., 2015; Liu et al., 2018). The results of our study indicate that the crosstalk between the auxin and BR signaling pathways may be also mediated by the predicted interactions between the *OsDLT, OsARF11,* and *OsARF19* transcription factors.

Additionally, our analysis indicated that the OsPPKL proteins (OsPPKL1 and OsPPKL2) which function as negative regulators of the BR signaling in rice, as they enhance stability of the OsGSK proteins, may interact with the OsBSK3 protein which represses activity of the OsGSK proteins (Gao et al., 2019). Interactions between the positive regulators (OsPPKL1 and OsPPKL2) and negative regulator (OsBSK3) of the OsGSK proteins’ activity have not been previously reported (Gruszka, 2020). Therefore, the results of our study provided novel information about this aspect of the BR signaling. Moreover, the results of our analysis indicated that the OsPPKL proteins may interact with the OsPRA2 protein, which is a negative regulator of the BR signaling initiation (Zhang, et al., 2016). Previous report indicated that activity of OsPRA2 is stimulated only by the OsGAP1 protein (Song et al., 2017). The results of our analysis indicate that regulation of the OsPRA2 protein may also be mediated by the OsPPKL proteins.

Finally, results of our study indicated that the *OsMADS22, OsMADS55* and *OsMADS47* transcription factors may interact with the OsMAPK6 mitogen-activated protein kinase (Figure 9). It is known that the OsMADS22, OsMADS55 and OsMADS47 transcription factors are negative regulators of the BR signaling (Duan et al., 2006; Lee et al., 2008). However, so far molecular mechanisms regulating activity of these transcription factors were largely unknown (Gruszka, 2020). Moreover, according to our predictions the *OsMADS22, OsMADS55*, and *OsMADS47* transcription factors may also interact with the *OsEMF1* transcription regulator, which previously was reported to repress expression of the *OsMADS58* gene in rice during palea development (Yan et al., 2015; Zheng et al., 2015). However, other functions of the *OsEMF1* transcription regulator were not known (Gruszka, 2020). Thus, results of our analysis provided novel and interesting information about putative protein interactions during the BR signaling in rice which may become an input for further functional analyses in this and other species.

## Conclusion

BRs signaling genes play an important role in the growth and development of plants. However, a comprehensive analysis of promoter regions of the BRs signaling genes has not been performed. Therefore, in the present study the *in silico* approaches were followed using different bioinformatics tools for a comprehensive analysis of 39 BR signaling genes in terms of their chromosomal distribution, phylogenetic relationships, transcription factor binding sites (TFbs), cis-regulatory elements, and identification of tandem repeats and CpG/CpNpG islands in promoter regions. Additionally, expression patterns of these genes in different tissues during rice development and in reaction to several environmental stresses were analyzed. These analyses revealed the presence of different types and frequencies of TFbs and cis-elements in each gene promoter. Microarray data indicated that up or downregulation of the BR signaling genes is necessary in some aspects of plant growth and diversifies vegetative and generative organs. Our analysis also allowed for predicting differences in the BR-dependent expression of the BR signaling genes between *Arabidopsis* and rice. Moreover, the significant difference in the occurrence of the WRKYbs within promoters of the *OsPPKL3* and *OsPPKL2* genes, which regulate grain length in rice in opposite manner, may provide a basis for this neofunctionalization. This is the first report on *in silico* analysis of the BR signaling genes in *O. sativa*. Although further research is needed to clarify these complicated aspects, results of this study provided insights into various regulatory mechanisms and interdependencies which involve the BR signaling genes and influence growth, development, and stress response of rice and potentially other cereal crops.

## Data Availability

The datasets presented in this study can be found in online repositories. The names of the repository/repositories and accession number(s) can be found in the article/Supplementary Material.

## Supplementary Material

Supplementary File S1 Sequences of the 1.5-kb upstream promoter regions of the BR signaling-related genes in rice.

Supplementary Figure S1 Structure of the BR-signaling genes: blue boxes represent the untranslated regions, yellow boxes represent exons, and black connecting lines represent introns.

Supplementary Table S1 Gene annotations of the 39 BR signaling genes in rice.

Supplementary Table S2 List of all transcription factor binding sites localized in promoter sequences of 39 BR signaling genes in rice.

Supplementary Table S3 Common transcription factor binding sites (TFbs) present in promoters of the BR signaling genes in rice.

Supplementary Table S4 Detailed information about the cis elements which are present within promoters of the 39 BR signaling genes in rice.

Supplementary Table S5 The most common cis element presents in promoters of the BR signaling genes in rice.

Supplementary Table S6 Detailed information about the conserved motifs identified within promoters of the BR signaling genes in rice.

Supplementary Table S7 Mapping of the protein-protein interactions.
