# Supplementary figures and images for: In-Silico Study of Brassinosteroid Signaling Genes in Rice Provides Insight Into Mechanisms Which Regulate Their Expression
Source: Front Genet. 2022 Jul 6;13:953458. doi: 10.3389/fgene.2022.953458 (PMC9299959; doi:10.3389/fgene.2022.953458)

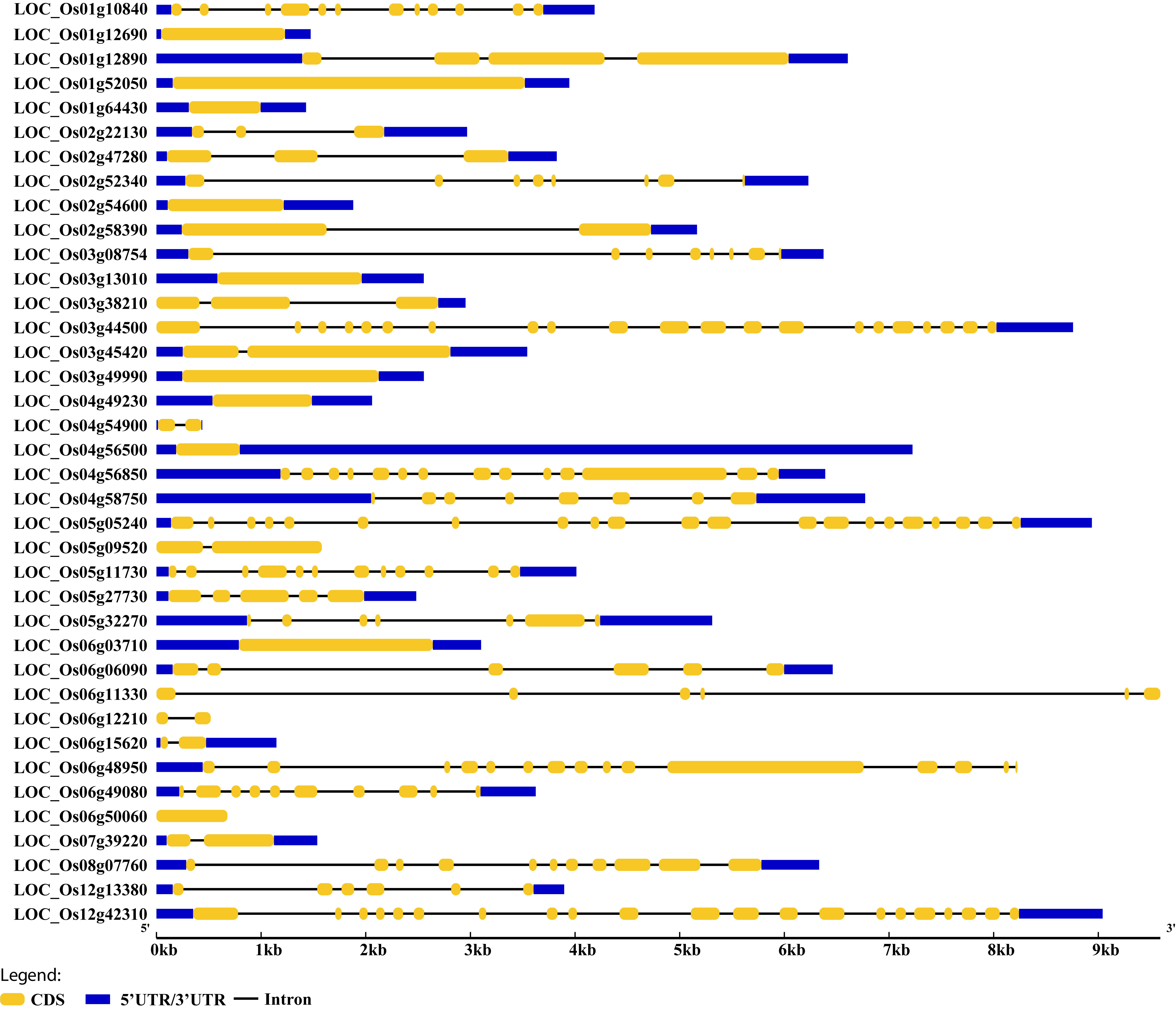

Supplement: Supplementary file 3 [file Image1.JPEG]
