# Supplementary material for: In-Silico Study of Brassinosteroid Signaling Genes in Rice Provides Insight Into Mechanisms Which Regulate Their Expression
Source: Front Genet. 2022 Jul 6;13:953458. doi: 10.3389/fgene.2022.953458 (PMC9299959; doi:10.3389/fgene.2022.953458)
Supplement: Supplementary file 9 [file DataSheet1.DOCX]

>LOC_Os01g52050

GGGTGATTTTATTTTCTAGTGAAGTTATTATTTGTCAATATGTAATTTATCATTTATATT

TTGATGAATTGAATATATAAAGTTATTTTAATTTTACATATTAATTATTTGTTATTACTT

TTTTTAATGATTATAAATTATATAGCTTAATGGAAGGCTCGCAAGCCAGCTCGAGCTAGC

TCGAGCCACAAACGAGCCGAGCCAAGCTCATGTTTTAGCTCATTGCTATAACCGAGCCGA

GCCAAGCCGAGACTAGCTTGTTGCGAGCCATTGCAAGCCGAGCCGCTCGGCTCGTTTCCA

CCCCTAGTGACAAGTGACAACGTCGTCGTGGGCATATTGGGGAAGCGGAGCACCCTGAAA

AAAAGGTCGCACGCCGCAGCCTCCACCGCACGCTTCTTCGGCTCCCGGATCGGATCTACC

ACCCCACTTCGGAAATTAAAGACAAAAAACCCTTCGCGAAGCAACGCAAGCCCACGCGTC

CGGTTCCCTCCACGAGTCCACGCCCGCCCAGGGATTCCGCACGCTCGGAACGCGACCCGA

GCCCATCCGCGCGAGGCCACACCACAGGCGTGAAATCCCCGCGCTGCCCACGAGCCCGCC

GCAGCGCCGCGCGAGGGCAGTCCGGTCAATCCGGCGCGCAACCCTCCCCCTCGCGACGGC

ACGTGACCGCGCCTCGAGCAGACCACCGGCTGTCCCCGGCCGTCGGATCGGATCGGGATT

TAACCTCGCCGTAAGCCGCGGATAAGCGCGGGGGATTAGAATACTTAACCTCTCCTCTGC

TCTCCTCCTCACCCGGCTTAAGCGCGCGGGGGGGCTGCGATTCGCAGGCGAGAGCACATG

CCATGTGACCCCACCCCACCACTCCTCCCTCACCTACAGCTGTTAAGGCCAGTCACAATG

GGGGTTTCACTGGTGTGTCATGCACATTTAATAGGGGTAAGACTGAATAAAAAATGATTA

TTTGCATGAAATGGGGATGAGAGAGAAGGAAAGAGTTTCATCCTGGTGAAACTCGTCAGC

GTCGTTTCCAAGTCCTCGGTAACAGAGTGAAACCCCCGTTGAGGCCGATTCGTTTCATTC

ACCGGATCTCTTGCGTCCGCCTCCGCCGTGCGACCTCCGCATTCTCCCGCGCCGCGCCGG

ATTTTGGGTACAAATGATCCCAGCAACTTGTATCAATTAAATGCTTTGCTTAGTCTTGGA

AACGTCAAAGTGAAACCCCTCCACTGTGGGGATTGTTTCATAAAAGATTTCATTTGAGAG

AAGATGGTATAATATTTTGGGTAGCCGTGCAATGACACTAGCCATTGTGACTGGCCTAAT

TGCTCTCCCCCTCTACTGTGTTTGCGTCTTTCTTCTTCGCTTCTCTTTCTCTCTCTCTCC

ATCTCCTCCTCATCACTTCCCACTCTCCCCCTTCTGTCTCTCTACTTTCTCTCTCTACCG

CCGCTCTCGCAGCAGGCCAGGTTCTCTCTAATGGTCGTGAGGCAGTGAGCTCGCTCGTAC

>LOC_Os08g07760

TTTAGATATTTCCAAAAAGGTATACATCAGATAAATTTGGTCCATGTTTCATTATAAAAA

TTGATATCCTACTCACATATATTTAATTTATCACATCTACTATATGCACTATAAAAATTA

ACCAATTGTAGAAAATCTCAAGTTTATAAAATAGTACTCTTAAAAATAGATCCAACCGTA

CGATGATCATCAATGGAATTAGATAAAACATGATTACATATTCATCGTTTCTCGCCGTTC

ACACGTTTAGTCACAAGCCCGGACGTGATTTAAAATTTCTCTTCACTGGTTAGTTCTTTT

TTTACTACACTTTTTTAGTGATTTTTTATATGTTGGATTAGAGGGAGAAAGGAAGGAAAA

TAACTATGCTTTTTAACATATACTCACTTCGGTTTCATAATTCTTATCATTTTGGATAAT

GACATGGTTTTCAAAATATATCTTTGACTGTATTTTTCTATTGTAATATATCTTTGACTG

TATTTTTCTATTGTAATACAGTTATACTTTAACTGTAGTACTCCCTTCATCTACTTTTTG

ATAGTCATATTTCATATTGGCACACAGACCAAGGATAAGTAATTCATCATCTATTTAAAC

ATGCTACTAGTCATTCCTCGTAAACAAGCGATTCATTAATATTTACATTTTTTAATACCC

ATGTAGCCAATCATGTGTGGAAGAATGGAGAGTCACACATTAAATCCGAGAAAGTCATTA

AGATGATAGGTTGTTGGATTGAAATATGCCTATCAAAAATAAATTTTTCAGATTTGGAAA

TATGACTATCAAAAATAGATGGAGGAAATACTTTGTAAGACAAGTCTATATATTGTTCTA

GTGACTTTAAACTAAATATTTTAAAAGTTATTAATAGTTAAAGTTATAAAAGTTTGACCT

TAATCTACAGTAGAAGGCCACACGCCCACACAGGCCACATCCCTCCTGTGGCTGCTGTTC

ATCCGACCACACGGGATCCCACCTCCTCCACCGATCCCGTTCCGTTGACTCCGTTCCCGT

CCCGCCCCGCCCCCACTCATCTCCACTAATCCCCTTCGCTAATCACCTCTGACTCCCTCT

AATCCACCATTTTAATTAAACCCCAATAATTAACAAACCCCTCACTCTCTTAATCCGCAT

TAATTAATTGATTAAAAAAATCTAATCACTCTCGCTATCCCGTTCTAATCCTAACCCCAC

CCGTGTCTCCTACCTCATCGACGGTATAAAGCGAGGCGACGAACTGACTCGACTCAACTC

AACCCCCCCCAAAAAAAAGAAGAAAAAAAAGAAAAAGAAAAAAAAGTCGTCGAAAAGCGA

GAGAAGGGGATCGATTCGAAGAGAGAGGAGGTAAAGCGGCGGCGGGAGAATCTTCGCCGG

ATCGGCGGCGGTGGCGGAGGAGGAGGAGGAGGAGGAGGAAGAGGGTGGTGCTGATTTGGT

GTGGAGGCGGGGAAATGCGTGATGTGTGAGATCTAGGGCGTGGGGAGGCGGATCGCGGCA

>LOC_Os01g10840

CCATAAAATTCATGCTAAAGACTTACCGCATCTTTAATCTTCTTAGTTTCTTTTGGCCCT

AGTTTTTTTTAATCTAGATACAACAAAAAAAAACAACAATTATAATGAAACGAATACAAT

CACATTTTAATAAAAATATCGCTATGTAACATGACTAAAATATACAACTTAAGCATCCAT

ACAATTTATTCTAAAAATGTACCACATCTATATTCTTCTTGACTACTTTCAACCCTAACA

TTTTATCCTGATACAACCTTAAGAAAGCAACAATCACGATGAAACGGATAAAACCACATG

TTAATAAAAAATTACTACGTAACCGTCGTTGAACTATACAGATTAAGCATCCATAAATTT

CATGCTAAAGACGTACCACATTTCTATTCCTCCTGACTATTTTCAGCCCCAACTTTTTAT

CCTGATACAACCTTAAGAAAGAAACAATCATGATGAAACAGATAAAACCACATGTTAATA

TAAATATTACTATGTAACCGTTGTTGAAATATACCAACTAAGCATCTATAAAATTCATGC

TAAAGATGTACTACATCTCTATTCTTCTTTATTTATTCCGGCCCAACTTTTTATCCTGAT

ACAATCTTAAGAAAGCAACAATTACGATGAAACGGACAAAATCACGTGTTAACAAAAATA

TTTCTACGTAACCGTCATTGAAATATACAAATTAAGCATTTATAAAAATCATGCTAAAGA

CGTACCGTATAGTTGTTTCTTTCTGTCCCAACCTTTTATCGTAATACAACCTAAAGAAAG

CAACAATCACGATGAAACGGATAAAAACATAAAACCATATGTTAATAAAAATATTTCTAC

TTAACAGTCATTGAAATATACAAATGAAGCATTCATAAAATTCATGCTAAAGATGTACAA

CATCTCTATTCTTCTTGACTTATTCTAGCCCGAGCTTTTTATCCCGATACAACCTTAAGA

AAGCAACAATTACGATGAAACCGATAAAAACCGTACGATGATAAAAAATATTTCTATATA

ACCGCCATTTGAACATACAAATTAAGCATCAACAAAATTCATGCCAAAGACGTACCACAT

CTCCATTCGTCATCCATACCCAATTCTTTATCCTAATCAATACCACCAAAGACTCCTTCT

AAACTAAGAAACTCAAGATTTTCACCTTGGTTCATCTCTCATGCAAATCCTCTAAAATTC

CTCCTATAAAACATCCCAGTTCATGAAAGAAAACCCTTCAAAAATATCATACTGAGCACA

GTTATTAGCTGGAGGAGTCAGCACAGTTATCGGTAAAACCACTTCCCCATCTGTCCAAAC

TCCAAAGCCGGCCTCACCGGCAGCAGCCACACCACACCACACCACAACACCCCGCAGCCT

TGCTGCCTCCCCTCCCTGACCGGTCAAAAGCACACCACCACCATCCCCCCGCAAAGCCAA

GCACCAACGCTCAGCAGCAGCAGCCGCCTCCCTCCCCAGCTCAGCTCAGCTCCCCCCCGC

>LOC_Os05g11730

AAAATTATATTTAAAAGTTCTCTAAATTTATATAAAAATTAATAGAGATAAATATAGACA

TGAAATACATTTACATCAAATCTCAAGTCGAAACTCAACTTTTATTTGAGAGAATATAAA

AGACAAATTTTAGGTGAATAGTGTTCTATTATTTTTCATCCGAAATTTGGCATTTTTGTT

ACTCCCAAATAAAGTTGAGTTTAACTTGATATTTGGTGAATATATTTCATGCCTACACTT

CTCTCCAGTAATTTTTCATGAATTTATTAAACTTTTAGTTCCGATTTTCACGTGTTTTCA

CTTATTTGATGGTTTCCATCGGATATGTCCCTGAAATATGGTATTGAAGTACAACATAGT

TCATACTTGGGTCGTGTTTGAAGTCATGTAAGGGCGTTATAAGCTCATAGGTTTTGCTTA

CATACAATTGGTGGGATAAAAAGGCACCGGTAATTTCTTCAAGATTGATAAAATAAATGT

CTAGCGCTATAAGGCCATGGCACACATCAAATGTTGTTTAGAACAGGTTATTTCTAGCTC

CATAAATTGTTGGATTTGAATTTGTGATTACATTGATAATAATTGATTGAATCAGTTTGT

TCTTATTTTAGAGAAAATAAAAAAAATAAACCACTATAATTTAACTTACAAACTCCAAAA

CTAGTCTGGATCTGTAATTTAGGTTGTGCTAAACAAGGCCTAAAGAAAAGAAGTAATGTT

TGGAGAACATGTTTTTAAATCAATATGGACCATCTTCTAAAATGGGCACACTGTGCAACC

GAAATGGTTATTAGCAACTTAATATCCAATCCTTAAAAAAACTACATTGAAAATATCCTA

AATCCCAAAATTAAATTTTAAAATCTAATTTTGGTAGCGATTGATTATTTGTAGGGGCAA

ATGATGAGGCCCTAAATCAACCATGTTTAGCTACTTCCTCGCTTTCTTTAAGTATGTTTC

ATACGCTACAAACTGATATTTTTTGCAAATACTTTTTATTAAAAAAATTATTTTAAGTCT

GCAAAAGCTAATGTTTAATTAGTCCTACACTAATAATCCTCCTTGTTTGGCTTGCCGCTG

ATAAGCTTAGTCAAAACCCTGATCCGAACTGCACGTAAGAACGGTCAAGAAACCATTTCG

GTTACATCACACAACACAGCCTCATCTCTCATGCTGTCATGCTTGTGGTGCACCTAGCAA

TTCCTCCCTCCCCATCTGTCTTCCTCCTCTAATCTAATCCACCTCCCCACTAATCCACCA

GCTGTGTACACTGCAGCAGCAGCAGCAGCTAACCACTCTCACTAAAAACTATAGCAGCTG

CAGTAACAGCAGCAGCATCACCCACCTTCTTCTTGGTCAAAGCCATCCATCCCACCACTC

ACCCATCCCTCCCAGTATAAGCCAAACCAATCCATAGAGGAGGAAGAGGAGGACCAGGTG

GTGGCACACCTAAGCTTTGTGCAGTGCCATTCACGCACCTGCAGCTTCCAGCTTTGCCAC

>LOC_Os07g39220

CTTGCCGTGGCCATCGGTCCTTCAACCGCGGCGTCGAATTCTGAATCCAGCCCGTTGATG

ATGTACGCCACCAGCTCCTCCTCATCCAGCGGCTTCCCTGCAGCCGCCATCTCGTCGGCG

AGGGACCTCATCTTCGCAATATATTCGCTGATAGACATAGGTCCCTTCTGTGTGGTGGTT

AAGGCGAGGAGTACATTGATTGTACCCGCCTTCGACTTGCACGAGAACATGTCATCAATC

ATGTTCCATGCCTGGGCCGCCGTCCTAGCGCCGGCCACTTGTTGCAGCACCTCCCTTGAT

AGAGAGGAGAAGATGAATCCGAGCACTTGCTGGTCCTGAACAAACCATGTCTTGTACTCA

GGGTTGGGGATGACAATCTTGGTCTTCTTGTCCTTGTCGCCGTCTTCTTTTTCAATTTCA

GCCGCCGGAGCTGCGGTCGTGCTGACGATGTGCTCCTCCAGCTGCGCACCTCGCAAGGTG

GTGAGGATCTGTGCAGCCCAGAGAGGATGATTCTGTTTGGTTAGCTTCTCCGAGATTTGG

AGGCCAAACAGAGGGTTGGAGATTGCCGCCGCCATAGAAGAGCTCGCCATGTCGCTGGGA

GAAAGTTTGCACGACAGAACTAATCTAATCTAGCGGGAGGCTCTGATACCATGTGGAGAT

AACTCCTTCAGCGAGGAGTTTACGTGTTAATTACTTGCCACAAAACCGGGCTTAACAGAA

TACAGGTATATATAGCTCGGATAGAGTTGTTGTGCATCTACTAAAACATAGGAAACTTAT

CTTATCTATCTAAACAGCCCAACAACTCTTATCCTTCGCTATTTGTTACATTATTATATG

TGAGAAATATCTAACACTACTCCACACTGTCCACAATTACTGCGAACCCGCAACGCACCA

AAAGCCCCTACGCGCCACTCGCAGTTTAGCACGACTACGAGATGAGAGCACGTAGCATCT

ACGAGCACATGCCACGCATGCACCACAGTCCACAGTGCTATGTACTACTCCCACCTCACT

CACCCGACGCACGCAGCGGCACAGAGCCAGCCAGCCACCCACAACACACACATCACACTG

GCCGTCCTCCTCAAAAGCCGCGCCCTGCCTGGGAAGGCGGAGGAAAACGTGGGTCCGAAG

GTGGGGACGCCACCACCACCAGTAGAATCTAGCCAATCCAGTTGCAATAAAGTATGCCCC

TCCCGTACCCTCCTCGTGAGCTCGCCCACTGCGTCCCCACCACCCATACACGCCGAAGCC

CCACGAGAGGGAAAGCACGCTACTGCTACTCGTCACCGCAGCGCACGCCACTAGACTAGC

TAGAGCTAGCTGGCTACGCTACTCTACTCTCCCCTCCCTCTCATTAGTCAATACTGTAGC

TGTAGTAGTGGTAGCACCCAACACCTCGCCATTAAAGAAACTTGAGCTCGCCAAGTGGAG

AAAGGAGAGGTGGAGGAGAGGAGGGGAGGGGAGGGGAGGTGAGGGACGGAGCTGGTGGGT

>LOC_Os06g49080

CTTCAACTGTACCTGCTGCTTCTCCTTGTCAAAGCTTTCCTCATTGCCCATCTTCTCCAT

CCTTGATGAGATTGCAGACATCCGAGCACTCTGCCAAGCTAATTGCATATGAAACACATA

AATATATAATCAGAGTGGAGCTCACATAGTACTAGGACTGCACAATTGAATTGAGCTATT

CTGAGAGGTGCAATGGCGATAGAACAGGTACCAGTACTGCCGGCTGCGGAATTGTCCCTT

GAAAACTACCAGAGGCCTGCAGGTTTAACAGTAATTGAGCTCGAATTATTGGCGAATGAA

CATATTTTCTGCAGGAATTCAGTGAACATATTTGATCAGGCTACATGAGATTGAGAGAGG

GTACTGCTACTTTCTGACGTTGAGGGGGCCCTCGCCGCCGCGGATGCAGGTGGCGACGCA

CTTGGAGGTGCACTGCAGCGCCTTCGCCGACTTGGGCCGCTTTATCCTCACCTGCTGCTT

CCCGTCGCCGCCTGCAAAATCATCCCCACACCCACCTCGTCGTCGTCATCGATCCATCCC

CCAGCAAAAGGCGTGAGAAAAACCGCGAGAGATCGGTCTCGCTTACCGGAGAGCGTCTGC

GGGATGTTGGAGAAGGTCTCCGGCTGCGGCGGCGGCGTCGAGAGGATCGCCGCCAGCGCC

AGCGCCACCACCGCCGGCGCCGCCGCGCGGGCCAAGCCGCCGCCGTTCATCCTCTCCGTC

TCCGACGACGCAATCTGCGCGCGCGGGCGCGTGTGTGGCGGGCGCTGCGCCTCTCTGTAG

CGGATTTTTTTTTCAACTTTTTTAATTTTTAATTTTAGAAATAAACATTTTCAAAACTTA

TTTTTAAAATATAAACTTTTGATGACATGAGCCCGCCTAGAATGGCAAAACAATATTGAC

ACGTCCCATACTGCAGACCTGACAGTTTTATTATGCTCGTCAGCTAAGATGGTGTGAATA

TGTGGTGAAATAACGCTACCACACGACGTGGCCAAATAACGGTGTCACGCTAGTTAGATT

GGTGTGTAATGTTGCCACGTTAGTCCGGTCGGCGTAGTAAAAAGGTTCAGATTTAAAAAT

AAGTTTTGAAATGTTTGTTTTTAAAATTAAAAATTTAAAAAGTTCAAAGAATAAAAAAAT

CTCTATGTATTGGTGTTGGTGTTGGAGCGCGCTACGGCCGTTCTATCTGGGCCGGGCCCT

CTCGTGGGCCGAATCACCCGCGGCCCGCCTGGCCCACTGGCTGCTCGCCGTCGTCACCGC

CTCTCAAAAGGGAGGAGGCACGACTACTCCGGCCGGCGGCCGCGTCTCGAGTTCTCGGCG

GAGGCGGCAAGCCGGCGAGGGCAACCCACCCACCGACGTCGTGCGGCCGCGCTTGCTGCG

TCGCGTGCCCACGCCAATCCCGTGGCGGCGGAAGGAGGAGGAGCAAGCGCTTGGGAGGAG

CTAAACCCTAGAGGCGGAGCTCTAGAATCGAATCCCCCCCCTTCCTTCCGCTTTGCGGCG

>LOC_Os06g03710

CAAAACTCGAGCTTACTTAGTAGCCAAAACAAATATACCAGCAGCTAACCCAGCTAATGA

CAATAGCTGTCTGCAAGTAAAACACCCGGTATTTTGTCACGTACCAAAATGACACTTTTT

TTTCTGACAGCAATGGCACATCATTAACTGAGCCCCGCATTGACGCCAAAACACGGGACA

CGCATGCGTGCGTACGTGTCCCGATCCGACGGTGGCAAACACACAAACATCATCATCACC

AGACACGCAGGATACAGCCACGTAGTACGTCGTACAGCACACCGTACCCCACCCTGTACA

CTAGCCTGTACGTACAGCTCAGCGTCCGTTGCCGCGCTGACGTGGCCGCACCGCGTGGCG

TCCGGCTCACGCGATCTGGGCCGTCGGTTCGGCGCGGGGGCCCACGCGTCAGACACGCGT

GTCAGGTGGTGGGAGCCCAACGGTAACAAAAGAACGGGGGCGTGCTGCGTGACCGCGTCC

ACGCGTGCGTGACAGCGTTAGGGGGGGTGGTCACGTGCGTCGCACCTGACCGCAACGGTC

ACATTGACCAGAGAGGGGGGGAGGCAATCAGGCAACGGTCACATCCCGGACCACCCCATC

TCGCACCGTCCGGTCGCGATCCGACGGCGTGGGAGCCGCCTCGAGATTCGCGCCTCCCGC

ACTCTTTTCCTCCCCTTGGGTTGCCCCTCCCGGCCTCTTCTTCCCTTCCCTTCCACCACA

CCGGAGAGGTGAGTGAGAGTGAGAGAGTGAGAGCAGAGACCACCACCACCGGAGAGGTTA

GTGAGAGAGGAGTGGTAATGGTGAGGCAACAAGAGTAGGTTCCATTTCATATCATCACTA

GGATAGCGTAGTTTGTAGGCTGCATCTCCATCTCCATCGCCATTGATTCGCATTGCATCC

ATCATTTTAGGATGTTCTACTAGGGTTCTTGATTTTTCTTTTGGTTTGTTGTTTTGACGA

ATGGAGGTATTGTTGGGATTCGCCGCCTGCTGCTCGTCGTCGTCGTCGCCGATGAGGAGG

CCGTGCGGGCTCTGCCCCGGCATGTCCGATCGTTCGTGATTTGTTTTTTCTACATGTTTT

AGGGCCCATTTGTTCTTGATCCTATTCTTTGATTCTTTTGTACTAAGCATTCTAAGGCGA

AGCCACCCATTCTTTCCTGCATATATACTTACAAACACATAGCCCCCATCTGATCTCACA

AACATTATTTCTCTCTCTTTTTTTCTCAGTTTTTTCTTTGTTGATTTACTGACCAAATTC

TTTGGAAGAACAACAAGATCATCTGGTTTTTATCTGCTCATTCTTTTGTACATCGAATCA

TATACATTTCCATTCCACCAAAGCCTTAGCCAGATACCACAGAGAGAGTGTGAGAGAAAT

CAGAGTGAGAAACAGAGGAGGAAGAAGAAGAAGAAGACGAGGAGGAGGAGGAGGAGGAGC

AGGAGGAGGAGGAGGTCTCTTCTTGGCACGTCGCGTTCCGGCGAGTGACGTGTCTCCGGG

>LOC_Os02g58390

TTGTTGTCATTTTCAAACTTATCAAATTTGGTAAAATTGTTAAAAAAAAGTGACTACATT

TAGTTTGCTGTCAAATTTTAGTAACTATATAAGAAGTTACCAAAATTTTGGTAATGCTAA

AATTTGGTAAAGTTGAAAATGGCATAAAGTGAACATGCCCTTAAGTAGAAGCTGATTCAT

TTATTCTTGTTGCACTTGCTCTAAGAGTAAGGATAATGGTAGAGCTCACTTCCTATTATT

AGCCCATCTTAAAGCCAATATATATAATAGATTAGCTATAAGGTTGGCTACAATATTTTC

TTCTCCTCTCTCTATCTCTTAATTATGCATTTAATGTATTTGTCTTGGATCTTGTGATAA

GCTAGCTCTTGCATAAGAGCCAACACCCTTGATTTTTCACTACCTCTCTCCTCCCTAAGT

TTATAGTAGGCTTATAGCTCACTATTATACTTGCTCTAAGGCCATTCCTAACCCAATGAT

TAGGATGGTGTCCATAACATTAAATAAGTTGCCACCTAGAATGAAAATTGATATGGCAAG

TGAATAAATGAGAAAATAGAAGGAAACCATATTTTGCATGAGACATGGTTTGTACACAAC

ATCCAAGACATCATATGAGATAAGTAGCATTAAATTTAAGTATGTAATAGTGATGTTTGT

ATTGGAAGAGTAGTGTCTAGTACTAGTTTCTTAATGATGTGGAGCTTATGGAAACTATGT

CTAATGTCTTGGATTGGAAATGGCCTAAATTGTTAAATCATACAAGTCATCGTGTTAGTA

AGTTTGTCGTAAAAAGATACTTCTGCAATTTCAAAATGTAGCTAACTAGTATAGGATGGG

ATATTACCTAGTGATATAAATCTGGACATCCCATCATGTACTAGGTATCTATATTTTGGG

ACGGAGGAAGTACTATTTTATAAGTAATATAGTTATAGTGTTAATTTTTTTTAGAAAATA

TAATTAAAAAGGAGATAGTACAGTAGTAAAGACACATAATAAACATAACAGTGTGTGTTG

GTTAGTAAATCGATGTCCTGTGTTCTGTTGTGGGACTTTTAGTAAACAGAGCAGCCAGGG

GAAGGGCCCAGGCCCAACTAACAATTAAAGGCCCAGTTACCTGCATCTTTTCCCTTCTTC

CCTAGTCTTCTCCTCTCCTCGGTGCTCAGTTCCTTCACGTGCTCCAGAGCAGCGTGGCCA

TGGCGACCGCAACGCGAGCTCCAGCATTTAAACCCGCACCCGTCCCCCGCTTCCCTTTGT

TAGTTAAGCTTTCCCCTCCCAATTAATTAAGTTTAGCTCTTATCAGAGCGAGAGAGAGAG

AGAGAGAGAGAGAGAGGGAAGAAGAAGAAGTGGAAGTGGTATGTATGTGCCCATGTCCAT

TGCCGCAGAAACAAACTGTAGCAGCAGCAGCAGCAGCTAGTGAAGTACTACAGTACAGTA

GTGAGGACTTAATCAAACGGGGCGCGCGGGGTGCCCCCTAGCTCTCCTCATTCCACCACC

>LOC_Os03g13010

ATAGCACCTTACTTTACTATTACGGGTGCTCTAGGTTCCCATACTCGCTGTAGACCAAAG

AGAACATGATGAGTGGAGTATACTGTGAAAACTGATAACAACTGTGCTGCCAGTCTGCTA

CTCGTACTGAAGTTTAGCATGACTGCTCCAGTGATTCCCATGGATTTGATTAGGAGCGGC

GATTGGCTAGGGTTTGAAACTTTGAAAACTGATCTGCGGCGATCAATGCGCGGTCAGAGC

TGCCCTCGGGAGAGCGGAACGGTCAAACGGGGGCCGGGGGCACCACCTCCGGTAGCGTAA

CGTGAGGGTCGCGTGCCACGAGGAGAGGCTGTTTGCTTCGGTGATGGATGGTGATCTCAC

CGCACGTGCGGTCCGCGTGCCGAGACCAATAGTACAGTGCACACATCACGGCATCACGCT

CGCTTCCGTCTGCGCTCCCTGCACTGCTCCCATTTTCCCAGAGGTAATATAGATTTAAAA

ATAAAAGCAGTGTAGTGTGAGTTTAATAAAAGTAAAATATGGAGGCGGCACCATTTGTAA

TGGGAATGAAAATCACATCAAGCCTAGCTAACGCACCATTCCCTGCTATAGATTCTACTC

CATCTTTTGTTGTTGTTGTTGCGCTTTGGCATAAACCAATTTCCTCGTAGTGGAGTAGCA

GCAGTGAAAAAGAAAAAAAAATCAAAAATCAAAATAATATAATGCCAGTCTGCATGAGCA

GAAATTACTCCATAAAAAACTGGGGAGAAAGGACCAGCTATGGGCCCACCTCACAGGCAG

CTTGTCAGTGAGTGTGGCAATGTCTGGTCCCCACTCTTCTAATCCAATCCCCTCACTAGT

CCTCCCTCCTCGGTCATCAGTCATCCCAACCCAAACCACCAAAATGAAAAGAAAACCCAA

ATAATTCCATCAGGATATTCTTGCTCACACATACACACACCCTCTCACAAGGTCACAGAC

TCATCGCCCTTGCAGCCTTCCTCTCTCTCCTCTCCACCCACTCTAGCTTGGCTTGGACCA

GATCTCACATTTTTTGTTCAGTTTCTCTCCACGCCACCACTCCCTCCTCCCTCTTCCTCT

TCCCCTTCCTCCTCACCTCCCATTCCCTCAGATCTTCCCCCATATCTCTCTGTCTCGCCG

CAGTCCGCCTCCCAATCTGAGGAGCTTTTCTTCAGCTCAAGCTTCTTGCTCGCTTGCTCT

CTACTATCTCTCCTTTTGACTCGTGGCCAAGCTGCAGAGGAAACCCAAAATTCCAAGAAA

AGCAACAGGTCAAGAAAGAGGGAAAGCTAAGGTGAGAGGAGATGGGAGGAAGGAAGGAAA

GAAAGATGGTGATGATGGCGATAAGTCGCCCCATGATTCGCGCCATCATTCCGTCTCGCC

CGAGGCCAGTGCGCTGGTAAAAGGGCCTCGCCGTCCGTCCGTCCGGCCGGCCGTGCGGCG

CGCGTGGAGGAGGTGGTGGTGGGTGCGGAGGTGCGTGCGTGAGAGGGGCGTCTTGCCACA

>LOC_Os04g49230

GTTTCTATCATGAATACGTACATAGACACAGAATGTCAAATCTTCTACCGTGGTCAACTC

TGATGTCTGTTTACATCAATATGCATATATATGATCTCCCTTCTCATCACTTACAGTATC

ACCCTTTTAATTTGGGCCAATAACACTAGTCGCAACTCGCAAATGCTATCAGCTATCCAG

CCGTTGTTTTTCCTCTCCGGGCTCTTATACGACCATCTCACCACCATACATGCGCACCGT

CAATGATCCTTCCGCCCTGTGGCTACGGTCAAGGGAGAAAGTGTGCTCCCCTGATGGACC

ACACGGGTGTTGCTCTCTGAAAGAACATTTCATATTTATATGCTACTGCAAATGATGGCG

CATCACTGGAGACATTTTTATAACCGCGAATCATGTGACCATCAGTGGCTAATACAGCTT

TGCATCACGTACTGGATAGGAGCGCGCGTGAATCCTAGGTTTTTACACAAGACCGAAATT

AATACAGACGATGGCAGAGGCAGAATTAGGACCAGCTGATACAATACTCCTAATATAGAT

TACTAATCCAATGTAAGTGCAAGTGCTAGCAATCCACAGTCACCTTCCAGGAATCTTCTA

GGGCAAGTCCCTCACACCCTCCCCCTCCACATCGCTTCGGCGCACTCTTCGCTTCCACTC

CCCATCTCCCACCGGTGGGCCGGAACGCGAGCGCAGGCAGGCGCAGAAGCAGCAGCAGCA

GCTTTCCTCACACAGCCTCACACAAGCCTACCCAAACAACAAGCAGAAAAAGGTAAAAAG

CAGCACCATCAATTCCCATTACCTAATTACCGGGCTGCAGTTACATGCCCTGCCCGGCCT

AGTAGCTTCCCCTCCTTTCTTCTCCCACCGCGCGCACCCCACCACGCCGATCGAAAACCC

AACCCCCAACCCCTCCCTCGCGCTCGCTCGCAGCAAGATCCAAACGCGCGCGCGCGAGGC

AGGCCGGTCAATGCCAGCGCGAGCCGCAGCAGCAGCAGCAGCAGCCCGCATCATCCCTCC

TCTCTGGCTGTGGCGCTGGATTGGCTAGATTAGATGAAATCGCCTCCTCCTCCCCGGCCG

GGCCTGGCGCGCTCGTGTCGTCTCCGAGCCGGTCAAAATTCGTTCTCGCATACGCGACGC

GAGAGGGCGCACACACGCAAACACGCGCGCGGCGCGGCGCGCGCAGGTAGGTAGATAGAT

AGATGAGATAGATAGGCTCGTACCATACCACGGCTTGGGAGATAGATCCCGTCCTCCTCT

CTCTCTCCGACTCTCCCTGTCTCCTCTCCTCCTATCTCTCTCTCGCTCGCTCGCTCGCTC

TCCCGGCCCCCTCCCGCTGCGCGCCGCGCCGGCGGGACGACCGCATCGCCATCACATCTC

CACGCCGCGTGCGACGGAGGCACGCTGGAAAAGGGGACGGGATCGAGGTGGACGCCGGAG

GCAGAGGCGGAGCGGAGGGCGCCGCCGCTAGGGGGAGGTAGTAGTATCTACTAGATATAT

>LOC_Os05g09520

GATTACCGGGTCAGGGACTTGTACCGTCTCTATGAAAATTGACTAACCACGTAAAAATCC

AATTCTGCCCTTCCACTAGTTCCATACACGCGAACAAAGGGGGAGGTACCCTGCACCGGA

TTCCTGTACCTCGCGGTACCGATGATTGTGGGTCGTTTGATCTGGCTGAATGGACGGTTA

CGATTGCAGTACCGCGTGGTACCAAAAATTCTGCTGGACAGTAAAAAATCTCTTTGATTT

GCTAATAAGCAGTATGAATAAACATATGCATAAGCGAAAGGCTAGGTGCTACTACTTCTA

CTGTGGAAGTGCTCATGGAGGGGGGCCGAATGGCCGATCGATCGAGAAAGCATTCATCCA

TTCCATTCTCTCGCTCTCTCAAAAGTTGCAATCTTTTTTTTTTCTCCTCTTCTTCTTTTT

CTTTTTCGTTTTCCAGCTCATCTCTGATGGATCATCTCTCTCTCCCCGTGTGGTAATTCC

GATGTGATCGATGACGCGTGCATGCGTCGGAGTAGGAGTACAGCCTCTGTTGTTCTTTTT

GGTCTTCTTCTTCTACTTCCTCCCAAAAATGCGTTGTGAGCGAGAAAAAGAGAAGCTTTT

TTTTGGTGTGCGTGAGTGTGCAACTCTCAATATTTGTTGCCCGAAATCTTTCGAGTTTGC

GTCTTTTTGGGCTTACACTGTCCCTTTTTTATCGCTGCGTCCAATCCTAATCCACTATTT

ATGCCTAATTAATTACTCCGTCTGTTCTTGAATATGACAACTTAACTTAAGTAGTGGATT

GAACCTTACGTGGTACTGTACTATATATCTAGACATACATTATATCTAGATATATTGTAC

CAGGTAATATCTCATATAGTACTAGGATGTTATATTCTCTGGTACTCTCTACCGGTAGTA

CAGTAGGAATGGTCTGAATTGTTCGTTATTAAGGGTAAATTATAAATTTACTAATATAAT

GATTACGGAATACTTTATTATTTATGTTTTTTTTGTCTAAATCAATTTATGGTCATCCAT

TTTATTGGCATCACTCATATTTTATCGAAAAGAAAAATAAAAGAGAAAGAAGATTTAGTC

ACTAATAAATGACGATTATACCTTACACCTATTTTTTTTACAATAATGCTCCCTCAAAAT

TTCTATAACCACTAACTGAAATGGTAATATAAATAGATCTAAACCACTAGATAAAAAAGA

TAAACTGTTCATATTGCTTTCTATCAGTAGCCTGGGATTGGGACGGAGGAAGCGAAGAGA

GAGAAAGAGAGAGGTGGTTTTGTTTTGTTTGTCCGGTAATGGCTGCCGCATTGGTGGTGG

TGGCCTCCTCTCCTCTTCTTTTATTTCGAACGCGACGCCACCCACGCGCCTCCCCCTCCC

CCCTGCGGTTTCCCTCTCTTATTCAAAACCTGTCTCGATTCTCACTCACTCTCACTCACT

CGGACTCCTCACCCGCTAGCTACCCCGGAGCGCGCCGCGCCACCGCTCGACAGCGGCGAG

>LOC_Os06g50060

TACATAATGTTATTCCTGAATCATCATACATCATGTACCATGACCTTCAATTCTAAATAC

TACCTCCGTTCCAAAATAAATACAGCCGTGGATATTTGTGCCCAACGTTTGACCGTCCGT

CTTATTTGAAAAATTTGTGAAAAAATTGAAAATATTTAGTCACACGTAAAATATTATTCA

CGTTTTATCATCTAATAGCAATAAAAATACTAATTATAAAAAATTTTCAAATAAGACGAA

CGGTCAAATGTTGAATGTGAATAGTGCATAACTGCACTTATTTTGGGACGGAGAGAGTAT

GTTGTACTGTGGTTCTGCAGTTCTTCTGCTGCAAAACCGCTGCCAACCTCTAAATAATTC

AGGGACCTTGATCTTTATTGCACAGAAAAAATTTGAAGCTAGCAAGCTGTTTCATCTGTC

ACAGCCTTGGGGTTTATAAATGAGTTCACATAATAAAAAGGACAATCATATATTGGTTTG

GATGATGCACTAGCCACATTATGCATAATGCCAAACTCATAATTCTCAATAGAATAATTG

AGTCATAAAGTTGTGGTTGTGCAACCAACTGAAACAGGAAAGCAGAGGAACGTGAGACGC

CATGGGAGATTGACACTGCCGCAGCGTGATGGAAGGTACGTAGTAGCAGTTGAGAACCTG

TACTGTATATTAATTGAGCAGTTGAATGATGTTAACGCACACGTAATCCATCTCTGCAAT

GTACTACATATTGATTGAGCAGTTGAGCTATAAGAATATATATATACAGTAGCGTAGCAT

GAGCTGCATATCCCTGATTTCATTGGATTAACTTTTGTGCTTCTGTAAGACTTGACAAAT

GGAGTATCTTTTAGTTGTATACATACGCTGCTCATACACTATTGATCCTATGAAAACTGA

CATAAGGCATGATTCATGAAGCACATTACAAGCACTAATTGATGGATATATGTATGTACG

CGAGTGCAAGTTCATGAAAGGAACGATCAATCTTGAAAGAGTAACAGCAGTGCTCCCTTT

GCTTGTGACACACTACTAGCTTAGCTATTAGCACGTACTCAGGTACTCTACAAATGAATG

ATGAAAGGGAGAGTACATATATACAGGAGCAAGAGAGCCACAACCTGTCTACCATTAGTA

ATTAGTAATTAACCCCCATGACTGACAGCCAACAGGAACTGATCTTTTCAATCTTGTTAA

GTTAATTGATTTGCCGCCTCCGATCGAATCCATTAAGAAATTAATGGAAAGACAATATAC

ATCCACAGTAGGAGCAAGATCAACCACTCTCCTCTACATCTCCAATCTCCATACGCCAGC

ATCTTAATTACTCTACAATAATCAATCAATATCAACGCATGCATACCGCATACGTGATCT

CAGAAGAAAGGTACAGCTCCATATAAGCACACAATACCTCGCTCAGCTGTGCATAGTTTG

CATATTTTGATCAATCAATCAAGCTAAGCAAGCTAGTGATTAGTCCATTGATAGATATCG

>LOC_Os04g56850

ACGAGACCGAGAAAATGTGGCGTGCGTCGCATGGTGGAGGGAGGGACAGAGATGTGGCAC

GGCTCGGCGCGTCGCGTTTGGCTGGACACGAGGACAGTACGTACGCCTGCGCTGACAAGT

GGGGGATCACCATGGGAAAGAGAAGCGAGCAAATGGGGATAGTGTCGTGCATTGAGAGAG

AGAGAGAGAGAGGCGATCGACTGACGACGACGACGCAAGGAAAAGCTACAGCGGGTAGCG

AGGGACGACACGCAGCGAGCGAGCCTGCGCAAATGAGGAATCATGGCTTCCCCCCTCGAA

AGGTCGGCATCTTTCGCGCAAAGCAGCCGCGGTCAGCAGCATCACACACTGCAGCAGCAG

CAGCAGCAGCAGCAGGAGAGAGGCTGGGAAGCCAAGACCAAGAGAGAGAAGGCAAAAGGC

GCAAAGGGGAAGAGGGGAGCACGACCACCCTCCTCCGTATCATTGCCATCTACTGCCCCC

ATCCTACTACTACTCCTACACCCGTCTACTCCACTCCACAACCTCTTCTTCTTCCTCCTC

GCGTGCGTCCGGGCCTAGCTGTAGCACGAGCTCGCCTGCCCATCTCTCTCTCTTCACCGC

TTTGCCTTTGCTGGCGTTGCGCGTTTTGCCACACTCGCTGCTTCTTTTTTATACTTCGGG

TTTGGGCGGGTGCCACTGGAGACATTCGTGGGGTGCCATTGCGGCGTCTTCTCCTCTCTC

AGGAGGTGAGTGGGCTCTGTGCTCTTGGCTTGTCTCTCAGCTACTTGCTTCTTCTTAGCA

TTACCCATTTTAAGTTTTTGGTTGTTCTAATCCGGTGATCTTGGGTGTTCCCTCCGGCAT

TGCCGATGTTCTTGAGACTACGATCACACATTCATATGCATGGGTGAGTGATGGTCTTCG

CTTCTTGTGAAAATGCCTCACCTGGGGGAAGGAGGGCTGGTACTAGTGCTACTAGAGGGG

TGGCGTTCCTGTTCCCTTGTTCGTAGGCATTCGCCTGGAGTTGTGTTTGCATTTACAAGC

TGCACGGCAGAGGATGATTGCCAAGTGGCATTTCGTCGAGCACGTAGCTGATATATTCGT

GGTGACATTTACTAGTACTAGTGCTACTACTCTCCGCGTAGCTTTCTACTGCTACTAATC

CTAGAGACATGAGCTGTGTGGCGTAAAAACACAAAAGCCGCCTGCTTTCTTGGGAAAGGG

AGGTGGTTTTGGCCGCCATGGGGACCCAACACCCAAGCTATAGCTAGCCAATGCATTTTG

AACCTCCCTTCTCCACGAATTCCTATTTTATTTTGGCTCTCCACTTTGACTGCTGCTCAC

ACTGCACACCGTATGAAATGATATCCTCTGCCTCTGTTGCAATGGTATGCAGGCATTTGT

GACATTTGGTCGATGGTGAAGTGTGGCTAAGCTGCTCGCTTGGCTTAGTACTCTGGAATT

GAGGTTTTATCAGAATCTTGGCATTCAGATTGCAGCTGTTCTTGGGTTTCTTGGAGACAT

>LOC_Os06g48950

GAACTTCATCCTGCAATTCTACCTCACAATGCGAAAATACAACATACTGTGTAACCATAA

GTTCTTTTGCAGTATAACCATATCTTCTTGTTTAAAAGAAATCAATCACTGTACTAGAAT

AACTACAGTCAAACCATAACCGTCCATTTGAGTACTCTAATATTTAGTGCATAATATGTC

AAAGAACAATTGACTATTACTGTTATATCTCCGAATTATCGGGGAGCACGTTTAACCAAG

GATAAATTTCTAAAAGTGTGCCACAACTTTAGAAGGAATCCAAATTTTGTACTCAAGGAA

AAGAGCTAAATTTTCAACGCGGAGAGACCATGTTCTTTCCTGCTTAGGGATGAATGGATG

AATTCGCTTTCGGATCTTGTAGACATGCAATTATTTCAAAAAGAATAATTCTTAGTGAAC

TATTTTAAGTTCAGTTTTGACCTTCAAAATAATAGTTACTCCTAATTCATGTACTTTTAC

CCTCAAATGTTTTGTGTTTGAGAGGAGAACCAATCCCTCCTCTGCACGCAAAACAGAGCG

GCCTTTTAATATTAGTTAATTTTTTTAAAAAATAGATCAATATAATTTTTTAAAACAACT

TTCGTATATATATTTTTTTTAAAAAATGCACCATTTAATAGTTTGAAAAGCGTACGCGTG

AAAACGATTAGTAAGGTGGGTTGGGAAAAGGGAGCGCCGAACTCAGCCGTAACAAGAAAC

AATATGAGAGTTAAGCCATTAACACTAATTGATTATTTAATAATTACTCCAAACTTAAAC

AATACTCTCAGTACGAGAGTATATTAATACAATTTACTCCCTTTGTCCTTAAAAAAATTC

AACCTAGTAGGATGTGTCATAATATAACGAATCTGGACAGAGGGTCTAGGTTGCATTTTT

TTACAGAGGGAATACTAAATAATGTTTTTTAAAATCTGATATTCATTGTCCTGAATTCAA

AACACTGTAAATTAATTTGGTGTGAGCCTAGCAGGAGAGGGTGTAGGACCCACACAAGGC

AGAGAGAACAAAATGGGAGCAGAAGTGAGAGAAGAAGAAGAGGATGGGTGTAGCTGTAGC

TGACCTCCTTCCACCTCTCGCACACGCACAATAACAAACCACTTTTAATTCCCCGAGGAG

AGCAGAGAGCTCGAAGCAAACAAGAATCACAGGAGCGAGCACGGCAGGCTGCAGCATCAC

CACCACCCTACTCTCTCTCTCTAGCTAGGGTTGCGCTGCTTCGGTCTCGGTCTTGGCGCC

GCCTGCCTCGCCCTTCTTGCTCGCCCCTGCTCCCCTGTCCCTCTCGTGTCGGGGCGACGG

ACGGACATCCTCACCCGCGCGCACGTACGGGTGATCGATCGATTGATTCCTGCTCCGACG

AAGACGGCGGCGGCGCCGGTGACCTCGCCGGACATGCGCGGGGCCCGCTGAGGTCTTTGT

TGCCGGCGAAAGGGGTGGAGTGATTGACTCGGTCGGTGTCTGCCTGTCTGGTCTGAGGTC

>LOC_Os03g49990

ATCTACATTTTCAAGGTGTTTAAGATGCAGCTCACGATTTGTTGCATCTCCTTCACCTCG

TGTCCACACGCTCTGGAGCTTCCCCATGCTCCTAGTCATACAAGATGCCTGTTCTCGGGC

ACAAACAGATGTTGCGTAGTCCACTTTGAGGGCCCATTTTTCTATTGCGCGATTTATCAC

CCTGTTTTGGTGCATCTGCTCCCTGGTCGAATCTCCGAGGGGGAATACTTGCTTCCCCTC

CTACGGAGTCTCACACTTCGACAAACAACTAAAAAATAAGTCAGAAAAAATAAGAAAAAA

TGTATGTGTACTTTCTATAGTATTATATCTACGTGTAAAATGACATTGAAAGAAAAAAAA

TTCTAAAAGATTTATTGTCTTAAAGTTGTAAGAATATTTTTACAGTTAAAATATAATGAA

TTTGATGTTAAAACTTTGCAAATAGGTCTAACACTATTGATAGTATATGAATATTTTTTT

CTAGATTTTTTTCGATATTTGTTAGTTAGTATGCATAGAGTGTGTACGTGAAATTTATAT

GCACAACATACGTTTCCTAGTATCAAATCAACTAAAGGTCATATCTACTCTTTACCTAGA

CTAATATAGTACTATGTAGTTTAAAATTTTATAACCAAACTTATCGTTGCATTTTTATCT

GTCAGTTTTGTAAGATGCATTGGCATAAGTTCATGAGAAACCATAAAATTTCCCTAACGA

TAAATAAAAAAAGAAACATTTAAGTATTATATCGGGCATAGTTATCTTACTCCTTTTCGA

TCATACTTTGGAAGTAGTTTTAAGCCCTTATGAGCTTGATAAAACATACTCCAGTAATTT

TAAATCGAACTTTTTTAATGAAATGTCTCCCAACTTAAAAAGGGTACAAAGAGATATCAG

GATGTAATAAGCCGCACCTTGGGCTACACAGATCGCAGGCACAGACTGGGAAAGAATTCG

GCTCGCCGTTGTCTTCCGAGATGGAGAAGTGAGCCGTAGGCGTAGAGCCGCATTTCTCAT

CACTCCTTTTTAATTCGCGGCGATTTTTGGGGTGGTGGGCCGAAGATTTGTAACTGGATG

GTGAGGTCTGTCTGGTGAAAAGGATGGCCGCGACGAATCCGTACATCGATCGATCCAGCG

CTGTGCGTGTGTGGGTGGTGTGTTGGAGAGAGAAAAGGGATGGGAGAGAAGGCACCGGTA

GGGCCCACCGCGCAGTGAGAGTCTATAGATAGATGCCTTCCTCTCTGATCACCTGATGCC

CTTCCTCTTCTCCCCCCTTGCTACTACTAGTTGCTTGCCTCTTCCCACCTCACCTCGCAT

TGCAATCTCGCATCGCCTCTTCCTTCTCTTCTTCCCCTTCTTCTCCCCTTCTCATCCAAC

CTCGCTTCCCAACCCTGGATCCAAATCCCAACCTATCCCAAAGCCGAAACCGAGGAGAGG

AAAAAGGTTACGCGCAATTATTACTAGCTATAGCTAGGTAGGTTTGGGGGAGGCGAGATC

>LOC_Os02g22130

TGCACACGTTTAAGCCTGCTAAACAATACTCAGATTATTTAGTACACTACACAACGGCAT

AGACTCGGCAAGCTCGGTCTACCTACTCAACGTCATTGCTTTAGTATTTTTACTCAAATT

ATTTAGTTATTATTAGTACAGTACAATAGTAGTGAATAGTCTAAAGACTCGGCATGGTCA

GTCTACTCTAACGTCATTGCTTAGTATTTTTGCTTAACATTGTTAACAAACACTTGGGAA

AAGGAGCTCTGCCCAACGGAAGGGAAGGTATCCCCATGTGCTTGTGTCATTGGAGTAGCA

CTGTGTTAATTATTAGTGTGGACGCTGATGATGATGTCCGATGTTGAGACAATTTTCGAT

CATCTAGAGACTACCAACTCTTTTTTCTTCTTTTCCTTCGGTGCTACGAAAACGAAATCA

AAGATGAATGTCTCCGTGACACATGGGCCCACTCTACCATCCGCAAGGGTTCCAAAATCC

AACCGCAAAAGTCATCTGGAATGTTATGTAGGCCGACGCAACAAAAGGAAAGGCGCGTCA

CGGCGTCAGACGAGACGACGACGGGGCTCGCTTTCTTCGAAAATCTGCAGCACCCGACGA

CGCGCATCCCTCTCACGACCACGAGGAGGAAACTACGGCCGCGAATGGCTCCGCTATCAG

TAGGCCGTTTCTACTTGCCAAACTGGGGTGTACTATGGGCATGTTTGGATTAAAGCCAAT

TTTTGCCCTACCAATTATTTAGTAGTTTTGAATAGTGCTTGTTAGCTATTTGGATTGAGG

CCAAATCTTACCAATTCAATAGAGTTAGAAACAACATAACCCAATCAAAATTCCAAAATT

TATCCAATATTTGACATTACCAAAATTTTGGTAGGGTTTCAAACCAAAACAACCCTATGT

CCCTGTGCTGTGACGGGAGAAATAGGGAACATAGGAGGGGAGCGTGCGGGATCCAGTCAG

CACCGCCGCCCTCCCGCCCGGTTGCTTCTCCCGTCCAAAACAAGCGGACGGCGACCCGAT

CAAACAAACCTTGTACCGGTAGCGCGTAAGCAAGGCCAAGTCGCACAAGGCCGGGTTTGG

ATTGTAGTGGAGTGGACTGTGCAGACCTACTCGACTATACTACTACTATATAACAGGAAG

AGGACCGAACGGAGAGGCGATAAGGAGTAACGAGTCCACTTTCTCAAGCGCTAATCTAAT

CTACTTGGGGCAGTGGTAACAGGGGGAGGCCAACGCGGGGGAAACAGCTGACGCGCTCCG

CTCCGTTCCGCTGCCGCCGTTGGCCGGCCGGGCGGTCGCTGTTGCTGACTTGACCAGAGA

CGAGCAGCAAGGGGGCAGTTGGATTGGATTAGAGGGTGCTTTTATATAGAGGAGCACGAG

GAGGAGGAGGAGGAGGGCCATCAAGCATATCCATCTCCGATTCACAGATTCTTTCTGGAG

ATCCATCATCCAGCGAGAGCGAGAGCGAGAGGGAGAGGAAGAGGGAAAGCCGCCACCGCC

>LOC_Os04g58750

TATTTTTGGAGCTCGCTTTCCCTTTTCCAGAAGCTGTGGGCGCTCGCGATCTTACTTTTT

ATTTGTCCCCCGAGTTTGCTCTATTTTCGCCTAGATATGGCCATTAATTCGTTAAATTGG

GTCGACATCTCCGCATTTGGAACGTTGTTAAACTGACTCCGGTAGCCATTAATCTGTTCC

ACCTATCCATTTGGTGGTGATATCTGCGTTATTATGGTCAAACTATTTCTGATTTATGTG

CGCCTGTTTACATATTATATAGCGTTTCTGCTAGTGATTTGGGGGTGCTGATTAGTTTTG

TTAGTTCTGCATCTTCGAGTGATTTTTGGCTGCAAGCTTGTGGTAATGGAGTAATTTGAG

CGGAATACATGTTTATCTGTAGCGGAGGAGGACCATGGGGAGTCGTATGAGCTGCCGGCC

TTTCAGGAATTCTCCTTCGAGCAGCTGAGGCTGGCCACCTCAGGCTTTGCGGTGGAGAAC

ATCGTGTCTGAGCATGGTGAGAAGGCGCCCAATGTGGTGTACAAGGGGAAGCTCGACGCG

CAGCGTCGCATCGCTGTGAAGAGGTTCAACCGTTCTGCATGGCCTGACCCGCGGCAGTTC

TTGGTATCCTATTGTCTTCACCTTAATCCTTTTGCATTGCTAACGATGTGCGTTTGCTTG

GTGCCAATGGTAGTTGGGTACGTGGTGGTTATTGTGGGAATGGTAGATTCTGTTCTATTT

ATAAGTGCATGAATTGGTTTCTAGGTTTGGGTTGTCTCTTATCATGGAGGTTGTTGATGT

GTACCTGTTCCTGAGATGATATGCATGCTTGTTCTTTACTTATTTGGCTTTTGGGTGGAA

TATGATGGCTTGCAGTTCAAATTGTGGCATCTGCCAACTGTTTGCACAACATATCTTTTT

TGGTATGATTATTAGATTCCAATACTAAACAAGTATCAGACAGCAATCATTACAGCTAGT

AGCACCATCATTTTGTGGATTACATGTTTGATTGCTACATCACTCCATTAATTGCTATGT

GTGAGTTAGTGTCTCAGTAATTATAAACTTACTATTACCTGTTGTTAACATTATGTATTT

TTGACTGCATTAGTTCTAGCTCTTCCCGTGGACAATGCTTTATATGCCTTGTTATTTTGC

TAACTGTCACATGTTAATTCACCACCATGCCATTTGTTTCTGATTGCTATTGTACATACT

ATTAAAATGGCAAGTATAGAGGTTTTCTTCCACAACTTTAAGCAGCCCATTTTCCATATT

GATGGCATTTTGTTGTGTGAGTGGATGGTGGAATCTACTTCCTATTTGTTGACGCAACTT

TTTGTTTGATTACCCATATATTTTTGTGGTAAATTCTCTAAGATCATCTAATAATCCTTT

TGGTGGAAACAACTATGCTTCAGGAAGAAGCTAAATCAGTTGGACAACTTCGGAGCAAAG

GTTAGCAATTTGCTTGGCTGTTGCTGCGAAGGTGACGAGAGATTGCTTGTTGCAGAATAC

>LOC_Os05g32270

CCAAATCTATAGCGTTGTGTGTGTAAACAAATAAATAAAAAAAAAAGTCCACCGAGACAA

GAAAAAACGCACCGTTGCCGTTCCGAGGAAACGACGGCGGGCAGGACAATCCCGCATCCG

CCGTGTCCCTTTCCGCCCCCGGCCCGGCCAATAGCCCAGTGGGCCCCACCCCCCCTCCCC

AATCCCATCGTATCCCCCCGCTTCCTCGGATTCCCCTGCCCCCTGTCCCCCCATGTGCCG

GCCCACTGGGCTCCACCCCCCCATCCATCCACGCATCCACCCCCCCCATCCGTCTCCATC

CCTCCCAGTCTCCCACCTCACGCACTCGATGCGATCCCCCCCATTTGAAATCCCCCCCCT

CTCCTCACCGCGACCGCCTCCCCCAAACGGTCACCCCCGCTTTGGCTCTCTCTTCTCTTC

TCGCCTCGCCTCGCCACCGACTCCGATCGAGTGGGGGGAGGGAGGGGGGGTTTGCTGCTG

CTGCGTGCGCGCGCCATGGCGTCCCCCGGCCCCGCCGCGGGGATGCAGCAGAAGCTGGAG

GCGGCTGCGGCGGCGGCGGGGGGAGGAGACGGGGCGGAGTGGGGCCGGGGAATGCAGAAG

ATGGAGGCGGTGGGAGCGGGGGGAGAGGGGGTGGGGGCGGGGGCGGAGCAGGTGGCGCCG

CCGCCGAGGAGGCCCGTGGCGGCGCGGAAGGAGAGGGTGTGCACGGCCAAGGAGCGTATC

AGCCGCATGCCGCCCTGCGCCGCCGGGAAGCGCAGCTCCATCTACCGCGGCGTCACCCGG

TACGGCTCCTCCCCCCTTTGCCCTCTCCCTCCTCCGTGTCTCGAGTCGCGGAGAAGTTTT

ACTTGGATGCTTATTAGGGGATATGCAATCGTTCTCTGCTTGGATTGATCTGTGGCGCTA

AATCCGTTCGTCAGGTCTCCTTGTATGCTCAAAATGGTACCGTCGATTCGGGATCTAGAT

TTTTCTTTTTGTTTACTTGGTTATATACGGATTAGCTGTATTCATCGGATTACCTGTTGT

GTCTCCAAGGGGACGATCTGAAATAATTAGGAGCTTCGCTTGATCTAAATAAATATAAAA

GCTAATGAATGATTTTCACTTGACCAGTACATCCAACATCATGCTTGATTTAAGTTGTGT

CAAACTGGGTTGACAGGGTTTAGCTCTAATCTGGGTAAGAAGCAAGTTTTGGACCCTTTT

CGGTCCAGACAAGTGTGGGGCTCAACTAAAAGATGAATTTTCAAACTATATGTGTATTTA

CATACTTAAATCTTTTATTTGGTGTTTAATCTGATCCAAAAGTAGTATTTGTTCTATATT

GTTATTCAACTGTTCTTGTACCACCATATGCTACTTCTTTCTTAATTTTGTTGTAATTTG

TAAGCAAGATTTTAACTTTCAGCTGTTATTATGTATGAATTTGATTTGACACTTGTTTTG

TATCATCTATAGGCATAGGTGGACAGGCCGATATGAAGCTCACCTCTGGGATAAAAGCAC

>LOC_Os03g44500

TTGTCCATAAAAGTTGACATGATCCCTTATTTACCCCTCATTTTCTTAATGACTTGTGGG

TTCAGGATTTGCTTGTAGTTTCTTTTTAGGATTTGTAGTTTTTTCCTTATTATTACTCCC

TATGTTTTTAAATGTACGTCATTGTGGATTTTTAATCTTGTTTTCTTTTCATGCATGTAC

AAGCAAACAAATTGTTACGTTGCACCACACGTCTATACAGTAACATCGTACTTCTCTTTC

TTTATTTAGTTTTACGCTCGCTCTTCTATTTTTTTTATTTCATGTATTATATTCGTGTGT

TTTGCTTGTCTCGTATTGCAAACACCCCAAAAACCTCTCTCTATGCAAATCCAATATATT

TTTGAGAAGACTGACCATCCACTCTACACATGCTTACTAACTTATCAATATGTTTCTAAG

TCAATTGTCGGGCTGGACTATTTATGGGCTTGCAACTAAGTGGTTTATGGACCGAAGCAT

CCAAGTATCCACCAAATAGTCTAGTGGATAATCCGCATTCGTAAAGTATCAGAGATTCTA

CATCTGTACCTCACTATCCGAATTCGATCATATATCCACCGAATTTCTGAAAACATCATA

CCATATCATCGTTTTGGAGTGGATCATATCAGCATTTCCGTACCTTCTCTCGGTAAACAA

CTAAACACAGAAAGGGATCATCTAACGTGAACTTCTCACTAAATATAGAAATGGATCATC

CGACGTGAACTTTCTAATACTAATATATGAGCCTCACCACTTACATGATCCAGGTATACG

TGATCACGGATCCCATACACTAAACACGTATCCGGATCCATAATAATAGGCGTTTGAACA

GCAGGTGGCAGCATCGGAGAGGAGAGTGGCATTTCGAAACGGGGAGGCCACGACGGTAAA

ACCGCTGCGAGTAAAAACCAAAAAAAGCCGTTCTTAATTGAATCACCATTTCCTCTCACA

CACTTCTCTCCTCCTCCTCCTGTCTGTCTCCTCCACCCCACCTCACCTCGCCGTCCTCCT

CCCTACGTTGGGGTCGCCATAGCCGGATTTGCGCGTTGGCCCCCCGACCCCTCCCGGAAT

TGCCAACTCCTCCTCCCCCTTCTTCTTCTCTCCTCTCTTCTGTCTTCCCTTCCCCCGCGC

CGCGCGCACGGCCCGGCCGGCCTCCCGAATCCCCACGCAATTCCCAAATCTCCCGGGGCC

AAAAGCTTTGCCGCCGCTCCACCACCACCCACCACTTCCTCCCTCCCCCCTCCCCTCCGC

CACGATCGCGCCCAGCCCGAGCCCCAAACCACCCGACGAATCCGCCCCCGCGGGATTCTC

TCCGCCGCCGCCGCCGACGAGGAGGACGCCGACGACGACGAGGAGGGGGAGGAGGCTAGG

GTTCCGGCGGAGAATTCGCGGCCGCGGGCGGCGCGCCGCGTAGGGGGGGCCACCCCCGCG

GCGCAGCTCCGGATCTCGAGGCCCCCGAACTCTAGGAGGGGTGGGGTGGGGGGCTCGGCG

>LOC_Os05g05240

CTATATAACACAAAACAATATATACTAAAATACATTCAATGTTATATTTATTGAAATCAA

AATGGTGTTGTATGTGTTGCTAAAATTTTTCTTTAAACTTGGTCAATTATAAAGACGTTT

GACTAGAAAAAAGTTAAAACATTATGAAACAGAGTGAGTACTAGTTTCTTAATAATTAAG

TGCCCACTTGGCATAGCTTCCGCTTTAAGATGGAGCTTATTTCGTTTCACTGATTTTTAA

AAATAAGTTTATAAAATATATATTTAATCGTCTTCGTCGTGATCATGAAAAAAAATTTGA

ACAATGGAGGCGGGAGAGAAAGCCACTAAAACCAAGCTTAAAATAGAAAATTAAATTATA

ATCCAATCATGACAGTTAATGCCGTGGTCCAAGTGTTCAACGGTAAAAATGTGTATTAAG

TCATGGTGCCACAACACTTGCTTAGAGCAATTAATAGCGTAGTTTTACATGTTTTTGAAT

CGTGGCACCAAAGCACTCGCTTAGGTCAATTAATAGCCTAGTTTTGCATGTTCCATGCAT

ACTGTACTGTTTTCTAATATCTTGTTGAGTCGTTTTATGGATCACTCACTTTCTCTATAT

AACTCAAATCAATTTTATCGGTTGACTACTAATAGACCATACACATACTTTTAAACTGAT

TCGTCAACCATATGACCACCCGCAAAAACCGATGACCATCAAACAACGACATTTCTCCTA

ATACCTCAAAGTCATCTTTGAAAACCCGCTTAATTTTGTCATCTCTAACCAAAAAAAAAA

GTTGACAAATGTCACGGTTGAAAGAAAAGAAAAAGAAGGTATTAGGACACGCACACAGCA

CACGGCACTGGAGGAAGCATCCTAGAATTTTTTTTTTCTAGAACGCCATGGCGTTCCCAC

ACACCTCCTCCTGTTGCTGTCCCCGGAGAAGAAACCCCTCCTGGTGTAATCTGAGCCGTG

TACGGTGTCCTACCAATAGTACTAGTAGTAATTCACACCAGTTAGAGCCCCCGGCCAACC

AGGACTCGTCTGCTCCGCTCATCTGCGCTGCTGGCTGCTGCGACGGCGCCCCCACCACCC

TCCGGCCGGCGGCGGCGGCCGCGCCTCCCATTTACTCCCGCCTCGTCCTCCTCGTCTCTC

TCTCTCTCCTCCGATGGTGGTGGTGGTGGTGGTGTCCTTCCCCCACCTCTGAGCTGTTGC

TGCCAGCTGCCACCATTGACTCCGGCAAAGCGTACACCCCATTTAACCCACCGTCGCTGA

CGCCTGGGCCCCACCGTATTGAATCCACCTCTAACACCTGGGCCCCACCAAATTCCCCAC

CCCCCTCTACTCTCCTCTCCTCTCCGCGAGCTCGAATTTCCAGTGCCGTGTGCTGTGTGC

GTGTGTGTGTCTCTCTCTCTTTGGTTACTACCGCCTCGGATCCCAAGCTGCTGCGGCGGC

GGTGGCGGCGGCGGGGAGCTAGGGTTTTGGAGGTGGGGGGAGGAGGAGGAGGAGGAGGGG

>LOC_Os12g42310

AAACCTATAATACTCGAACAAAATATGCAAATATGTATTGAAAATACAAAAGAATTTCAA

AACAAATTTTTATTAGCAGCATGATCATCATTTTTTTAGAGTGAATTACGCTTTGGACCA

CATTTTATTATCTAAGTTTCACTTTGGACCACCCTTAAACATATCTTTTCACTTTGGACC

GGATAAATTTGCCATTGTTGCGATTTGGACCACCACGAATGATTATTTTTTTAAGTACAA

TCAACGACCTTAAACACATTAGCTCTAGTATATGGCTGAACTCTTCTATGCGTAGTTCAT

ATGTTTGCCGAAAGAGAAGTTAGACATGACGAGAAAAGTTGTTCATAGTAATCCAAACCA

CAACAATGGTAAATTTACCCGGTCCAAATTGAAAATATAGGTTTAAGGGTGGTACAAATT

GAAATTTAGGTAATAAAAGGTGGACGAAAGTGCAATTTACTCGTTTTTTTTTAAAAAGAA

GGCATTATGTTCATCCAAAACATCCAAACATATATATATATATATATATATATATTATTT

TCTTGAACTTTTTAATTTTAAAAATAGGTCATTGCAATTTTTATTTCTAAATATGAACCT

TTGGGGGTCGCACCACCCCGCCCGACATGACGAAACAACATTGTCACGTCACTTGTGTTT

GGTGTGACAATTTTATTTTGCCACGCTAGTATGGATGGCAGGGCGACCAGAGTGGCATGG

CGAAATAATGCTGCCACGTCAATCTTATTGGCATGAAAATAAGTTTTAGGATGATTTAAT

TGTAAATTTAAAATTAAAATGGTCCAAAAAGTAAAAAAAGGAAAACCCAAACATATCGTC

ACTATCGCTAGTACCCACTCCCTCGTTGAGAAAGCCTTCATAAAATCCAAAAGAAAGGAA

AAAAAAAGAGACGAAGACGAAACGCAATAAGGTTAAACCCAAAGGGGCTACACGAAAACT

CCCACCAAACGAAGCCCCCAACTTGTGTGTGTCAGTTGGGTGCGTGTGTGTGCGCCCCAT

GCGACAGTGCGAAGTGAGCGAATAAAATTACAAAAATTCCCACACGCGCTGGCTGAGCGC

TGACGACGCGTCCCTCCTCCTTCTCGCTGTCCCCGACCATTTCGCAGTCCAGTCCCTGAC

CTCTCCCCTATTTCCACTCTCGCCACCACCACTCCGCGACAAGCTCGTGCTCCCCCCCAA

ATCCCAAATCTCCCAGGGGCCTCCTCGATCGATCCCATCCCCCCCCTCACTTCGCCGGCG

GCGGCTGCGGCGGCGTAAGCGGCGACGACCGGTGGTGTGGCGTGTTCTACCTAGGGTTTC

GGTGGGGGGAGGAGGGATGGCAGCGGCCTCGCCGCAAGTGGCAAGGAGTTGGGAGGAGTA

GGCGGAGCTAAGAGATTTTTTTGCTCTCTCGCTTCTTCTCTGTGTGTGTGTTGCGTGGTG

GGGAGGAAGATCTCGAGCGGTTGGAAACCCTAGTGGGGAGGGAGGGAGTGGGCGGGGGGG

>LOC_Os03g45420

ATTTAAAGGCATCTCTTCCAGTTTTATAATTAAAGTTTCACTCTTACTCATCTATTGATT

TGGCTAGAATGGACTTGTTTCCTTCGGTTTCGATAAGTACTGTTTGATGGCAATCATGGC

TAACTAAAAGAGCTATAGCTCATGATGATTGCTAACTAAAAGAGCTATAGCTCATGATGA

TTATATGAACATCATCTTACAATGTTTGCCTATCACTAGCAGAAGCGGCAAATTGGGGCG

TTGATTGATCTGCAGCTGGATGTGGAGCTGATGAAGGAGAAGGATGACAACATTGTCCCC

GTCATCAACTCAGACGTTCCTGTGCTCGAGACCTGTGACAGCGCTGCTGTTGGCAGCAAG

TACGAAGTTGTCAGACATGCTGGCGTTCGATGGCTAGTGGATCGATGCTGGAGCAGATCA

ACATGCGGCTGGTGATGGTTATCTTTTTATGGACAGTGGCCATGGAGGCATCCCTCGGCG

ATAGCCTCCTCGCGTAGGTTGCCAATGGTAACAGACTGGCATGGTTCACGGCCACCACTA

TCGTGTTGTCTTTGTGTCGTTGTTGCGGCCACCGTCATTGTGCTTTCCTTAGCGTCACTG

GTGACACTAATTTGTGACAAGAGCGTGGAAGTCAAGAGCGATGACGTGATGAGCACCGGT

GTCGAGCTCTAGAATGGGAAACCCCGGCGCGCTCGCCTTCATCAAAGCATGCGGATTGTT

GTGGTGCTGCTAGACGATGCGGGCCAACCACATGGACCCACATTTACCCAAGTGGACTAG

TTAAAGTGGGTTTGGTGGGTTAGTGGAGAGGGGTAACCCAACAGGTTAGCGGCAAACATG

TCCCACTTTAGGTTAAATATCTCCTAAACCAGGGCATTTAACTTTTTGTCACTTTTAATA

TGTGGCAATTAAGAATTTACCACTCACAATCTATAACACGTGGATCCTCATGTGTCTATG

ATATATGGGTTTAATAACAAATTCTTAATTAACACTCGTAAGATTGACAAATAGTTGAAT

ATCTCCTTAACCAGTAGTACCAAGTACCAACCATCGAGGCACCCAGGAATTGTGTGTTGT

ATGCAAGTATGACATCCACAAGTAACATTAACCGCTCAACCACGCCATATATATTACCCT

TATCATTACCATGGCGGGAAAGAAAAGAACGTACGGAAGAACTCCCTCTGGTTGACGCAC

GCGGGTGAGGCTTGACTGATTAAAAAGGAGACAGAATCCTCCCTGGGTTGGGTTTGTTCT

GGCTCGCACACAGCTCAGCAGCTAACTAGTCCAATGGAGCAGCTAGCAGGTGAGAAAATA

AAAGCCAAGAACAAGTCGACGTGGCCGCCTTTCGTTACCCACCAACCCAACCTCGTTCTA

CGGTACGCCACGCCTCCCCTCCCCTCCTCCGCCGCCGCCGCCTCAATTAGACCGAAGCTC

TAAACTCCGGCGAGCCCTCGCCGCCGCCTGCGTGGAGGGGAGGTCGGGAGGAGGGGGTTG

>LOC_Os01g12690

AAGCAGCCGTCCGTCCTCGAGTGGGGCCCAGCGCCGCCCCCCGCCGACACGGGGGACACC

CTTGTCATTCCCTCTTAAATGAATGGAGACTTGTGGCAGTCCTTCTCACGTGGAGGGGGC

TTTAGATGATGATTCGATGGGACGGGGCATACCTACCCCTACTCCGCCGGGTACAGGGTG

GAACGTGGGGGTATCACCGCCCGTCCAATCAGACGTGACCGGCGACAGGCCGGTCACAGT

TTGGTCCCGCCTGATTGACGTGGGTCAATCATGCAGTGCCGCACGTACGCCCTTGTCGCA

TTAGATGCAGTGAGGGACGGTTGGGGCGCCGCGCATTGATGGCAGCTCAGCCTGAGTCTC

CGTCCCACTCGCTCCCCTCGCCACGTAGCGGTTGGGCGAGGGCCTCGGGGCGAGGCAGCG

CGAGGGCCCGAGGGGCGTGACGTAGCACCCCGAGGCCCCTTGGTTGCCTCTGCGTCGCCC

GAGATGTGGGGGGAGAGCCAGGCTGTAGGGCCGCGTGGCTTGATGGGAAGGTGACTCCCC

CATACTTAGCATACCCCCGGGCCCATATCACCGACATCTGAAAAAATAATTGTGCCTTAT

AATCCCTCCGTCCCAAAATAAGTGTAGTTTTGTACTATTCACGTTCAACGTTACGTCTTA

TTTGAAAATTTTTATGATTAGTATTTTTATTGTTGTTAGATGATAAAACATGAATAGTAC

TTTATGTGTAACTAAATATTTTTAATTTTTTCACAAAATTTTTAAATAAGACGGACGGTC

AAACGTTGGGTATGGATATCCACGGCTGCACTTATTTTAGAACGGAGATAGTATTATAAA

ATGGAGGGAGTAGCCAGCTTCTGATAATTGAAGAAAGCTCGATTGGTTTCTTAGTTTCTA

GATTTTAATTCATTTTCTAGATTCTATAACCACATCTTTTTAGAATTTGAACGAAAGTTA

AAACTGAACTATTTACAAAATCTTATGATTTTGAAAGAAACTGCAGCTAACATAAACTCT

CCTAAGAGCAAGTTTAATAATATAGCCCACTACTAGCTCCAAAATTTAAATCATCATCTA

TAGTCAATCTAATAGCTCACTCATATAATAGTTAGCTGTAGAAATATACTGCATCATTAA

CATGGTTTATTGGACCTCGTGTTACAGCTAGCTATAAATCCGTAGCCCGTTTTTGCTATC

TCTCCTCCACACAAACATAAATCTGATGTGGTAACTTGTATAGCCCGTTTATGTCATCTT

ATTGTACCCGCTCCAAACGCGTACTCAATGGTTGAATGTCACCTAGCTAGCACGTGACCC

GCCTTTTCCCCGCACGTCCCCTTGCCTGCCTGCGGCCCAGCCAGTGCGCGTACGCGTACA

CACCTCCCCCTCCCCGTTTCAAAATCGCGGCGTCTCCCGCTATATATAACGCCACTGCCT

CGCCGCCGCTGCTACCACCTCTCGCCGTGCGACTGGCTGAGCTACTGGTGGTAGCTGCCA

>LOC_Os01g64430

GAGGAATAGAAAAAACACAGGATTCCGACAGAAATTGAAGTGTAAAACAGAGGATTACAA

AACACAGAAAAAACACAGGAATGGTCGTTTGATTGGAGCACAGGAAAAACGCAGGAATCG

AATGAGAGAGATAGACTCAAAGGAAATTTTCCAAGAGGTTGGAGCTCTTGCTAAATTTCC

TCTAAAATCCACATTCAATGTGCCATTCCATAGGAATTTCATAGGATTTGGAAAGCTTCA

ATCCTTTGAATAAAAGGGCCAAATAGGAATATTTTCCTATAGGATTTGAATCCTATGAAA

TTCCTACATAGATCTTTTGATTCAAAGGGGCCCTTAGAGTTTTTACCAGTCTCTCTCTCT

CTCTCTCTCTCTCTCTCTCTCTCTCCTTCCTGATTATCCTACTTAATTTTTGCAAAAGAA

CTACTACTGTAATTCTGGTTTTGTCCTGTAGCAGCACCATTACATTTACTAGTAGTTTTT

ATTCAGTGAGTGCAAAGAAGGAATCGATGAATACTCCTATATAACAGCCAAATGCGGCTA

AAGATTATTTTACATAGATTAAACACCATTAGAACCATAGGCTTATTTTAAAGGAACGTT

TTTTAAGCCAAGTGGTAAGATAGAAAACAACCAAAAGATTTGCTTCAACAGAGAAAGCTA

GCATGAGCTGAGCCAGCAGCATGGCCAGCTTGTCCTCACATGAACAGTTAGTCAACCCCA

ACAGGGGACTCCCGTCCTATTACGAACCGTGGCAAAGAAAAAAAATATACTCCATCCACA

CACCACACCATCTCAATATATCATCAGTCATCTTCTCCCTCTCCCCTACGGTGTACACTC

CAGCTCAGAATTTACTGCACAAAAATTAACCACCTCGTGCAGTCACACCAGCCATATATG

CATGCATGCTATGCTTACAGGGCTTACATCTGCAACTCTCCCCTCTGACATGATCCCCCC

AGGGATCCAATCTGAACATCTTTGCACCCATGCATGAAGCCATGAAATGCCATGAAAACC

ATTCACCCATACACCCCCCACACACACATACTCCGTATAACTAGTAGCAGTGTGTAGCAC

TGAGCCGCACTGGACTGTGCTGTACATGTTGTTCACATATCTTTCTCAGGTTTCTGCTTT

CCTCTTGCACTGTCCGAGCTTATTTTACCCACACACTTGCCCTTACACCTCTAATTATCC

GCTTCACATGCTCGCTCTCTTCTCCCTTCTCACTGTTCATGTCGCTCTATCTTTACTCGC

CATTTAATCGTTGCCTCGCGCTCTCACATGGCGCCCCATTAATATATAGCACCACTAGCT

AGCTCCTCTTCCAGCTCCAAGCTCCAAGAACTCAACTCACTCTCTCCGAGCTTAGCTCTC

ACTGTCTCGCCGCGCACACGTCGCTCTCACTGGCTTAGCTGAGCTGAGCTGCATTGGCTT

CTGCAGTGAGTGCAGGAGTGCAAGGCTATTGAGCTTAGAGGTACACCAGTGGCATTGTCC

>LOC_Os06g12210

CTCTCGAAATCAAACCCAACCCTAGAAATTTGGATTTCTTTAAATAATCTAATCCAACAG

TGTCAGAAAACCAAAAAAAAAAACTGGAATCCCCACTATATCAAAAATCTCGCTCTTCAC

AAATACAGCACGGCACTCGTTCCCAATCGGTGGAGGCCGCACTTGATCGTGTGGAGCGGT

GGGTAACTGATCGCACGGAGGGGGAAGGAACGATTTTTTACAGGTGGCCATTTTTTTTAC

GGGCTCAGATTTGATAACTCATATAGAGAGAAACATATTTTCATGTCTTAAATCCTAATT

ATAGAATCCTAAGAAGAAATTTAGGGGATTAAATTTTGGGTAACTCTTGACAATGCTCTA

AGAATACATCCACGATATAACTAAAAATAATTTCAGGGCAAAACATTATACTTTTAGTAA

AAAAAAAAATCTAAAATGAGTCTTAACAATGGAAGCCGAAAAAAAAGTTATACTTCAATG

TCAGCTATGATTTACTACCTTCGTTCTCATATAGATATTGTCCTAAAATACATATAGGCA

CATATCGAAACCTTAAATAATTAATTAAAATAAAAATTAAGATTTGACATGAGAAAATGA

TATACATAACAAAAATTTTAATGTGGTTATATTTTTAATAGATTTTATAAACATATAGTA

AGAAAACATAGAACTTAAATATTTGCAAAACATAGAACTTAAATATTTGCATTGGAGGCT

ACATTGATGTCCAAAATGACTAGTAAAATGAAGGGAGGTAGATTAGGCTTTGTTCTATAC

AACATTTTCCCAACTTCTCCTTCCTCATTTTCCACATGTACACTTACGGAATTACTAAAC

GGTATGTTTTTTTAAAAGAAATTCTATAGGAAAGTTGATTTTAAAAATCATATTATCTAT

TTTTTTAAAAAAATTTTACTAATACTTAATTAATCATATGTTAATCTACTTTTTCGTTTT

TTTACTAATACTTAATTAATCATACCAACCTCCCGAAACTGAAATAGATAAAAAAAAAGA

AAAAAAATATCCTATTTGGCCATAGGCCATAGCGGCTAAACCAGGTGGGCCATGGGGGCG

CATCCAGTGTGATTGTGTGAAGAGCCAGAGAGGAACATGTGGGCCCGGCCCCCCATCTCT

CCTTTCTTCGCAACAGGCAGGCAGCAAGCTGCAAGCTTGATCTGGCACGGGTGTCTTTGC

TCATCACATCGTCGATCGTCTTAGTTAATCATTTTTCTTAGCTAGCTCGATCTCGCCTCG

CCTCCTCCGGGCCTCCAGCGAAAGTAATCAATCAGATTCAGATCGATCAGCAGCAGCAGC

AGCAGCAGCAGCATCCGCTCCCATACATGTCGATCTCTCACTATAAATAACCTGCCCCCT

CTCCCTTTCTGCTTCAATTCCTACCTCACACTTCTGCAACAAGCTTTAGCTCCAGCCACC

GGAACGAGAGATCGATACAGCTCGATCAGCTAGCCGCATTCGCCCTCGCCGCCGCCGACG

>LOC_Os05g27730

TATATGATGATCCAGTCTTTCAAAAGCGCTTATAGGGTAGGGTGTACGCGTGTTATGAGC

GTCTATACTTGTTTTTCAACTAAATATATATGTAACAATATAAATATTCCTGCACTAATT

CCCATCATTTCAATGATTTAACATCCAATCAAATGCTAGAAAGGAAATGTAAGCAATTCA

AAATTCAAAAATTGACAACTGAAGTAACAAAAATAATCTTTTGATCCAACCTTACTACTT

AGGGGCTGTTTAATTCCCAAAACAAAAACTTTTCACCCATCACATCGAATGTTTGGACAC

ATGCATGGAGTATTAATGTGAAAAAAAAATCAATTACACAGTTTGCATGTAAATTGCGAG

ACGAATCTTTTAAACCTAATTGCGCCAAGATTTACCAATATGGTGCTACAGTAAACATTT

GCTAATGACAGATTAATTAGACTTAACAAATTTGTTTTGCAGTTTCCTAGCGAAATCTGT

AATTTGTTTTGTTATTAGACTACGTTTAATACAAATGTGTATCCGTATATCTAATGTAAC

TCGCAAGGGCAAAAAAATTTTGCCAACTAAACAAACCCTTAAGAAGAACCTATAAATTGT

TATACTTTAAAACAAAGAAAATAATAGATAGATATTATATGAGAGTGTGGTGCGCATAAG

TAGAAGTGTGGAAGATTGTTCTATATCTTCCGTGTAATGGAAAATACATCCAGTAATTAT

AGTTTTTGATTTTAAAATTTCTCCTGATTTCTTCTCGAATAATTGCTCCGATACCTATCG

CACAACAATTCCAAAGATGTGCTGTTGTTGAAGCACTGTATATATACAGCGTAATGAACG

GGAAGTGTTAACCATTTTATTAATACAAGGATATTTCATATGAAAAACCGAACAATAAAA

AGGAAAAAGCAAAAAAAAATCACATTTTTGTCTTTGTCCCCCCCAAATCCCCATCCCCAA

TCCGTCCGTCCATTCGAAGCGACACGCCCACTAGTCCGCCCTCAACCCAAACCCAAACCC

CAAGCTACACGTACGAGTTGAAACTCGAAAGCCGTGGGAACACGCGACGCTCTCGGGTGG

GCCCCACACCCCGGCGTCTCCTTCCCCGAAACCACACGTCGTCGCCTCCCTCCCTCTCCT

CTCACCTCCCGGCGCGAGCCTCAGCCGCCTCGTCTGACCGGTCAAACCGCACGAGCTTTG

ACCTCACCCCCTTCGCCGAAGCATCCCCAAACCATCACGTTCACATTAGCCTCCCTCTCC

CACTGACACCCGGACCCCAACCTACTCCGCCCCACCTGTCAGAGACACGTGCCTCGCCTT

TACGCTTCTGCCCCCACTGACCGGAGCAGCCGCTACTTAAGCCGCCGCGACACGAGCCTA

TCCACCACTCCCGTCTCGTCGTCTCGTTCTCGTCTCCGATCACTCTCCTCCTCATCTTCG

TCACGGTCTCCTCGCTTCGCTAGCTCGCTTGCTTGCTGGCTGAGCTGTGGTACGCTCGCC

>LOC_Os03g08754

AGCAACCTAGAGCCACATCTCCTTCTAGTAGTACAACGAATCTGAACAACCCTCCTAGAT

CCACATCTCCTCCTAAATTGTCTTTTTTTTTGGACGAATCAGGGAGTACTCCATATGTCA

AAAAAGAATTCAACTCTACGGATGAATCTGAACATATAGATTTTCTTTTAATAGAAAGAG

TATAATTACTCCATCTGTAAAAAAAAAAACCACTAACTTAGTTAAACCTATTTGATGTGA

TTGATTTTTTTTAACGAAGTGAGGAGGGTGTTTGGGAAACAGAGACTTATTCCAAGTCTC

TGTCACATCGAATGTTTGGATACTAATTTAGAGTATTAAACATACTGTAATTATAAAACC

CATTCCATAACCTTGGACTAATTCGCGAGACGAATCTTTTGAGTCTAATTACGCCATGAT

TTGACAATGTGATGCTACAGTAAACTTTTCATAATTATGGATTAATTAGGCTTAAAAAAT

TCGTCTCGCATATTAGCTCTTATTTATGAAATTAGTTTTTTTTATTAGTCTATATTTAAT

ACTCTAAATTAGCGTCAAACATCCGATATGACATGGACTAAAAAGTTTTAGCCTCACCTA

AACACCCCTTAATTCTTAAAATTTTAAGCTCTTCGAAATAGTTCCGAAGGCTCTTGAACC

CAGAACTATGTGACTTTTCAAAATGTCAAAAGTTGCAGGGGCAATGGCGTGGAGGGAGGG

TACACTAGTCCCTGCGTCCCCAGGGGATGTTTAGATATACAGTTGTAAAGTTTTAGCGTG

TCACATCGGATATTGTATTATGGTGTTGTATGGGTTGTTCGGGCTCTAATAAAGAAACTA

ATTACATAATCCGTCAATAAACCGCGAGATGAATTTATTAAACCTAATTAATCCATCATT

AGCAAATGTTTACTGTAGTATCACATTATCAAATCATAGAGCAATTAGGCTTAAAATTTT

CGTTTCACAAATTAGTCGTAATATGTACAATTAAATTTTTTTCTAAACCTATATTTAAAA

CTTTATAGAGATGTTCAAACGTTCGATATGATATGGTGAAAATTTTTAAGTTGAGATCTA

GACGGGGCCTGAGTCCTAACCACCTCGTGGCGGACACCTGCCTCCGTCCCTGTGGCTCCA

CCACTTGAAAGCCTTTGCCATAAAAAGTAGGAGTCGTCCTACTCCTACCCCTACCCCTAC

CCCTGCTGCAGTGCTATAGGCTAGCCCTACCTCCCTGCTCCTGTTTCCTCTCTTTCTCTC

TCGCCTCCTTCGCTTCGCTTCCATTGGTGGTGGTCTCCATTGCTCCCTTCCTCCCTGCCT

TTTTCTTCCATAAGTAGTAGTAGTAGCGGGAGTGGCCGCCGCCGCAGCCATAGATCTGCG

GCGCCGCGTATCTCGCGGCTGGTTTGGCTGGTGCGGTTAGCGGCTAGGGTTTTGGTTGTT

TCGTCTGATCGATCCTCCCCGATTTTGCGGCGGCGGTGGTGAATTGGTGTGTGATCTGAT

>LOC_Os02g52340

TAGGAATCTTTAGAAGAAAATAGGTGCACTATTACAAAATTATCACATACTACTAGTGAA

TTAAACAACATTAATCAACCTAATAATGTGCTTTAGTTGCTTGATGGCATGATTTGCCTC

GTTCCCACGCGAAGGGAGAGAAAAAAGAGGTCAGCATGGATGTTTTTCTTCCTATGATCT

TCCAGTGATATGGCTAGCGTAGGAAATTTCTCTATGTATTTCCTACGAACATTTTCATGT

GATCCAAATGACGCCACAGTAAAGCAATCCATAGGAACCCAAATCCTTCATTTTTCCTGT

GTTTCTCCTCTATTCTGAACAAGCCCTAAAGAAATTCAAGGAGGCACCTGAACGCCGGCC

AATTAGTGCCAGCGCAAACAGCCGATCGCTGAGAAAATGTGAATAAGGCAAAGCGCGAAC

TCTGGAAAGCGCTCGAGAGAGGTCGGCCGGCGACCCAACAACACACCACCACCGGCCTCG

CGCCCGCGGCGCACACAAGATCGCCACAGCGCCGTGGTCGGAACAGCGAATCCGGCCGCC

TGCAGGCACAGGCGGCAGTGCCACCTCCCCACCCCCCCAATCCGCTGCGTTTCCCTCTGG

CCTCGTCCCTTCCCAACAAACAAACCCGCCACCACACGGCGCCCAGCGCCCGTGCTCTCC

TCTCCTCTCCTCTACCCGCCCAGCCCAGCCGCCCATCCCCCCTCCTTTCCGCGGTCCTTT

TGCTCGGTTCAGCACGCACGCGGGCACGCGGCACGCCATCACCGCGCGGCCGGCCGCCTG

CTGCTGCTACAGCCAGTGGAGTACTGTTCTGGGCCGGCCGCGCCGCCCGAAGCCACCACC

CTTTCCTTTTCCGGAAGCCTCCCCAATCCTCCGCCATTCCCCGCATAGCGCCCCATGCCC

CTACCGACCATAGCCTCCCAATCCCCCATCGCCTAGCTGCTTCGCCGTTTATTTTAAGAT

TTTTCTTTGGGTTGTGAGTAAGGCTTGGTGGGGCCCAGCTCACGGTTCCTTCTGGCATAA

ACAATGACTTGGGCCTGCCCGCCCCGTGGCCTCGCCATCCGACACTGTGCCTCTCATCCC

GCGATTAGTATATTTTTGCTGTTTTTTTATGGGTGAAAAACTGTGTGGGCTCTCTCGTTG

GGTTGCTATCCGTCCACCCGTCCATTCATCCTCGCCTTTGCCAGTTGCGGTGTTGCCCCC

CTCGCTTTATTTATGTCGACCTGCCAATAGGAGCAAGTCCCCGTCTCGAGACACCAGCCG

CGCCGCGCCTCGCCTCGCCTCGCCTCACCTCACCTCACCTCACCCTCCGCCTCGTCTCCT

TCCCCCCTTTCCTCTACCCACGCACGCCCTCTCTCCCATCCCAGCCGTAGCCGCGCCGTG

ACTTGTCTCGATCGGCGGCGAGGAGGAGGGCTTCCGTTCCCTGGCCGGTGGGGGAGGATC

AGGCGGGGGGGCGGCGGGGAGGGGGAGGAGATCGGTCTCGGTCGATCCGCGCGCGGCGGC

>LOC_Os06g11330

GAGGAGAACGGTAAAGCCGGTCACAATCTTATCCCTGTTTCCATCGTACGTAGAGCCACA

TCCACAGCCACACGGCCCGCGTCGGAACAGGGAATCCCCCCGGCGTGCAGCTCTTCTTTT

CCCCGAGCAAGTTTAATAGCAAAGCCCACTACTAGCTTCAAATCATTTATAGCCAATGTA

ATATCTAATTCATACACTAGTTACTTACTATACTAGTAATATCTGGTCTTACCTGTCATA

CACACATTATGTCTTTTAAATCCGTGTTGTAGCTGGCTACAGATCTGTAGCCCGCTGCTT

TTCTCTCTCTTCCTTTATCTCTTTAAAATATGTTTATAGCTGGCTTATAGCCTGCTATTT

GTACCTGCTCTAAAACCGAATACATATTTCCTCCCTCCCAAAATATAGCTATTTTTAAGG

TTAGTGTGGGTATTAAGAAAAAGTAGGTGAGAATGATCGGACTGGATGAAGTGTGTCATT

GGTTGAAAAGAGAAAGTAGGTGAATAAGAATGATTGTGATTGGTTAAGAGGAGGTAAGTA

GAGAAATAGCTTCATTTTAGAACATTGTGCTAGAAATAGTTAAATTTTGAGATGGAGGCA

GTATCTATCTATTTATTGCGTATAAACTAAATAATTAGAAAAAAAATAATTAATAAGATA

AATTAATTTGTAATATATTCATTTACAAGCATTCAACTTCAAATTCAACTTATATTTTTA

TATAAGTTGTAAAAAATAACAAATTAACTCTAATCAGTATACATATATTTACAACTAAAT

TTATTTTTTACAACTTGCAAAAGTTAAATTTAATCTCAAATGCTTCTAGTGTGATATATT

TCATATTAATCTATCTTAACATTTACCAGTTGATTTGTGATAAACGGTTAGCAACAATTA

TCCAGGTGATTAGAATCCTTCTCTCCCCTTTGCAGCACTGTTCCCCCCACTCCCCCTTTC

CTTATCAGGAAGCCACACGCACTCTCCGTCTCCGTAGACTGCACGCTTCGTCGTCACATT

TTTCACGGCCTCCTCACCGCCCTCCCCCCTCGGGCCCCACGTGTCAGCCGCCCCGTGCGG

ATTCTTTCTTTCTCTTCTTTTTTTAACCTTTTTTTTTTTCTTTTTGGTATAAACAAGGAC

CAGTGTAGTTGCGCCCAATCGGAGGGGAGAGAAGAGGAGACACACGCACCGCACGGTGGT

GGTGGCTCTCGGCTCGGCCAGATTTGTTTAATTATTCGCCCCGGGTTTACCGCGGCCGCC

AATCGGAGGCGCCGCGCGCGGCCACATCCCTTTCTTTCTCTTTCCTCGCCTCGCCTCGCC

TCGCCATTGGCGAGCCGCGGCTATATACTAGGCGCACCCCGCCGCTGCGGCGCGCGTTTC

TCTCTCTCCTTGTCTCTCTGTGCTGTTGTTGTTGCGGGTGAATCTGCTTGGATTTCGTCG

GAGTTTTCTGTCGCGGAGGAGGAGGGGGTGGTGGTTGATCGGAGGGGAGGGGGGAGGGAG

>LOC_Os06g15620

GAGTCCATGTGCCGCAAGAGCTGTGGTGAATACAAACCTGGAACGATCCATCATCCATGT

GCGGTGCCCGCAAGCTGTGGATACGAACCGCGCATGTGCGGTGCCCTCTCCAGGAGTAGA

ATGGAGTACCAGAACTAGTAGCTAGTGGAGGTGCACTGGAAAAAGGGCATCAGCATGTGC

CGCCGATCTAGTGGACGTAAACTACGGAGTACTATTACTTATAGTATATGACCCACTCAC

CGATTTAATCTAGACTTGGAGTAGCATCTCACCACCTCGTCGTGTTATTTTTTTGTTAAG

AACAGTTACAATAGCAGACTATTAGCCAGCTATAAACATATTTTAATGAGATAAAATATG

AGAGAAGAGTAGTGGGCTACAGACCTGTAATCAGCTGTAGCACGGACTCTAAAACGCAAT

GTGTATATGACAGATGGGATCATATATTAATAAAATAATAAACAACTATTGTATGATTTA

TCTATTAAATTGACTATAGATAAATTGTAGTTAGTAGTAGGCTATAATATTAAACTTGCT

CTTACATCTCCTCGGGTTCGTTTGATTCCCGGTATTAGAGCAGGTACAATAGCAGGCTAT

AAGCCAGCTGTAAACATATTTTAAAAAGATAAAGGAAGAGAGAGAAGAGCAGCGGGCTAC

AGATTTGTAGCCAGCTGTAGCACGGACTCCAAGACTGTGTTATGGTAGGTGGGATCAGAT

ATTAATAGTATAGTAAACAACTATTATATGAATTGACTATAAATGAATTGGAGCTAGCAA

TGGGCTATGCTATTAAACTGGCTCTTAGAGTAGGTACAATAGCAGGCTATAAGCCAGCTG

CAAACATATTTTAAGGAGATAAATGAGGAGAGAGAAGGACAGCGAGCTACAGATTTATAG

CTAGCTGTAGCACGGACTCCAAGACACGGTGTGTGTATGACAGGTGGGACCAGGTATTAA

TAGTGTAGTATGTAACTATTGTATAAATGAACTATTAGATTAGCTATAGATGAATTGGAG

CTAGCAGTTAGCTATACTATTGAACTTGCTCTTAGTCGTTACCTCGAAACTCGAATTGCC

CGCCCAAAGCTACCCAAAAGGAGTATCGTACTGGTCCACCACGGCCGGTGAAAACCTCTA

CTACTACCTGTACTGGAACTATTAATGCAGAGTGGTTTTTTGATGTTTTTCATCTGTTCC

CGCCACCACCAACACCAGCACGACGCAATAATGGACAGAGCGAGAGCGGTGCGTTTCCAA

TGCCCACTATGCCAGTGCTACGGCCCAGCTGCGCGCCTGCGCCCATCGACCGGTTCACGC

GTCCACTGTGGCGCGTCACATGCCCCAACCCCAACCCCATCACCAAACACCGCACCTGCA

CGCACAAATCTTTACTCCTCCACGGCTCCACACCAATCTCGCCGTCCTCCCCTCCTCCTC

CTCCTCTATATAAACACCACCCTAAGTCCCTAACCCACCCAAACACCACCGCACCCACCA

>LOC_Os03g38210

AACGATCCAACGACTTTATCTGCTCGGTACCAAGCGGTGGGACGAGGATGCGCGGTTCCT

TGGCTCGGCTCCCAGGCCCATCGACTCTCTCCACAATCCCCTTCCTCACGTGTTCTCCTA

TGCCTTCCTCATCTTCCATTGGACCTACTGCAGCGGCATGGCCTCCCCTTGTGGTGTCAA

CTCGAACAAGAATCCCACCCGCAAGAAGGAAGCCTAGGCCATGTTCATGTACACCACCGG

AGCCTACAACGTTCTCCAATTTCTTGTCACGGTTTATTTTGATCATATCATGCGATGGGA

ATGATCTTGATCCATGGATCATCAGTACTTAGTGACATTGAGAACATGATGGTGTATAAC

TAGTACAAGCAGGAAAGAAGGGCTCAAAGGGGACGCACTGCTAGTGGTGGCAATCACAAT

ACGGGTGCCGACACCACAGCGCGCACCAAGAAGTACGCCGTTTGGGACATGGGCAGTGGC

CACTTCAGCATGTTCTCCCATGACCAAGGTGGTCGCACTCCTACGATGGTGGTGCCACTG

GCGTCGCAGATGGATCTTTGTTTTTTTCTACCACAGATCCTCGTTTTCTGCTATGTCCAT

CTTCCTTGTGACGCCGGGTGCTTCTTCTGCTGCGTTGTCCACAAAGGTTTCTCTTTTCAT

GGTGGTTGTAGTCACCAGCGATGTAGTCGCTAGCGATTTGGTCATGCCTGAAGGCGAGTC

GTCCCGTTCCACATCTGCACAATCCCCTTTTGGGAGCCGGGCCAAGGCATGCCATGTGCA

GCCTTGTTGGATAGTGCCAAACAAGGCAAGCTGTTGGATTGGTATATTATCCACGCGTCG

GACATCCGAAGTGGGGCTGCAGGCAATGTGGTTTGCACCCGGACTGCACCAATTTTTTTT

CCGATCCATTGCACTCTCTTTGTCTCTATAGACTCCTCCCCTTTCTCGCTGCCACCACAC

TTCCTCTCCGTGCTTACTGGTGTGACACTTCCATGCACTCTTCTCGTATCTCCTAATCAT

TCTCCTAGGCTCTCCATCCCCACACCACACACCACAGCATTTTGTCCATATTTCAGCCCC

TCTTTTCTCGGTCTACTCTACCATATATATCTCCCAGTAGTCCCCCACATACACCAGATG

CAAGCATAGCACCAAAAGTACAAGAACTCCTAGCATCAGATGCCTCCAGTCCATATATAG

CCCTTTGGAGAGCAAAAAGGGAGAACAACCCAGACAAAAGAGACAGCCATGTACTGCCCT

TGGCCTTAACCCCCACCACCACAGCCCCTCACTGCCTTTCACCTGCAAAAACCACTTGCA

ACCCTCACAAAACCAAAAAACGCCAGCAGCCAGCTTCCCTCCCCCCTCCAATTTTAGCAA

CTTCTCCCTTAAAATCTCCCCCCCCCCCCCCCCTTGCATTGCACCTCTCCTTCTCTACCC

ACACCACCCCCGCCGTCTCCGCCGCCGCCACCACCACCGATTCTTTCTCCGGTGCCACCT

>LOC_Os01g12890

GCCAACGCCAACTCCAACTCCAACTCCCGTACAAATAAAATATACAATCCTTTCTTTTTC

CTCCTCCTCTTTTCCCTTCCATTCCACCCCCCTCTCTCTCTCTTCTCCACTCCAAATCCC

TTCTTACCCTATTCCCCTCCCCCCGCAGCTTCTCTTCCTCCTGCAGTACTCGCCGCCACC

ACCACCGCGCCGCCGCCGCCGGCCGCGTTCCGAGACCCACTCGATCGGAATCCACCGCGG

CGCGCCCGCGCGCCTGCGTCCTCTTCCTTCCCCGGGAGCCGACCGACCACGGCGACCAGT

CGATCTCCCTCTCCGGGCGCCAACCGCGTCTTAGCTTCATCGAATCCACCGCCCCACCCC

GCATCTCCTCCTCCTCCTCCGACGACGACGACTACTACTAGTCTTCTCCAATAAGCCCCC

CTCCCGCTCCCCCCGCCTGAAGAAGAAGCAGCAGCTAGCTCCGGGGAGAGGTCGACGGCG

CGCCGGGTAGATCGCGCCCCGCCCCGCCTGCGTCGCGGCTGTCGGAGCAAACGCAAACCC

CCCAGGTAATCAACGAACTTTTCCTCCGCCGCAAGAACAGCTCCCGCGGGGGGTTTGGTT

TTGACCGATTTCTTCCCCCCTCCCCCCAAATCGACCCATCCAATTTCGCCTCGATTTACT

TCCGATTTCCCCACTTTTTTTTCTTCCTTTCGGGTTGGGGGGTTGCGGTTTTGGGGGAGG

AGAGGGGTTCAGCTCATCCGAAGCCCCACGTTAGGTCCGCCCCCTTTCCAGCTGTGCCCC

TCTCTCGGGCCTCGAGCTCCTCGCCTCCATGGGAACCAAAGCCCTTATATTTCATGTCGC

GGAAGAAAAAAAAATCCCGTCTTTTGCGGGGATCCTCGCGGCTACGTACGAGCCCTAGTT

ACCGCGCGGATTTTAGTTACGGCGGTTTATGCGGCCCCTCCCTCTAGGTTTTAGATCTAC

CCATCTCTCTCTCTCTCTCTCTCTCTCTCTCTGTGCATGCATGTGTCTATCTTAGCTATA

CCTGTATTATTTGGAAGGTTAATTATGGTTGTGTATATGTGGCGCGGTAATTAATTAGTT

TAATTCGCACCCCCTCTCTCTTTGTTTATCTAGGTTTTGGGGGAATTTATTTCTTGCTAT

AATTTTGCCCGCTCGAATTTCTGGTGCTCTTATATTCCATGAGCTGATTGAAGTGGATAT

ATATTGTGCGTGCGTGCGTGCTATTGCTACATCGGCTTGACTTCTTCTTGCCTACTACTT

CATTAATTTGTTTCTTCTGGTTTCTGTTTCAGGTTGTTCTAGCGTGTGCAGCGGCTAGCT

GATTGATTGTCTTCTGTGATATATCCAGAGCTCGTGTTTTGTGGTTTGTGGTTTGTGGTT

TGTGCTTGGATTGTTGATGTGCTAATTCGCGGCGTTACAAGATCACTGCTGGATTGATAT

TGAGTTGTGCCTCGGCTGTGCTAGCTGTGTGTTGATTCTCTCCTCGTCGTGGTGATCGAT

>LOC_Os02g47280

GCTCTCCCGTTTTATATTGTTCATCGTATAACCCAAAATCAGAATTTCCAAATTATATCT

TGTAATCTTGACTGCATCGTTTGACATACAATACTATTAAATCTATACATATAAATTGAG

TCTGTATACGTATATACAAGCACTTGAGATGGTTAAGCACTTTTTTTAGCATTCTAAGTT

TCTTATTTTGTAGGATTTTTAGTACGAGGTAAGACATACTTGAAAAAAATTATAAGAACT

AGAGTGCATGTGACCACCTAACTCCTTGCAATTTTTATTCTTATAATTTGAAAATCCTAT

AAACCAAATAAGCCCTTCAAAGGAAATTAAATCATGAGGTTTGAGGTTAGGTTTGAATTC

TCTAAAAAGTGGAGGAAAGGACTCAACAGAAAAAAAAATCCTATAGAATTTCGATCCTAT

AAAATTTTAGTTAAAAATACTTTGTTCCAAAATTGCCATGGATAAAATGTAATTTCTATG

CATACAACTAAATTATCGATGGCAACAGTGCATGAGCATATATTTATTTCATTGACCTAC

GGTTGCATGTCTTCGATCTCTATGGAGTAGTACCGAGGCTAAGTTTAGTTTCAAACTTTT

CCTTCAAACTTACAGCTTTTTTATCACATTAAAACTTTCCTACATATAAACTTTTAACTT

TTCCATCACATCTTTCAATTTCAACCAAACTTCTAATTTTAGCGTGAACTAAACACACCC

TGAATTCAAAACTCTTTTTATTTTCCTTCAAGATGTCCGATGCACACGCTCTATGTAGAC

GCAAGAAGATGTTGGAGCAGCAGACTAACAGTAGCAAAAAAATGGCAGGTCGAAAAGCAA

CTGCGACGGTTGCTCCGTCATCCTCTCATCGCCTTTTTATTGCTCCGGCGTTGGGAACCG

CAACAATGGAACAGCCCAAATCGACAGTCCCCTCCACCCCCCTCCCCCATCCTCTCTCCC

CCCACGCAATACTTGTCACTACTCGCGCTGCCCACTACAGCGTCTCTGCATGTATATCCA

TCTATCCATCCATTCCCCCATTTTCCAAATAAAAATACAGCAAACCAAACACAAACGCAG

CCTCGCACTGTACTCGAAGAAAAATCGGTGCTGTACGTACTACGCCACGAGATAACGAGA

GAGAGAGAGAGAGAGAGAGAGGAGAAAATGGAAATGCTACTGCTCGTACCACGCCGCTAC

GTCCGCTAGGTCGACAGGCCCGGGGGGAGGCAGGTGTTTGTCGTCTAGCTCGGGTCGGAG

CGCGCCTTCTCGTGTCGGGCTCGACGTCCGCGACTCCTCGCCCCTGGTCGAGAGCTCGCA

GGCGCAGCGGGAGAGAGAGAGAGAGAGAGAGAGAGAGAGACAAGCCGCGCAATAAAGGCG

CGCGCGCGAGCGAGCGAAGCAAAGCACCATTACTAAAGACCGCGGCGTGTGCTTGCGTTG

CGAGCGAGCGAGAGCGAGAGAGAGATTGAGAGAGAGAGAGGGAAGGGATGGCGATGCCGT

>LOC_Os04g54900

TACACAGGACTCAAGCTAGCAATCATGGTTTTATATTCTCCTCCCACACAGCTTGAGACA

ACCCTAGAGTACAAATCTTCCAGCATCCCAAAATGTTCCCTGCACGAGCAGTCTTAGTGT

CAAATAAATTATGCAGTGGAATATGTTTTAAAACAAGATAATATGTGTGAACAATAGTGA

AACAGGTACATACTGAGCCAATTATTAAAGGGAACTGTTAGGAAGACACACCTGGATTTT

GCTTTGGATGCAGTGGGCCAGTGGAAAGAGTCTACCCCCATATGCACCTTGGAAAAAAAA

ACCCCCACAAACACCTTTGCATTTGAAATAATAATCATTGGAAAATGTCTTTTTCCCTTT

CTAATAAAATTTGGAAAATACAAAGATGTATATGGGTTTTAATTTTCTTCCCTTTTCATG

CTATTATTTAAAATAATATGAATGCCGCATATTTATTGTATTTGGTTTAAATAGAATGAA

TCAACGGGTACCCATACGGAGTGACTTGCTCTCCCCGTACTGAAAACAACTCTCAGATTT

TGGAAACAAAAAGAACATCACGATGCTAAGCATTCTTCTGTTGTCGTTCTCAGTTACTGT

GACTTGCCCCATACCATCCCTCTATTTTCTTATCAGTCGCTATTGCAAAAATTCAACCAG

AAGCGATGTACATATGGAATACTTAATTAGCCTCGTTAAAAATTTAAATTGTGTCGAAAT

ATTATTAGCTAGGTAATGCCAGTGAAATTGCAATACTGTTTTTTTAGGTTCTGAAAATTA

AAACTGTTTTTTTACTCAGTACTACAAGATTTTTAGATAATGGATTAATACTACAAGGAT

GCTTTATTCTTTGAGAGGAAATGCTACAATAATTAAGATGCTACTGATCGATGACTAATC

TATTTAGTGATGCCCAGCAGCTAGCATGAACGGCATCGATCTGCAGATCCAGGGGAGGGA

GTGCAGATTCAGAGAACAGCACAGGGCCGGCCAGAAAACCACACACATATAGTACAGAGT

ACAGACAAATCCAGCGGCCAAAATGCAAGTACGAACCAAATAAAAAGAAGCACGCACACA

CACGCACACACAGGCTGCAGCTAGTAGCTCTCTCCTCAGTCGTCCTCGCACCGCAACACA

TGCTCAAGACTATTAGTCGATTTATGCTCGCAAGTCATCACAACGTCTCTCTCGCTGTCG

CACAATTACAACTCTCGCTTTCTCCCTCCCTGCTGCTGGCCTCAGCCTCGCCTCTCTCTC

CTCTTACTCTCCCAGCTCCGCTTCCACCTCCCGGCCTCTTGCGGCGGCCTAGCTAAGCAA

GCTCTCGCTAGCGGCGCCCGGCCATTATATATAGCTCAGCTCGCCTGCAACTGCCAATCT

CAGGCATCAAATCTAGCTATAAGCTACGCACACCTAGCTTGCTCTTCTAGCTCAACTAAG

CTATTAGTACTATATAAGCTAGCTAGATAGCTGCACTGCAAGTGGTTGTAATTTGCAACC

>LOC_Os04g56500

GTTTATAAATAAAACTTATATATATTTGTTGTTAGTAATTTAAAACTTAATGTTGAGAAA

TAAACTAAAAAAAACATCAAAAAAAATCTAAAATTAAGATTTAAAATTTAAATTTCAGTT

ACGACTAACAAAGCTAAAAAGCAAATGATGTAGCTTAGCTAGGGTTTAAAGTTTATCCAT

AAATTGATCTCTCCTACAATTTCTTCGATCTATCCAACACCGCTATATTTATTACTCACT

GTGGTAATATTATATGCTCCTAGAGGCATTGGAATATTTTTTTTTTCTTTTTACCTCAAT

TTTAAAGCTTGCCAATTAACCTAAAATCTCTTAAAATTTGAACGGATCAGGTAGCTACAG

AATTAGCCACTGCCTTGTTTATCTCTCTAGGGGATTAGCTAGCTAGGGGCCTGTACTGTA

TCTATCTCAAAACGCCCTAACGGGAACGGAACGAATCGGCATGGAGGCGCATGTTACCTT

TCTGCAAAGCGTGTGGCATGTGTTGCCCAAGATTACGATCAATTGGTCGATATTTGATCG

AGATCAAGTTGTTGCCTGGCTCCAGCAACATCTTTATAATCTCTCTGCTGGATTAATTAT

TTTAATTTTTCTCCAGCTGTGCAATATACACACACATTTGCGCGTAATATGTGCTTTTGC

ACGCCGATTAGGCGATTGCTACTATAGAAACTTAATTAACATTGCATGGCGATGGTTTGA

CGAACGTGTCAAACCCCAAATCCCAGGCCGGTTTACTCGTTTTACGTTGCTTCCACGCAG

ACGAACAATTTAACAACAAATCGAGTTAATGTTTCAGACGCGGACGGCAACGTGATGCTG

ATGCTGATTTGCATGAAAAAAAAAAGCATTGCCTTAATTTTTCCTCTTTTTTTTCATCCA

ATTGTAACTACCACTACCACCGTACTCATATATCTATCCAGCACATGCATGGATATGCAT

GGTTAATAGTAGTTTAGCATGTGTTGTTTCCTCATGTTCGTCAATGCAATCAGGTTAATT

AACCAAATCAGCAGATTGCGTAGGAGTAGATGTATGTGACCGATCGAGTAACTGAATCAA

TATATCGATCCGACCAAAGTCCGACCAGATTGCTTTTATATCCATTAGCCCACATGCAAG

CAAGAGCTAACCAAAGATGAGTCTCATGTTCATCTTGGCCAACAATTTCTTACTTTCTTT

CTCTACAGTACTAGCAGACTAATTTTTTTAATAAAAAAGAATAATTTGGATTGTTTTTGT

CTCTCGTTGTCAACTAGGTATTATAAGACACCGGATCTTGCTCAGGACATTTGTGTCAGA

GCCGTGTTCCTCGCTCTCTTCCCTTTCCTTTTTTTCTCTCTCTAAAATCGCAACCCACAA

ATCCTTCTGATCCGTTTGTGTCAGAGCCAAATCTTCTTCTTCTTCTCCATCTCTCTCCTC

TCCTCTTCTCTTCTCTCGTTGGAAGGTGAGGAGGTGAGGTGAGGTTGGGGGCGCCGTTTG

>LOC_Os02g54600

TTCTAATTCAAACCAACATTTCAATTTTGCATTCTAGGTGCCGTTAATAATGTGTCGTAG

CCGTTTTCAGAGTGTTTTTATATGATAGTAGTGTATTTAATAACTACACGTATACTTTCT

CATGACGCTATGTTTAAAAGTTGGCGCCAATCGCAACTAAAAGTAACCTCACCCAATTAT

CTATTAACTTATTAAAGGAATAGAAAAAGGATCATCCACGTTCGCTCTCACGGCCTAGAA

ATTCTTACATTAATTGAAGAAAAAAGAAAAACAGAGTCCATATAGAAATACAATTTAGAA

ATAGTTGAAATTCGAAATTAAAAAAATAAGGAATATTGGAAGAGGAGACTAGAGTCCATA

TATAAATACAATTTATAAATAACTGAAATTCGGAATTAAAAAATTAAGAGTATTAGAAGA

ATAGTATAGAGTCCATACTATTAAGAAATAATAGAAAATCAGAATTAAAAATAAGGAATA

TTAGAAATAGAGTATAGAGTCCATATAGAAATACAATTAAGAAATTATAGAAATTCAAAA

TTAAAAATAAGGAATATTAATAGAGTCCATATAGAAATTTAAAACTAACTATTCAGAATA

AACATAATAGAATTAAAAGTAGAGTTTAGAGTTCGTATAAAAATACAATTTACAAATAAC

TAAAATTTAAAATTAAAAAAACATGGGAAGAAGAATATAAAGTCAATATATGAATATAAT

TTAGAAGTAACTGAAATTCGAAATTAAAAATTAAAGAATATTGAAAGATGAGTTTAGAGT

CCACATAGAAATACAATTAGAAATAATAGAAATTCAGAATTAAAAATAAATAATATTAGA

AGAAGTGCCTAGAGTCTATATAGAAATACAATTCACAGAGAACAAAAATTCAGAATAAAA

GAAAATAAAATATTGAAAGACGAGTCTAGAGTCCGTATAGGAATATAATTTACAAATTAC

TACAATTTGATATAAAAAATAATTAATAACTAACATGTATATAAAATACAATATAAATAT

TACACATTAGTAGTTTCGTAAAGTTAGTACAAAATTTAAAATTATGTTGTTATTTTAATA

TATTAGATTGAGAAAACATACATGCTATTATATAGGAGAAAATATAATGATGCTAGCCGC

GTAATCTGCGTAGGAATCCTGCTAGTTTTTTTTACACCAAAAAGAAAATTTACCGAGGTC

CACAAAACGTTGAACTATCACTTGGAACACGGCAATACGAAACACAACAAAAACTGACTC

GTCTGATTTGTAAAACCAGAGAGAAAAGAAAAAGGGGGAAAAGGAAGTTATAATTTTCTT

GCCACCCCCATCACGGCTTCACTTGGCCTCGTCTATAAAAAAACACTCCTCTGTCTCGAA

TCCTTCTTCCTCTCCCTGCCCCCGATCGGCCTCGTCCTCTCCACGCCGCGAGCCCACCAG

AAAACGCACACGCCGACGCGAGCCAATCAACCCGCCGCGCCGCGCCTCGCCGCCGTCGCG

>LOC_Os06g06090

AGTAAACAATGTTATTAGTACTGATGCTTATTTCTTTATCTCCACTGGATCAAGAAAAAA

CACCTTTCTGTCTCAATCATTTGTAGGAGTATGAAGTCTTCGACGAAAAAAAAAAGTGTT

GGTAGAATGACATGAGCGGGAAGATATGGCGAGCAAAAAAAAATATGGAAGAAAATGACA

CCATTATCTGGCGCTAGATAGCATTCGCCGCCCTCGTGTTTGGTGTTGCTGAAGCAACCA

CCTTAAGCAAGGATAATAGTAAACTATAAGTTTATATGGAGAAGGAAGACAAGAAAAGTA

CAAAGCTCTAGTCATATGCTTCAAGGTACATGCACTAAATACATAAGCAAGAAAAGGTGA

AGACAAATGTTATAGCCAATCTTATACTTGTAATATGAGTTTATAAGTAGTTAGCTCTAC

CATTCAACTTGAGTAAAGTGCATTGGCGGTCCTTCATCTTATAGGGTTGTGTTATATAGG

CCTCTAAACTCTCAAAATACATATCTGAGTCACGTAACTTATCAAAGTGTATCATCTAGA

TTCTAAATTGACACATCACCTCTAGGATCCTACGTGGCGTTGATGTAGCATGCCACATGG

ACATGATGTGTCCTTTTCTTTTTTTTCTTCTTTTATTTTTCTTTTTATTTTCCATTTTCT

TCTCATTCTTCTTTTTTTCCTTTTAAATTTGTAGCCAGCTGCAGCACGAACTCTAAGACG

CAATGTGTATATGACAAGTGAGATCATATATTAATAGTGTAGTATATGTTTATAGGTAAC

TATTGTATATATTGGCTATTAAAGTAACTATAGATAATTTGGAGCTAGTAGTTGGTTATA

CTAATCTTGCTCTTAGAGCAGGTACAATAGCAGGCTATTAGCCAGCTATAAACATATTTT

AATGAGATAAAAGATGAGAGAGAAGAATAGCGGACTTAGATCTGTAGCCAGCTGCAACAC

GGACTCCAAGACGCAGTGTGTGTATGACAGGTGAGACCATATATTAATAATATAGTAAGC

AACTATTGTATGAATTGACTATTATGTTGGCTATAGATAAATTGGAGTTAGTAGTGGGCT

ATACTATTAAACTTGCTCTTATGGGTGTAAAGGTACTAGGTACAGAGGTAGTGTTCCCTC

ACGCTGTCGTCACCATGACACGTCATCAGTCGTTAGGGTCGGCACAGCATCTCCATTTTG

CTACACGTCCCCCCACACCCACCCACCCCAAAAATGGGCCACGCCTAGTACCCGTAGCCC

CGCGTGCCAACCATTTTATACCCCGGTGGGCCACCACCCCCACCTCCACAGCCCAAGAGA

AAACACACGCGAGCACACACACACCCACACACAAAAAAAACACACACAAAAAAAAAAGAG

GCGAGAGCTACAAAACGAGGCCGAAAGCGACCAAATCTCGCGACGAATTCCGCCTCCACT

TTCCCTTCCCTTCCTCCTCCACCTCCACCTCCTCGTCGCGATCCAAATCCGAATCCGGCC

>LOC_Os12g13380

AGCCGGCACGTGGAAATCAATGCTGCAGGAAGATTAATTAGGTGGACACACCAAACCCTG

TGGTTGGTGACGCCCTGTTGTTAATCAACTGGGGTGTTCGTTGGACATGGTTTTTGCAGG

AAAATTAAGCAAGAAAATTAAGAAGAATGCTCAAGCTGACATGAGAAAACGTAATCCAAT

GGAAGCGAATTTCAAGTCGTTCTCTTGTACTACCATGTTTAGAATACATAAGACAGTGCC

AACGGTTTGATGGCTCCTATTGGCTCGTGTGATACTGACTTGTGTCACAAAGCATCAAAT

TGCTTCTTGGAGTATCTTTATTACCGAAAACCCCAAAGATTATTCTATTCCACCTCAGGG

TAATTGTGCTGAACTATGCAATGAATACAAATTCGCAAAATATCATGGTTATCTATCTTG

CTCAAATTGAAATTTGAGTCCAACTGAGACTGCAATACGATTTTTCTTTTCAAAAAGAAA

TTATTAATTTTTTTTTCATGAAACGCAATTCAACCGTTCGAGAAATATGCTGTCATAAAT

AAGTAGTCTAGTGCAGAAACAAAATTAATATCACATAAAAAAGAAGGTTGTTAATTACAA

ACCATGTTTCGTACTACAACTCTAATTTGTAAATTCTTATTTCAGTCACAAAATTCCAAT

TTCCAATTAAGAAAAATAAACGTAGACGGCTAAGCCCACCCATCTAAGGCTAAGTTCGAG

AGGTGAAGTACGCACGAAAAATATGATGGTTTATTAATATGATTTTTTTTAAATAACTTT

CACATAAATTTCTTTAGGAAACATATCATTTAATGGTTTGAAAAACGTGCACATAGAAAA

CTAGAACGATGAGTTGGGAAACAAGAGAAAAACACAGCCTTAAGGCTTCTTGATCCTCTA

GTTGGAGGTTGATTTTCAAACGCATGATAAACGAGAAAGCTCATTAGCACATTATTACTT

AGATATTTATAATTATAAACTTGAAAAAAATATTTATTTGAATTTTTTAAACAATGTATG

CATAAATTATTTTTTAAAAACACACCAATTTAACCCTTTAAAAAGCATCCTAATAGGAAA

CGAGGAAGTTAAAGATTCACCGAAGGTGTGTTTGGATAATGGAAAAATGGGGTGGGATTA

GAATTGGTAAATGAATCAGGGTTAGGATTAAATATTAAAATGAAAGAGGGAGAATGAATG

GTTAGAGTTTAAATGTGTCTTTTTGGTGGGTAGAAAATTATTTGCCATACACTCCCCGAG

AGCGGTGCGTGCTTGCGTGGGCAGAAGCGTCTTTTTCGTTGGAAAAAAAAACTGCTTAAA

AAGGAAACAGAAAGAGCCCAGCTTTGGTTGTCACCGTCTCACCAGAAACGAAACAAAAAG

CCCCACCACCTAAACCTCCTCGATCCGACCGAGACTCCTCCATTTCAGCGGCGCACGCGG

AGAGCACGCGACGCGAGTTCGTCGACGAACAAGGCTAGTGCAGTAGTTGTTGCTGCGGAG

>LOC_Os05g06280

GCGCGCACTCCCCAACTTGTTATCATGGTAATCACCAAACCACTAATGACACCAAACGCTAAGGCAAAACAACCTAGCCAAGCCATCATATTGCTAAGCTACACAGCTAGCCAGCTCAATATCCTTTTCAAGAACCTAATTTTTTTCCTATCGCTGGTAATCCATATATTGTAACGAGTCAGGCCCATATATTAGGATGGGTTAGAATGGTGAATGAGAAATGTTGGATCAAGTGGTTACACAAGTGAAACAGACATGCTATGCAGGGTGAGGAAAGAGTGGAAAAAGGATGACTATGTTTTATCGTAGTAGAGATAGATGATATATTATATATCATGCGCACAAAGCTTAGAAACAATATGATATATATATATATATATATATATATATATATATATATATATATATATATATATAAAATTGTAGAATTATTTTTCTAAAACAAACTTTTTAAAACTTATCTGCACCTTGAATATCATCACAAAATCAAATAATCTGTCTTATCTATTCATACAATCAAACCATGCCTCAATACCTCCCGGAATAAAATCTATCATTGCCTTATTTTATTTTCAAGTTATTCACCGTATTATTGAAATGGTTTTTTTCTCCACATTTTGAAAATTACATTTTCTTAAAAAATTCGTTAGACCTTTTTTTTCTAACTTATATACTGAGTTTATTTACCTAATTATTTAATTTATCCTATGATAAAACATATTACCTGCTATAATAATCCGGTCACTAATCAATGTCAAACTGGGGCCTATCTATTTTGCTTCAAATTGAAAAACTCCACATTTCAAAATGCTAAAAACCACCCCATTTTAAATTGAGGTGGTTTATCTCAAATGGAGCCTACTTCCTCTGTATCACGATAAAAGAAACTAATATAAGATAAGATGTACTACGAATCTGAATAATAAATATGTCATATCTTAGGTTTGTTTATTCTAAGAGAGCGTATCTACTTGGGATTCAAATTGAAAAACTTCACATTTCAAACTGCCAAAAACCACCCCCATTTTCTCCCTCTCATTATCTCCTTAAAAAAAATCAAAAAAAGAGAAGAGAGAGAGAGAGAGAGAGAAAGTGAGAAAAAAAAAGAGAGATAGAGAAATAAAAAAGAGAAGAAGGGGGGAGGCCAGCTGAGGCAGGCACCCCACCACCCTTCTCCCTTCCCCCAATTCTCTGCACGCCCTCAAACGCGCCGCCCGCATTCCGCGATCCCCCCCCCTTCTCCCCGCAGATCTGGGATCCTCCTCCGCCGCCGCCGGATCCCCCCTCCGCCGCCTCCCGTGCCTCGCCGCGCCGCGTGATCCGATCCCGGCCTGACCTCTCTACCCCCCCGCCGCCAAGTTGGCGCCTCAGCGCAGGTCGCAGCGCCTCGCCGGCGGCGGCGGCGGCGGCGCAGGGGCCGCCGCGCAGCCGCCGGGCGCGCAGCAGCAGCAGCAGACTGCGCCCGGGGGCGCGGGGATGGGGGACTCCGGGGACGCCGTC
